# Supplementary figures and images for: An AGO10:miR165/6 module regulates meristem activity and xylem development in the Arabidopsis root (part 1 of 2)
Source: EMBO J. 2024 Apr 2;43(9):8. doi: 10.1038/s44318-024-00071-y (PMC11066010; doi:10.1038/s44318-024-00071-y)

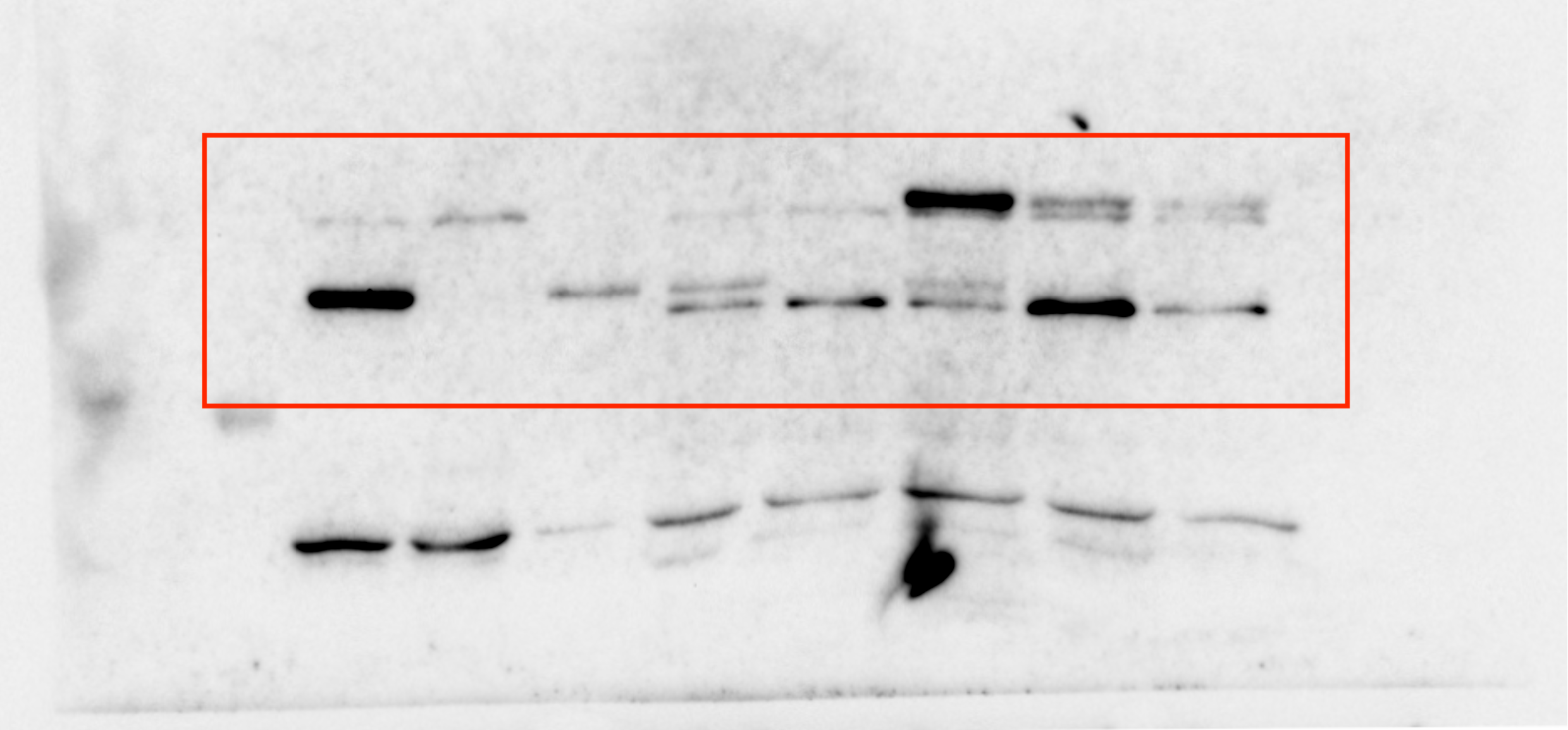

Supplement: Supplementary file 4 — Source Data Fig. 1 [file 44318_2024_71_MOESM4_ESM.zip › Fig 1/B/Figure_1B_WB_AGO10.tif]

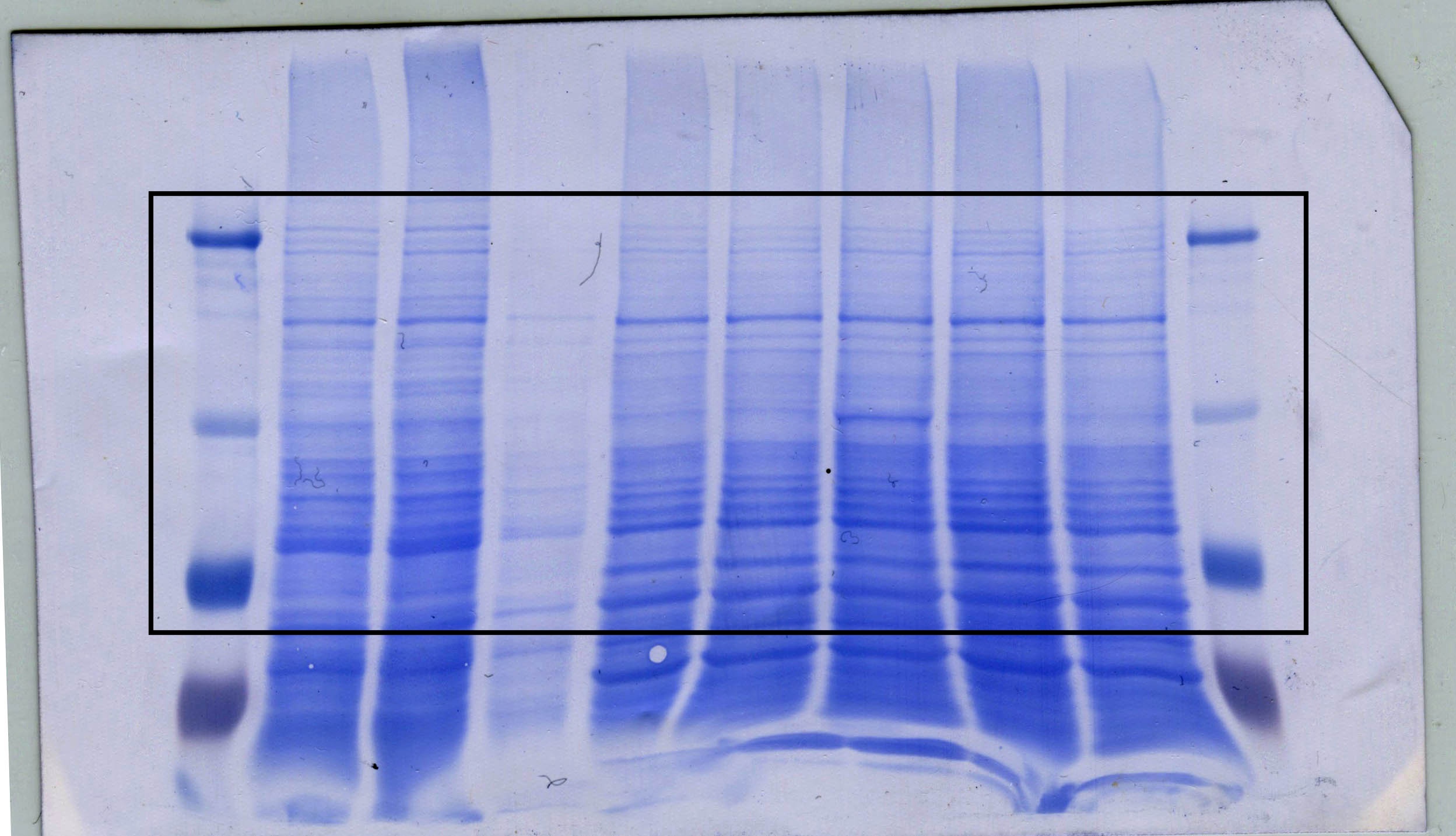

Supplement: Supplementary file 4 — Source Data Fig. 1 [file 44318_2024_71_MOESM4_ESM.zip › Fig 1/B/Figure_1B_commassie_WB.jpg]

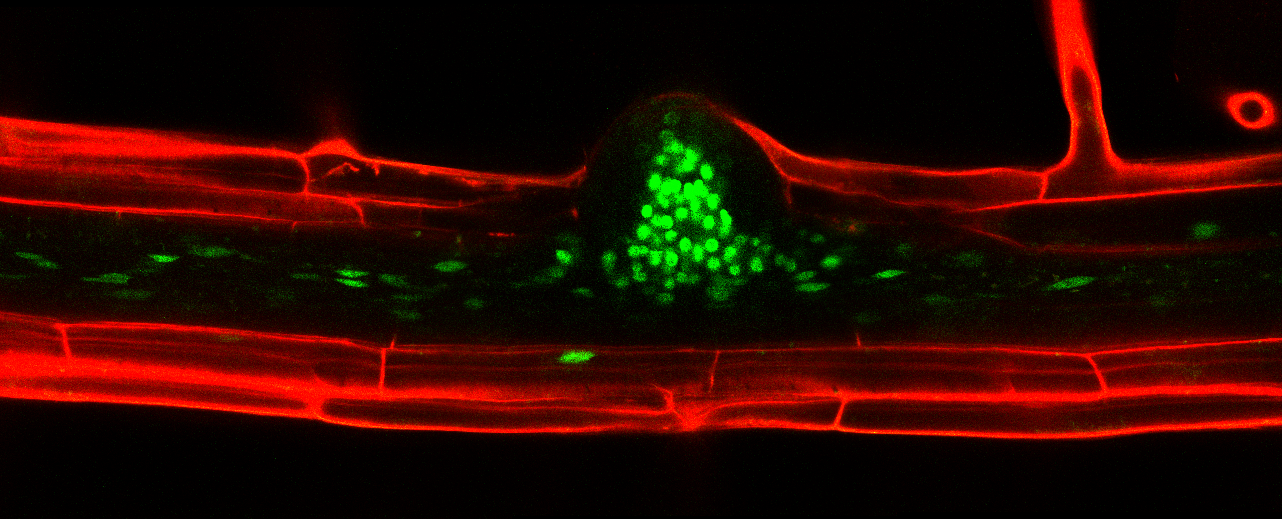

Supplement: Supplementary file 4 — Source Data Fig. 1 [file 44318_2024_71_MOESM4_ESM.zip › Fig 1/D/1D-A10H2B lateral root2.tif]

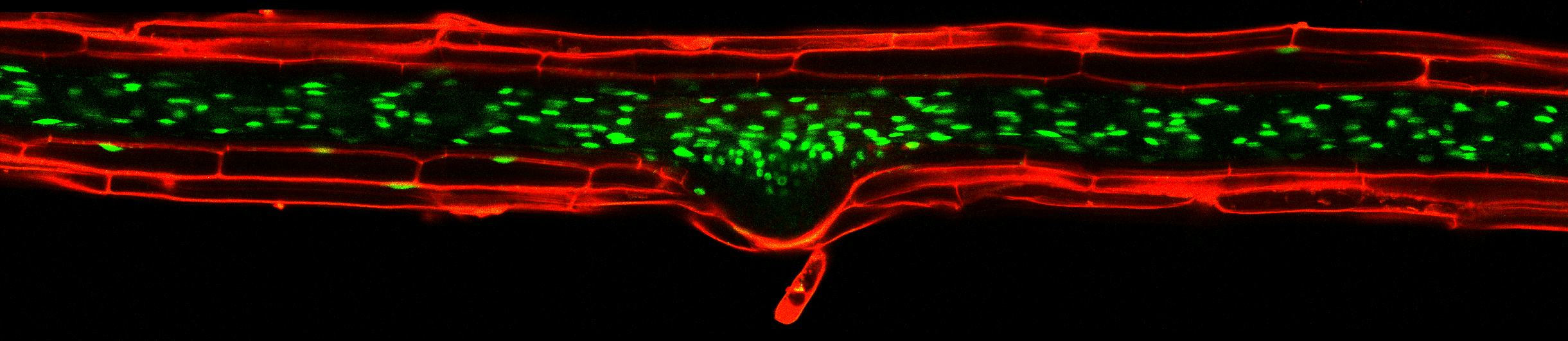

Supplement: Supplementary file 4 — Source Data Fig. 1 [file 44318_2024_71_MOESM4_ESM.zip › Fig 1/D/1D-A10H2B lateral root1.tif]

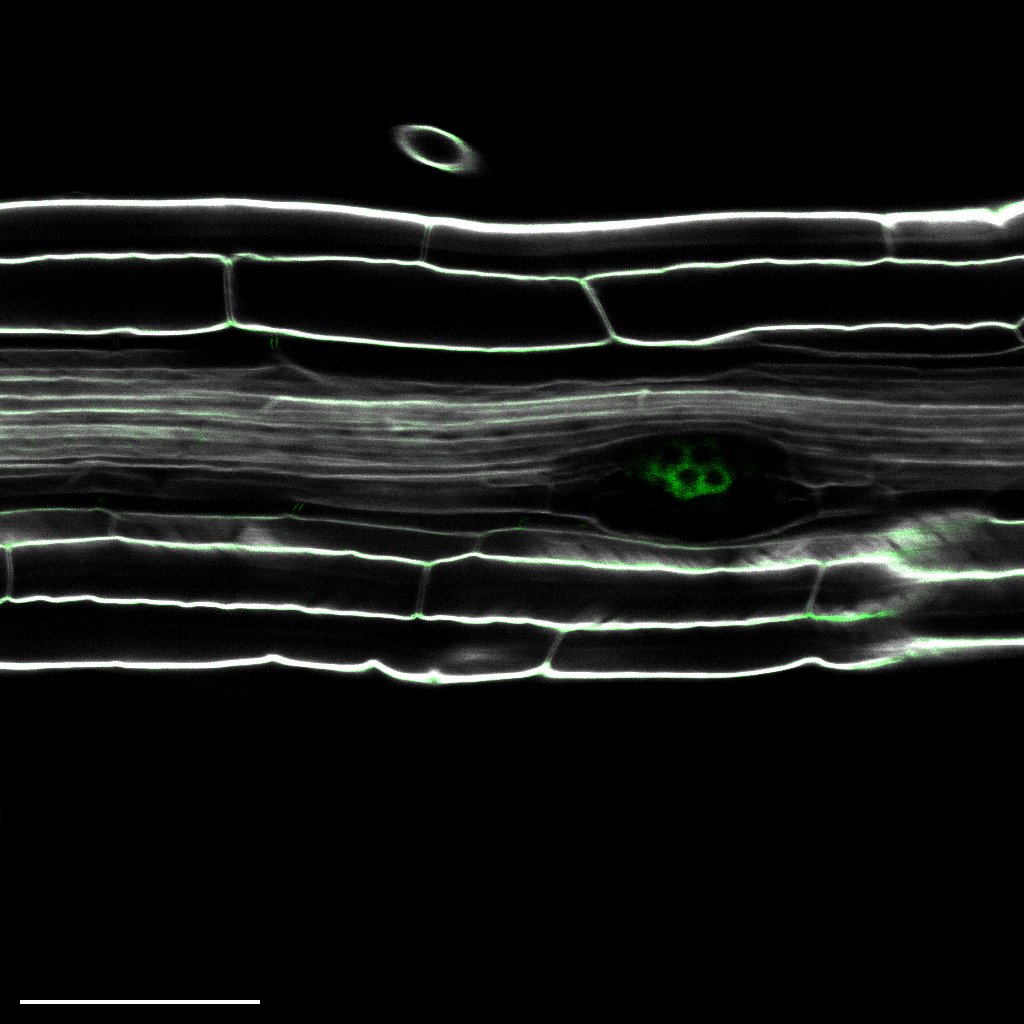

Supplement: Supplementary file 4 — Source Data Fig. 1 [file 44318_2024_71_MOESM4_ESM.zip › Fig 1/D/1D-A10 lateral root-1.jpg]

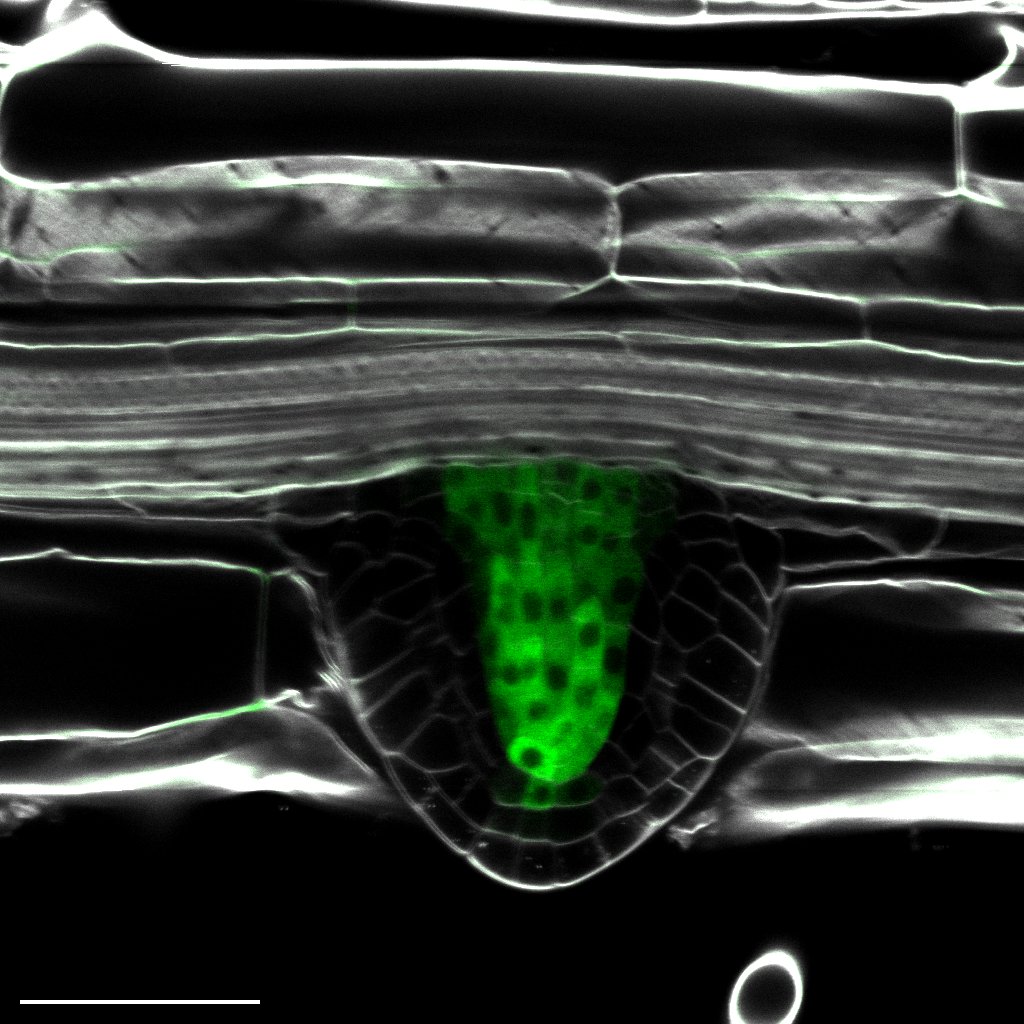

Supplement: Supplementary file 4 — Source Data Fig. 1 [file 44318_2024_71_MOESM4_ESM.zip › Fig 1/D/1D-A10 lateral root-2.jpg]

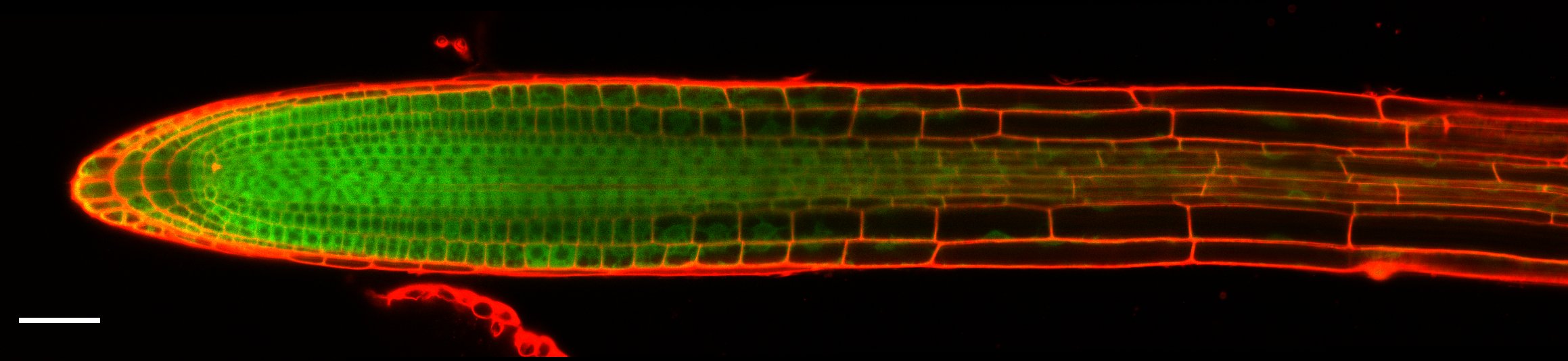

Supplement: Supplementary file 4 — Source Data Fig. 1 [file 44318_2024_71_MOESM4_ESM.zip › Fig 1/A/1A-A1GFP.jpg]

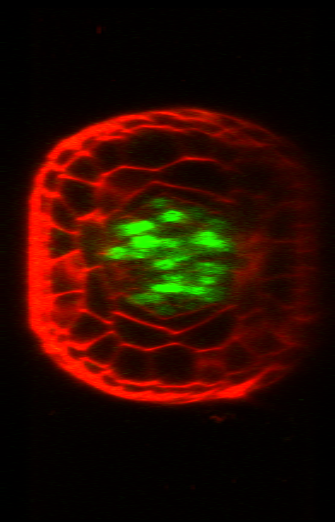

Supplement: Supplementary file 4 — Source Data Fig. 1 [file 44318_2024_71_MOESM4_ESM.zip › Fig 1/A/1A-A10H2B-CS.tif]

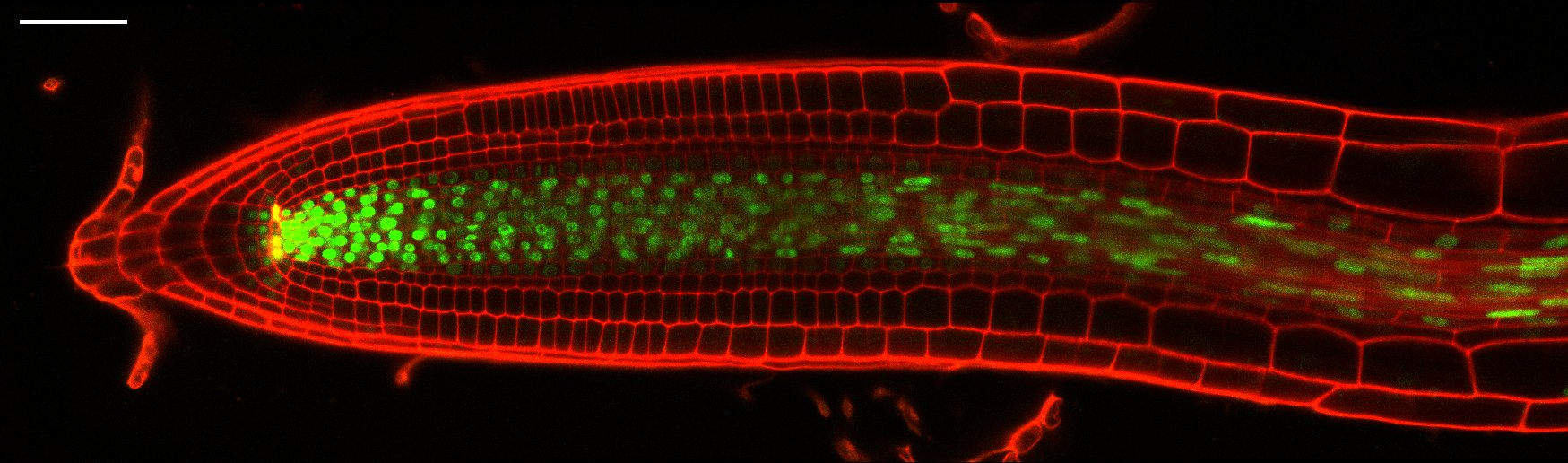

Supplement: Supplementary file 4 — Source Data Fig. 1 [file 44318_2024_71_MOESM4_ESM.zip › Fig 1/A/1A-A10H2B.jpg]

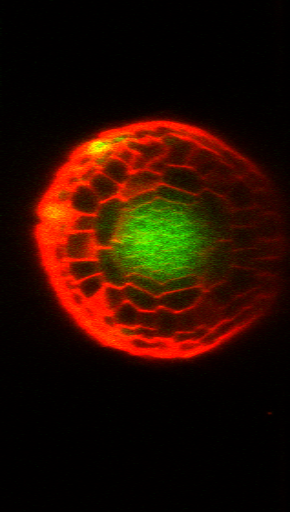

Supplement: Supplementary file 4 — Source Data Fig. 1 [file 44318_2024_71_MOESM4_ESM.zip › Fig 1/A/1A-A10NTF-CS.tif]

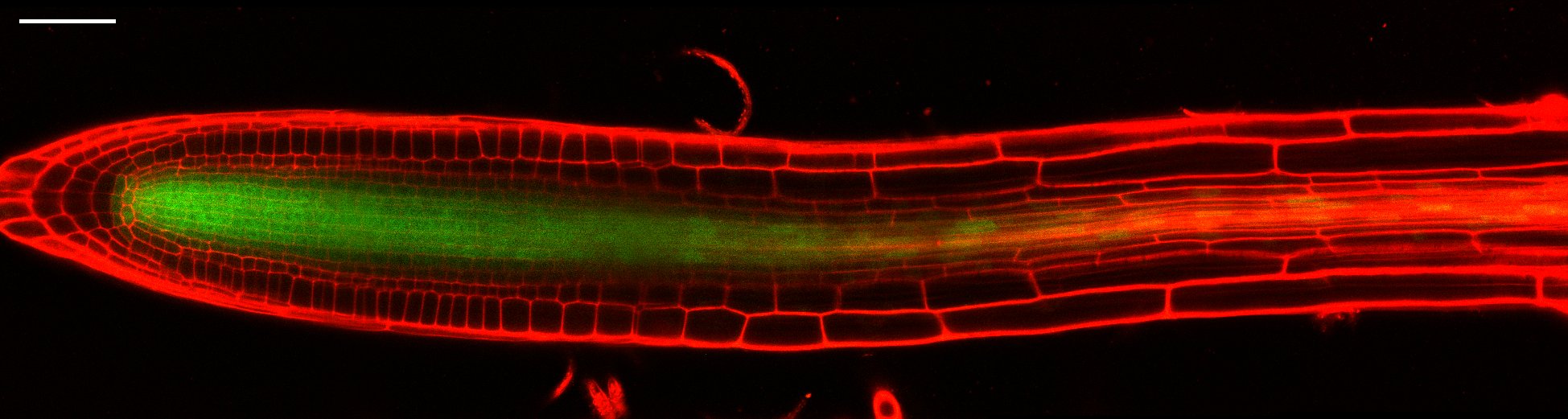

Supplement: Supplementary file 4 — Source Data Fig. 1 [file 44318_2024_71_MOESM4_ESM.zip › Fig 1/A/1A-A10NTF.jpg]

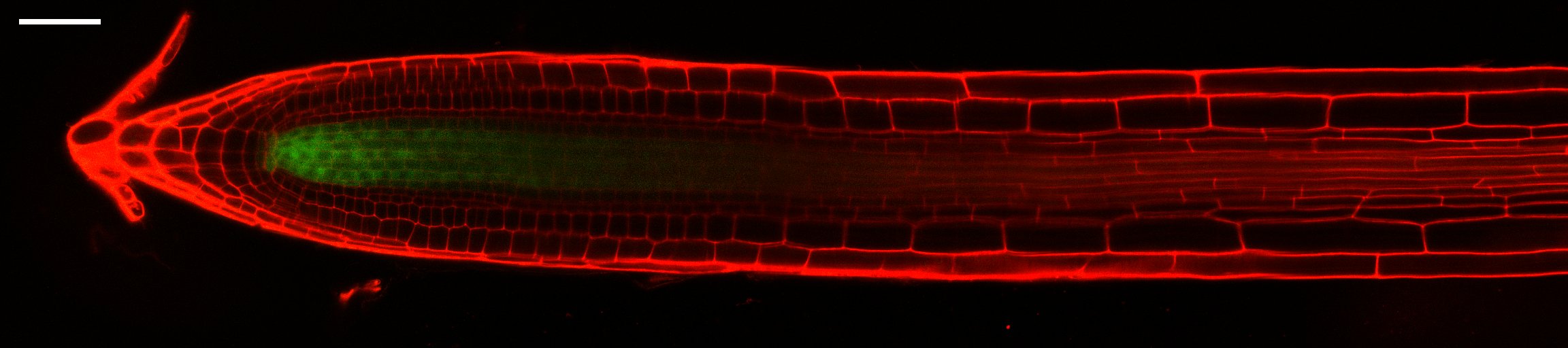

Supplement: Supplementary file 4 — Source Data Fig. 1 [file 44318_2024_71_MOESM4_ESM.zip › Fig 1/A/1A-A10NX.jpg]

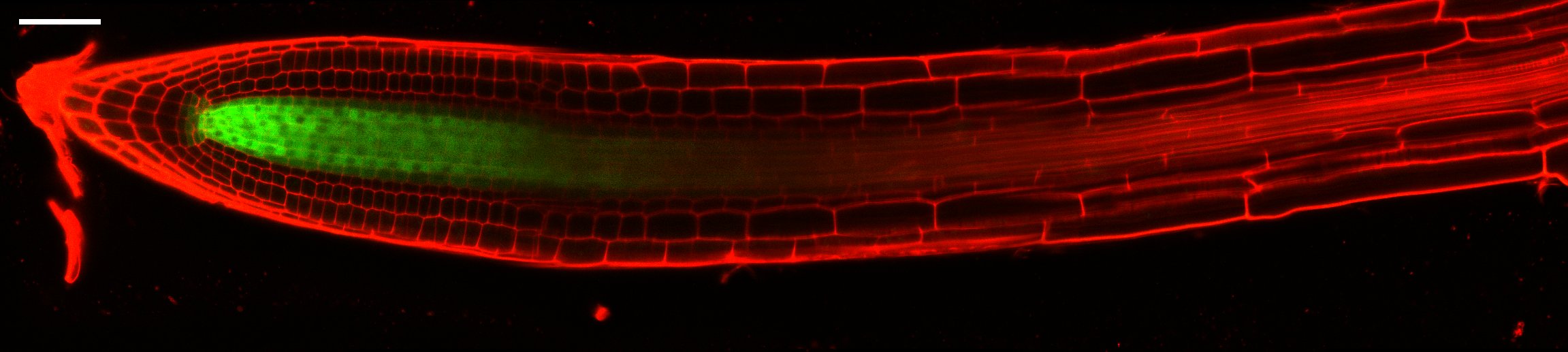

Supplement: Supplementary file 4 — Source Data Fig. 1 [file 44318_2024_71_MOESM4_ESM.zip › Fig 1/A/1A-A10OX.jpg]

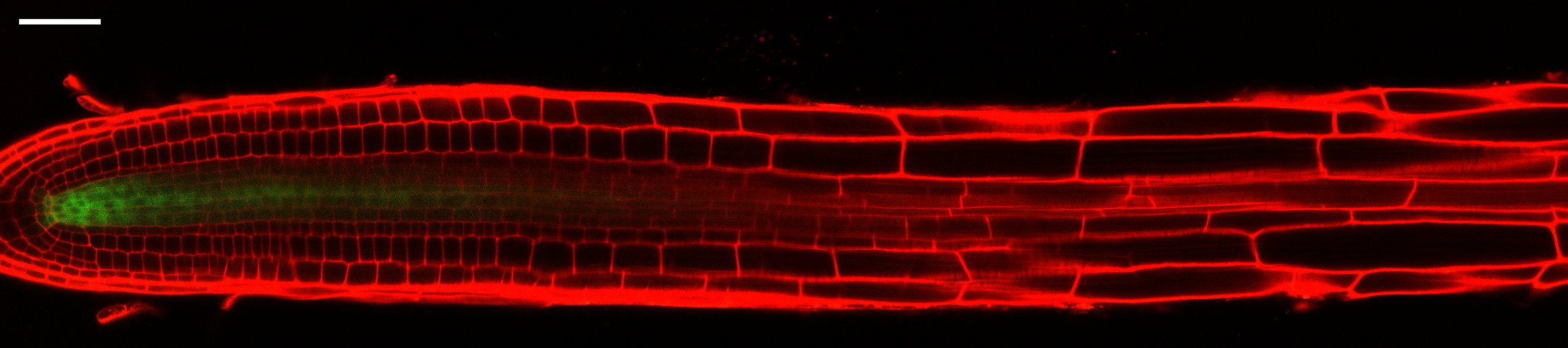

Supplement: Supplementary file 4 — Source Data Fig. 1 [file 44318_2024_71_MOESM4_ESM.zip › Fig 1/A/1A-A10UX.jpg]

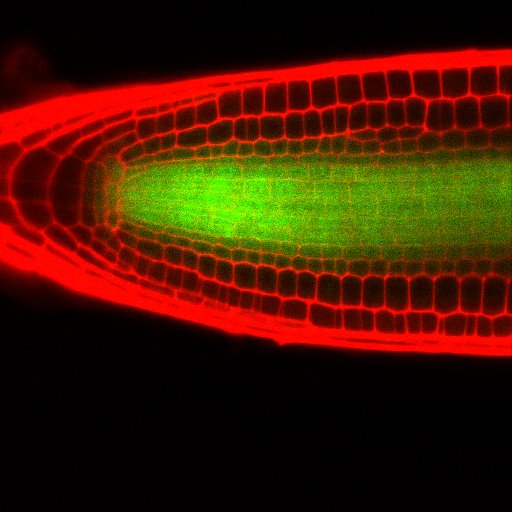

Supplement: Supplementary file 4 — Source Data Fig. 1 [file 44318_2024_71_MOESM4_ESM.zip › Fig 1/C/GFP quantification imagesImages/A10 NTF/Image 2.jpg]

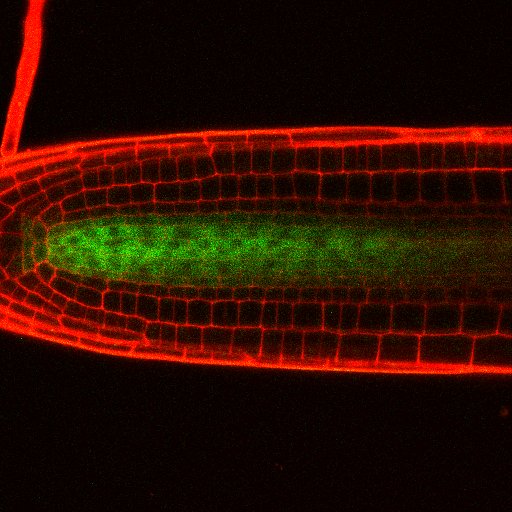

Supplement: Supplementary file 4 — Source Data Fig. 1 [file 44318_2024_71_MOESM4_ESM.zip › Fig 1/C/GFP quantification imagesImages/A10 NTF/Image 5.jpg]

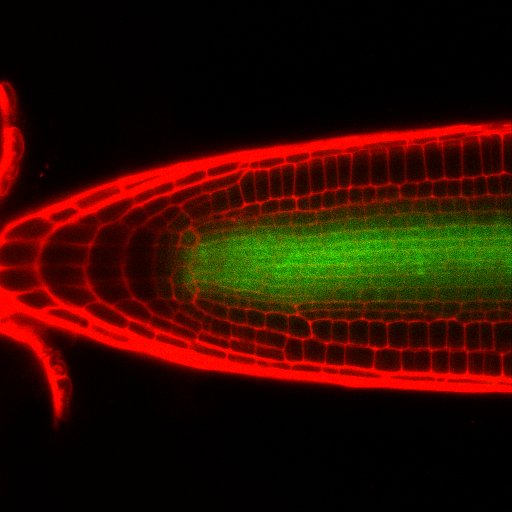

Supplement: Supplementary file 4 — Source Data Fig. 1 [file 44318_2024_71_MOESM4_ESM.zip › Fig 1/C/GFP quantification imagesImages/A10 NTF/Image 7.jpg]

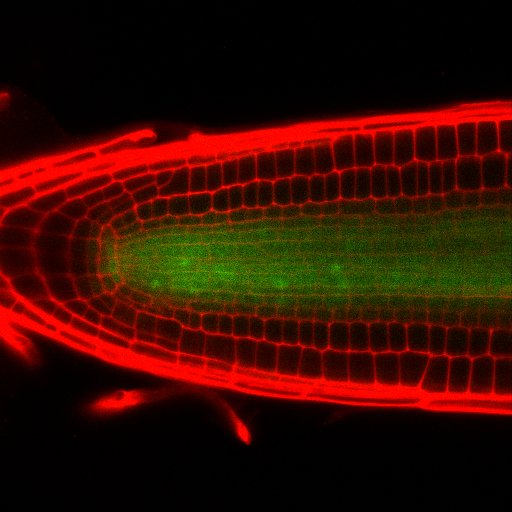

Supplement: Supplementary file 4 — Source Data Fig. 1 [file 44318_2024_71_MOESM4_ESM.zip › Fig 1/C/GFP quantification imagesImages/A10 NTF/Image 9.jpg]

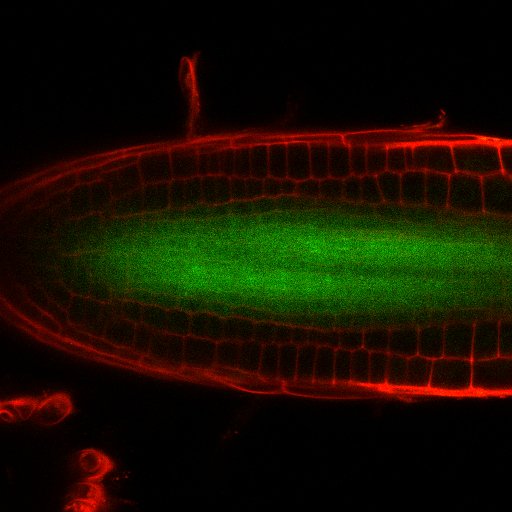

Supplement: Supplementary file 4 — Source Data Fig. 1 [file 44318_2024_71_MOESM4_ESM.zip › Fig 1/C/GFP quantification imagesImages/A10 NTF/Image 11.jpg]

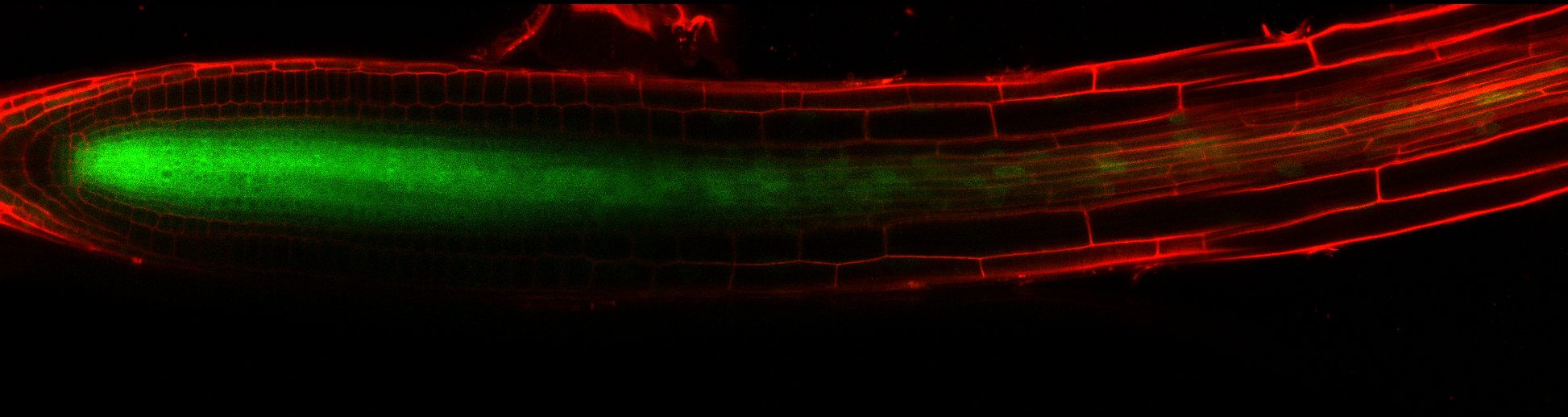

Supplement: Supplementary file 4 — Source Data Fig. 1 [file 44318_2024_71_MOESM4_ESM.zip › Fig 1/C/GFP quantification imagesImages/A10 NTF/Image 16.jpg]

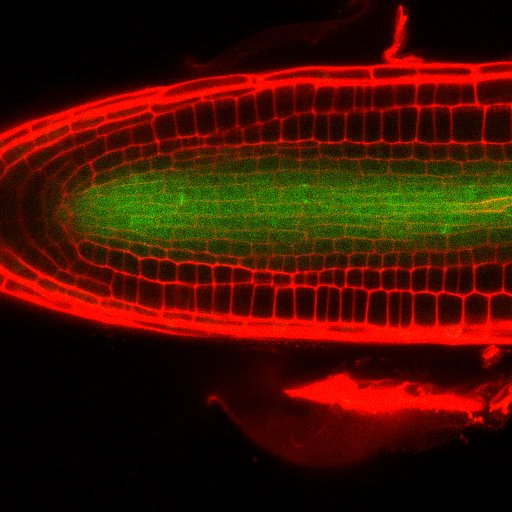

Supplement: Supplementary file 4 — Source Data Fig. 1 [file 44318_2024_71_MOESM4_ESM.zip › Fig 1/C/GFP quantification imagesImages/A10 NTF/Image 17.jpg]

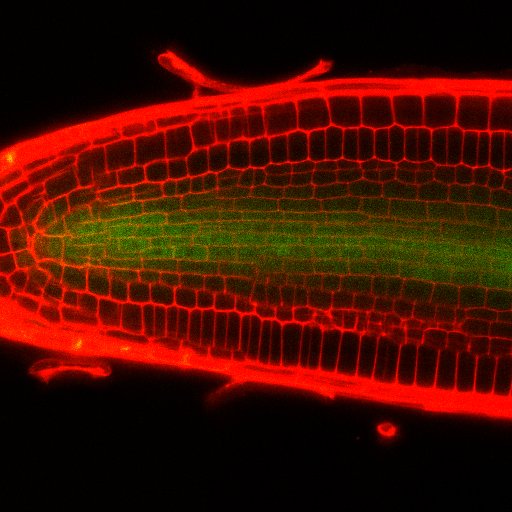

Supplement: Supplementary file 4 — Source Data Fig. 1 [file 44318_2024_71_MOESM4_ESM.zip › Fig 1/C/GFP quantification imagesImages/A10 NTF/Image 20.jpg]

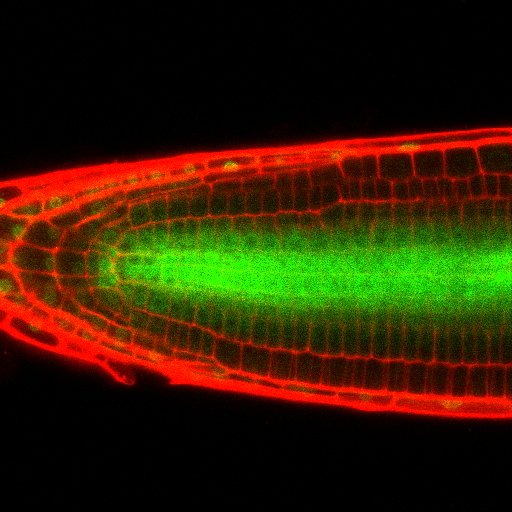

Supplement: Supplementary file 4 — Source Data Fig. 1 [file 44318_2024_71_MOESM4_ESM.zip › Fig 1/C/GFP quantification imagesImages/A10 NTF/Image 39-2.jpg]

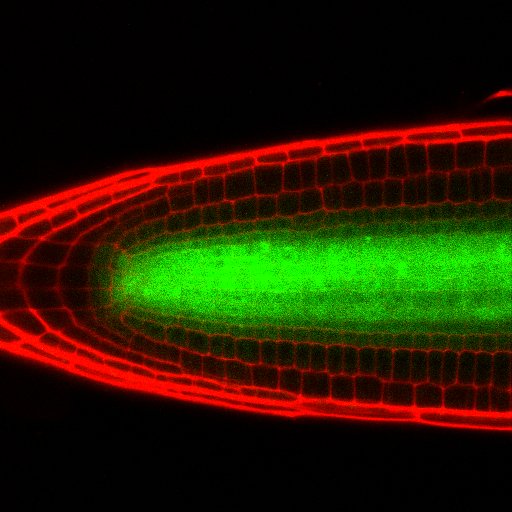

Supplement: Supplementary file 4 — Source Data Fig. 1 [file 44318_2024_71_MOESM4_ESM.zip › Fig 1/C/GFP quantification imagesImages/A10 NTF/Image 40.jpg]

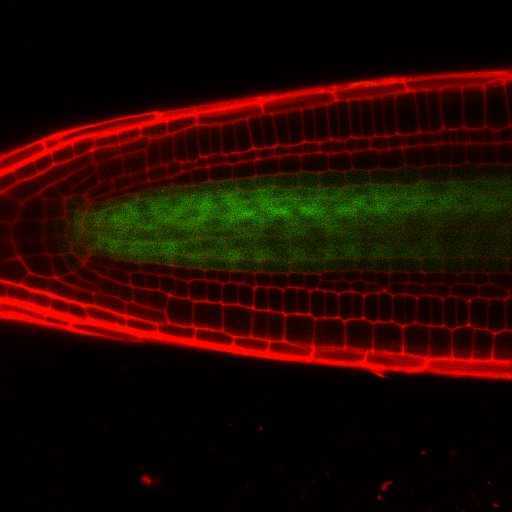

Supplement: Supplementary file 4 — Source Data Fig. 1 [file 44318_2024_71_MOESM4_ESM.zip › Fig 1/C/GFP quantification imagesImages/A10 NX/Image 1-2.jpg]

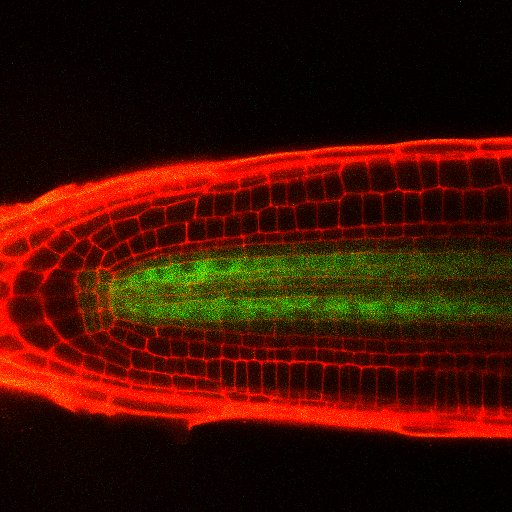

Supplement: Supplementary file 4 — Source Data Fig. 1 [file 44318_2024_71_MOESM4_ESM.zip › Fig 1/C/GFP quantification imagesImages/A10 NX/Image 2-2.jpg]

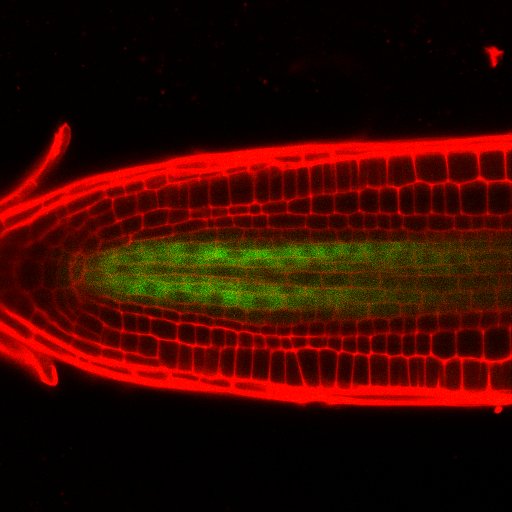

Supplement: Supplementary file 4 — Source Data Fig. 1 [file 44318_2024_71_MOESM4_ESM.zip › Fig 1/C/GFP quantification imagesImages/A10 NX/Image 2.jpg]

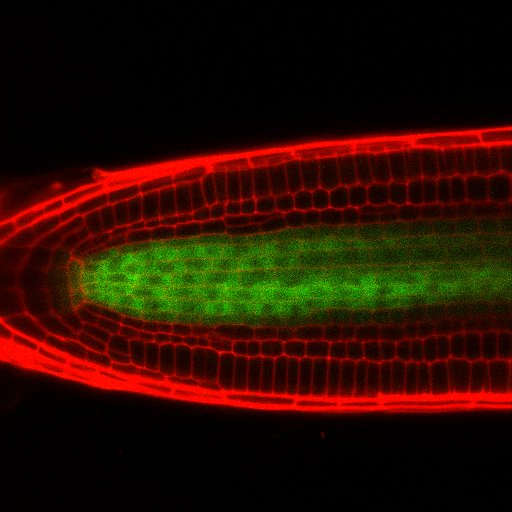

Supplement: Supplementary file 4 — Source Data Fig. 1 [file 44318_2024_71_MOESM4_ESM.zip › Fig 1/C/GFP quantification imagesImages/A10 NX/Image 3-2.jpg]

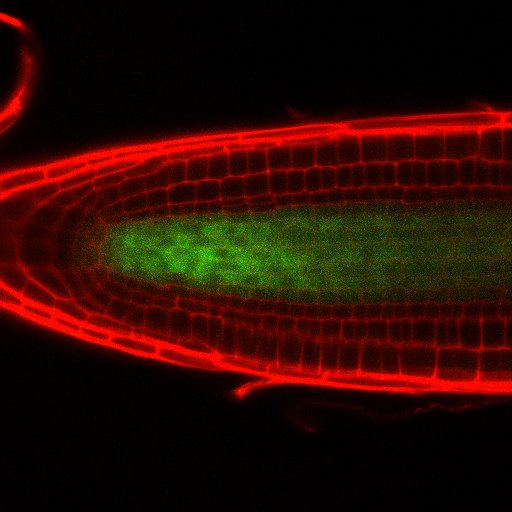

Supplement: Supplementary file 4 — Source Data Fig. 1 [file 44318_2024_71_MOESM4_ESM.zip › Fig 1/C/GFP quantification imagesImages/A10 NX/Image 4-2.jpg]

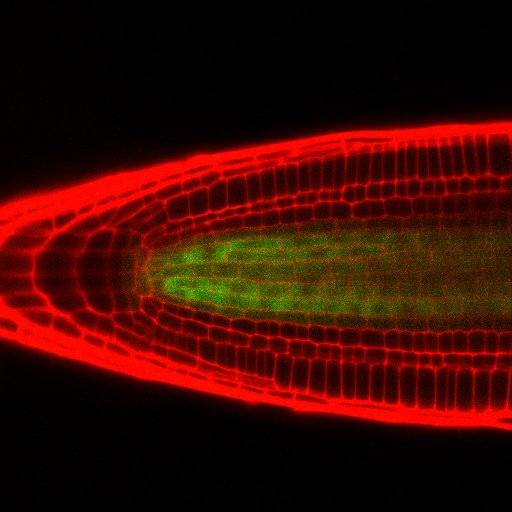

Supplement: Supplementary file 4 — Source Data Fig. 1 [file 44318_2024_71_MOESM4_ESM.zip › Fig 1/C/GFP quantification imagesImages/A10 NX/Image 5.jpg]

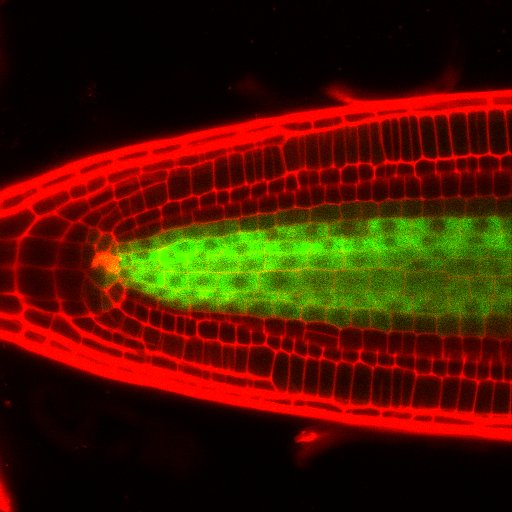

Supplement: Supplementary file 4 — Source Data Fig. 1 [file 44318_2024_71_MOESM4_ESM.zip › Fig 1/C/GFP quantification imagesImages/A10 NX/Image 6-2.jpg]

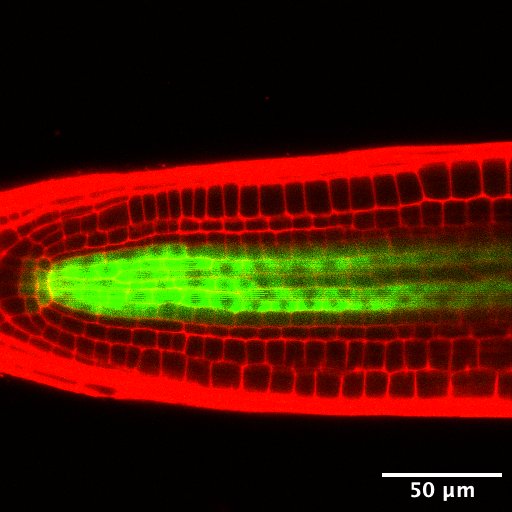

Supplement: Supplementary file 4 — Source Data Fig. 1 [file 44318_2024_71_MOESM4_ESM.zip › Fig 1/C/GFP quantification imagesImages/A10 NX/Image 29.jpg]

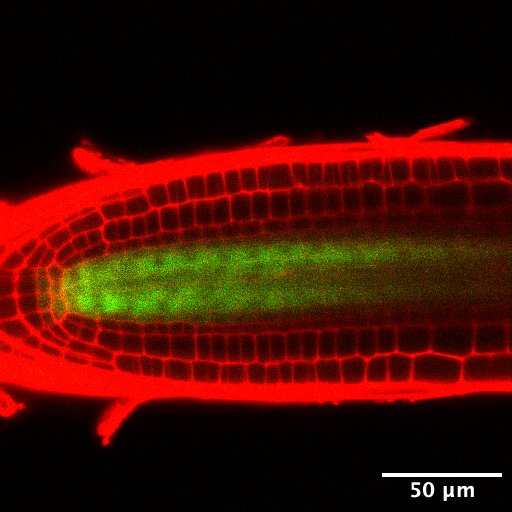

Supplement: Supplementary file 4 — Source Data Fig. 1 [file 44318_2024_71_MOESM4_ESM.zip › Fig 1/C/GFP quantification imagesImages/A10 NX/Image 32.jpg]

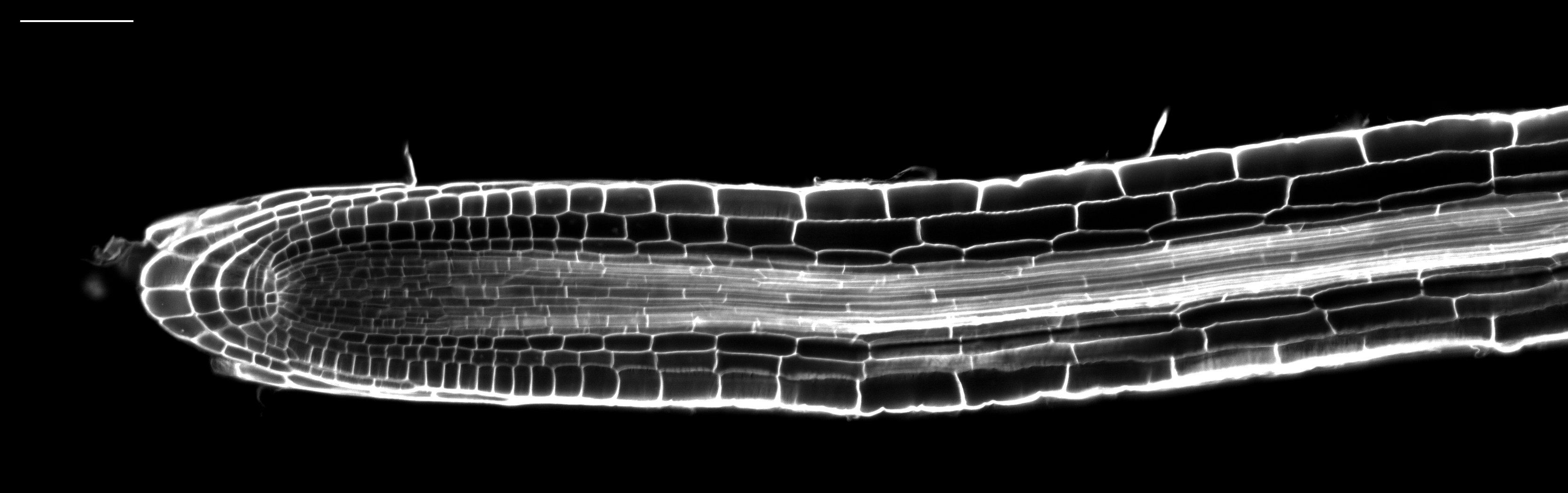

Supplement: Supplementary file 5 — Source Data Fig. 2 [file 44318_2024_71_MOESM5_ESM.zip › Fig 2/A/A10 OX.jpg]

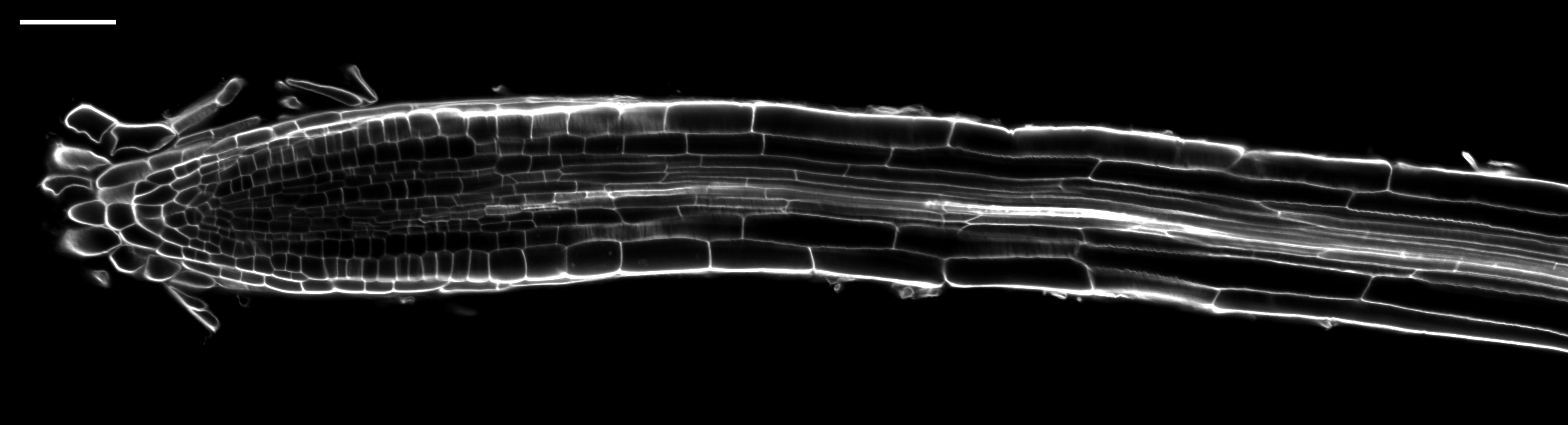

Supplement: Supplementary file 5 — Source Data Fig. 2 [file 44318_2024_71_MOESM5_ESM.zip › Fig 2/A/shr-2.jpg]

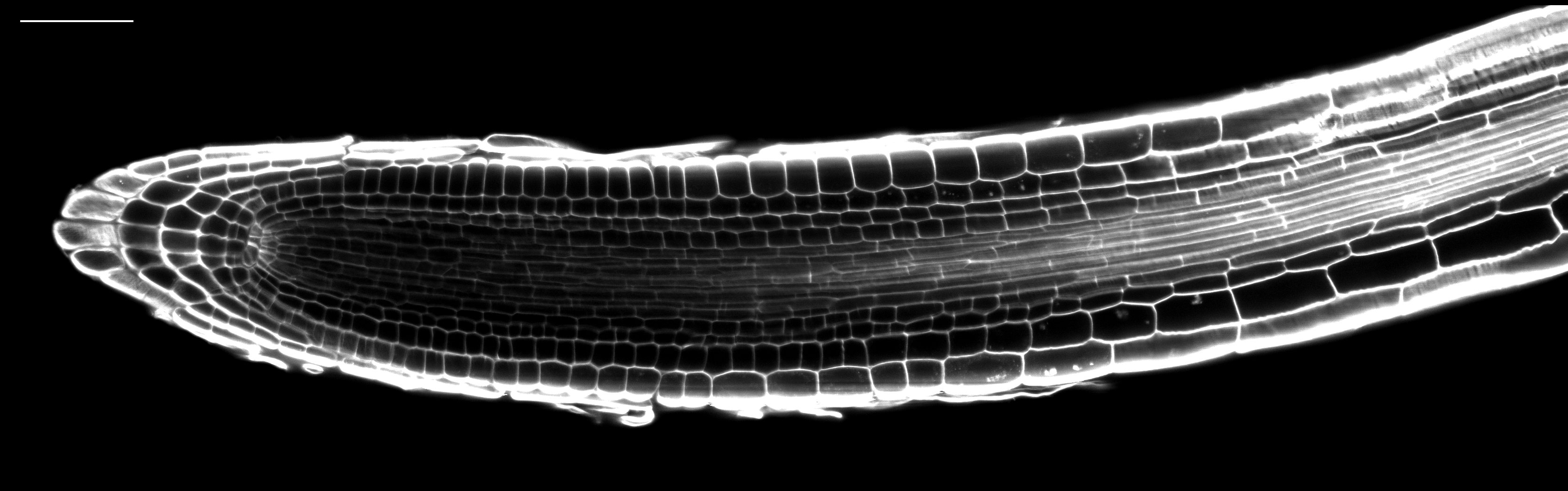

Supplement: Supplementary file 5 — Source Data Fig. 2 [file 44318_2024_71_MOESM5_ESM.zip › Fig 2/A/ago10-1.jpg]

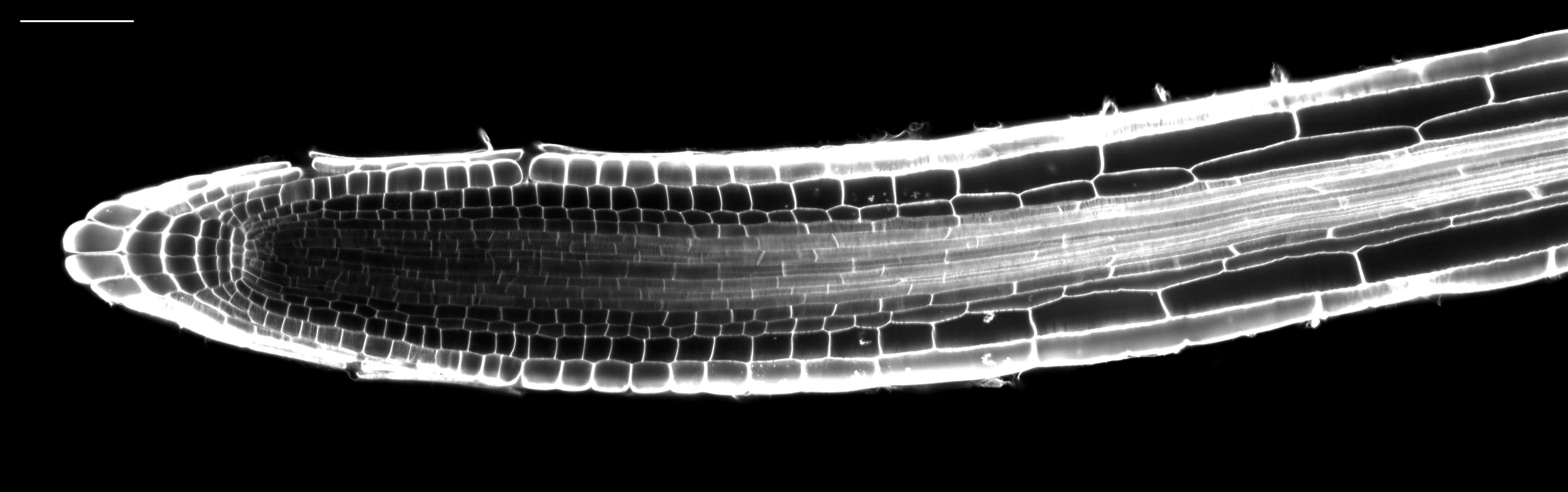

Supplement: Supplementary file 5 — Source Data Fig. 2 [file 44318_2024_71_MOESM5_ESM.zip › Fig 2/A/WT.jpg]

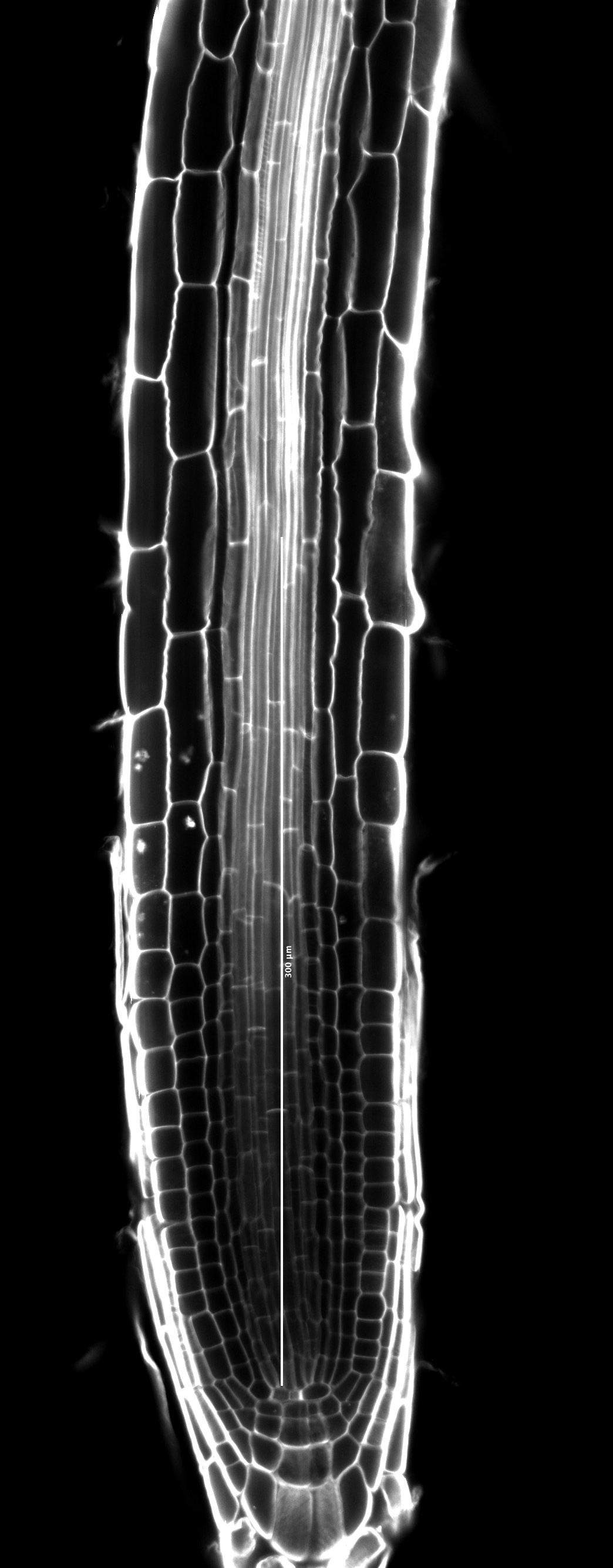

Supplement: Supplementary file 5 — Source Data Fig. 2 [file 44318_2024_71_MOESM5_ESM.zip › Fig 2/C/A10OX.jpg]

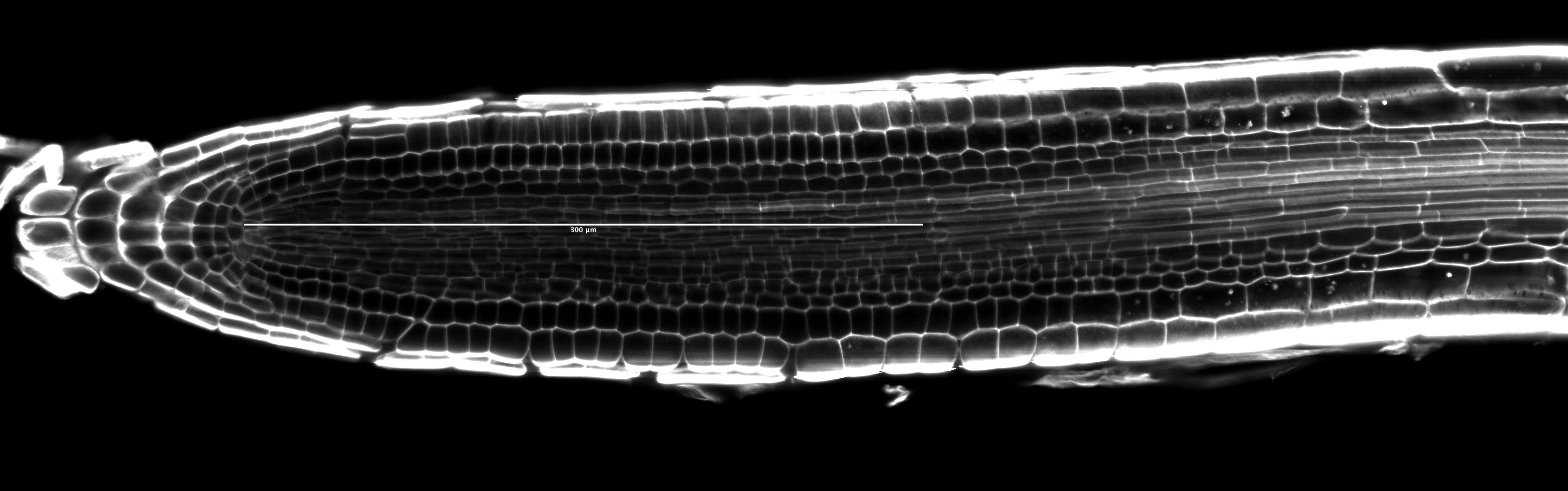

Supplement: Supplementary file 5 — Source Data Fig. 2 [file 44318_2024_71_MOESM5_ESM.zip › Fig 2/C/ago10-1.jpg]

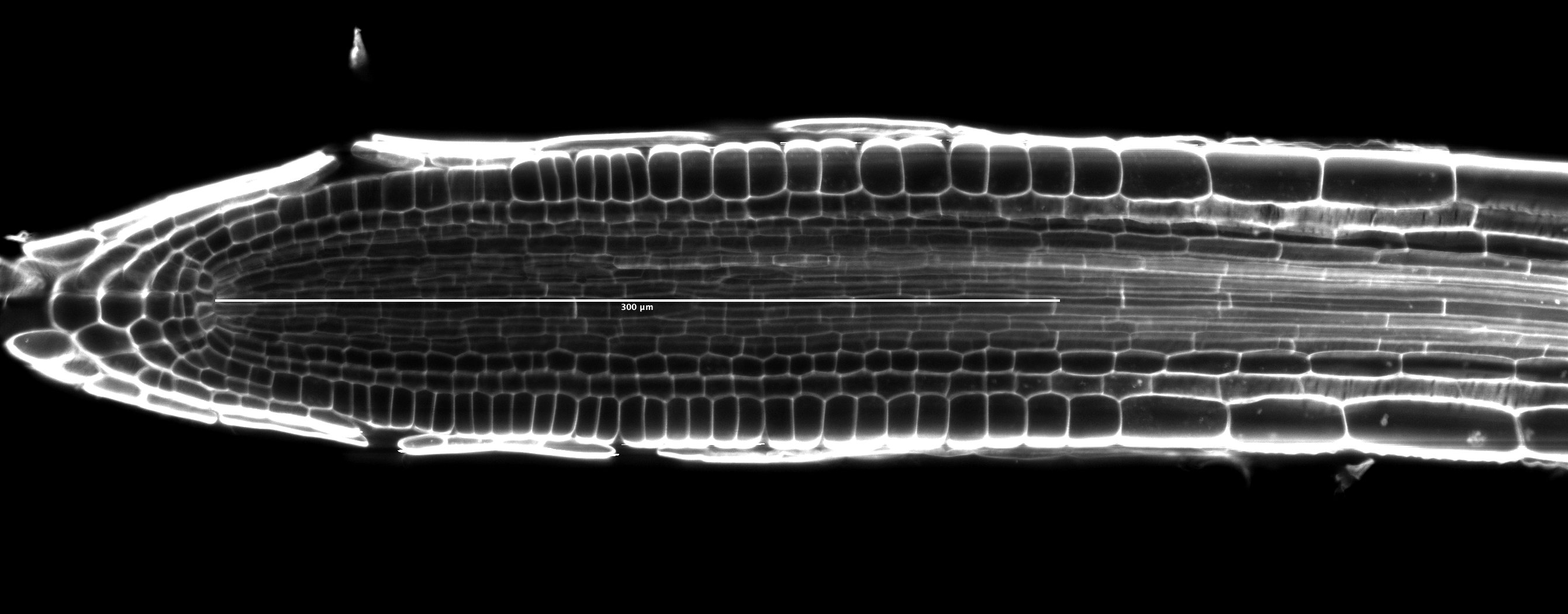

Supplement: Supplementary file 5 — Source Data Fig. 2 [file 44318_2024_71_MOESM5_ESM.zip › Fig 2/C/WT.jpg]

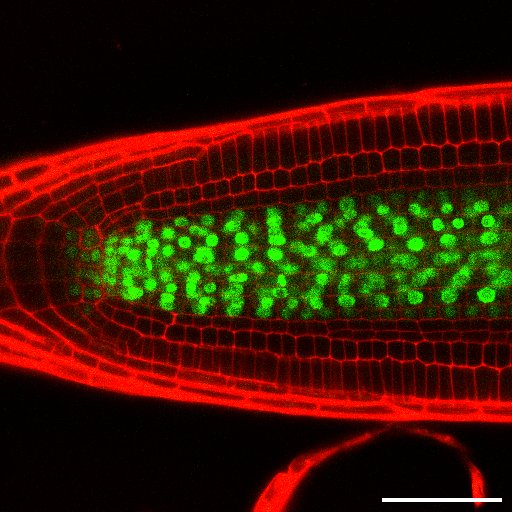

Supplement: Supplementary file 5 — Source Data Fig. 2 [file 44318_2024_71_MOESM5_ESM.zip › Fig 2/H/A10 H2B GFP.jpg]

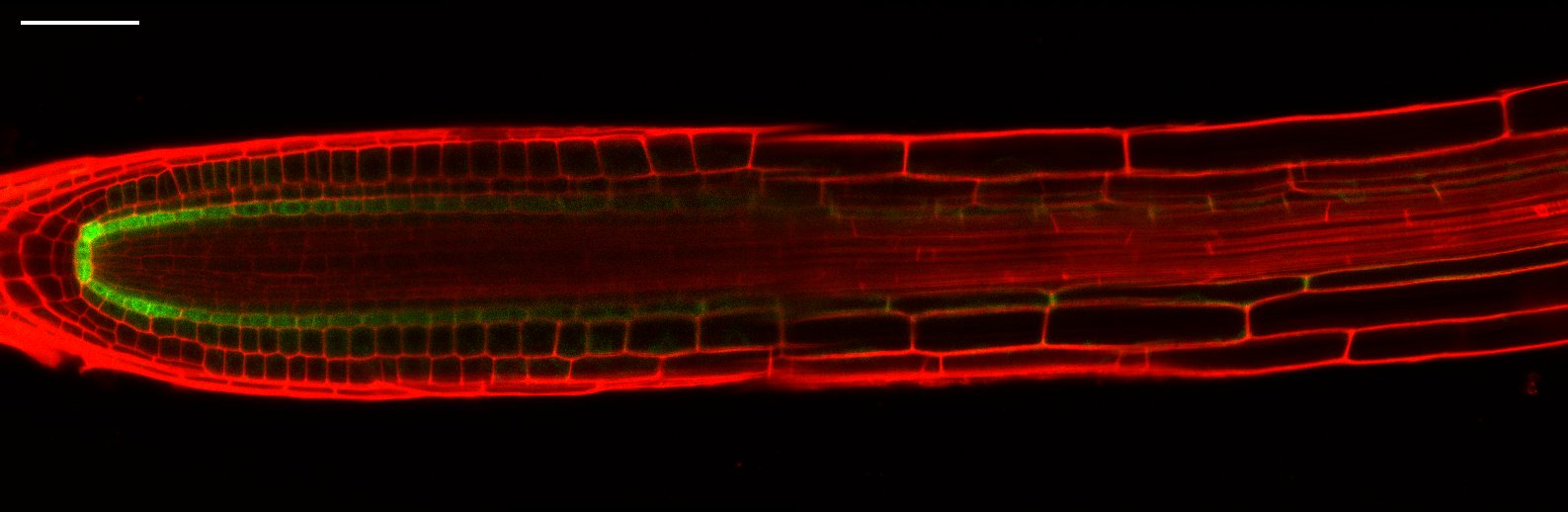

Supplement: Supplementary file 5 — Source Data Fig. 2 [file 44318_2024_71_MOESM5_ESM.zip › Fig 2/H/pmiR166bGFP.jpg]

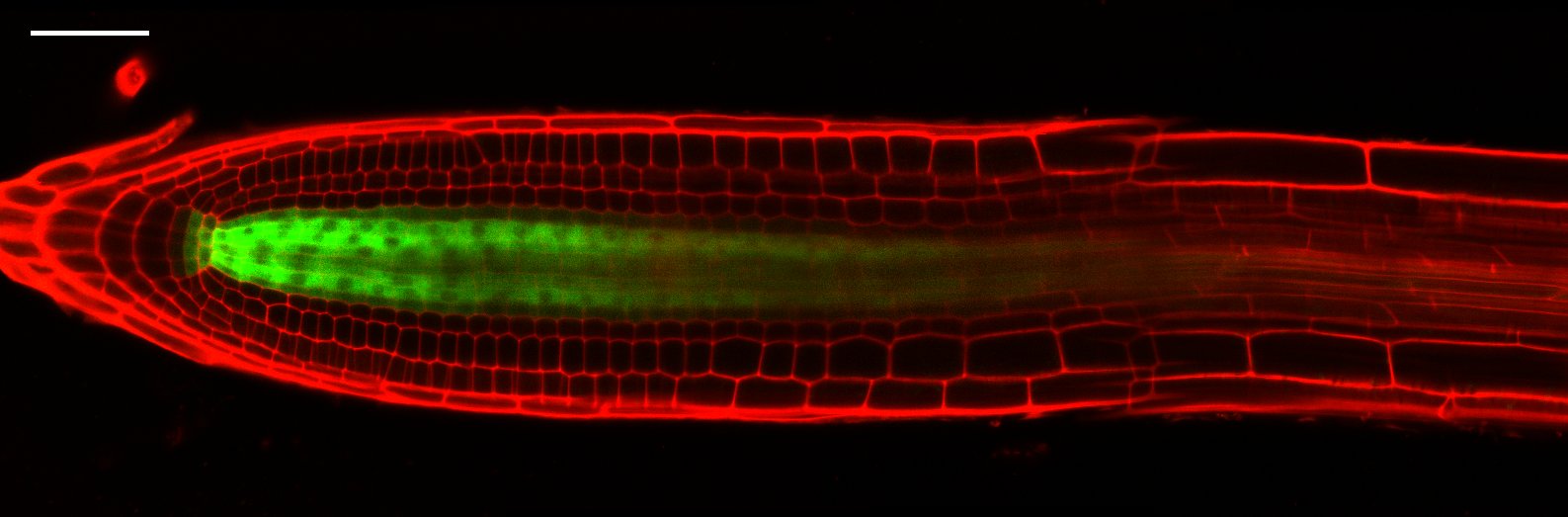

Supplement: Supplementary file 5 — Source Data Fig. 2 [file 44318_2024_71_MOESM5_ESM.zip › Fig 2/H/A10 NX.jpg]

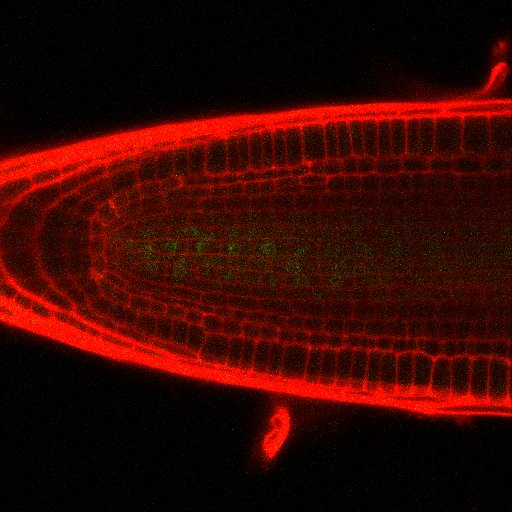

Supplement: Supplementary file 5 — Source Data Fig. 2 [file 44318_2024_71_MOESM5_ESM.zip › Fig 2/H/PHB GFP in ago10-1.jpg]

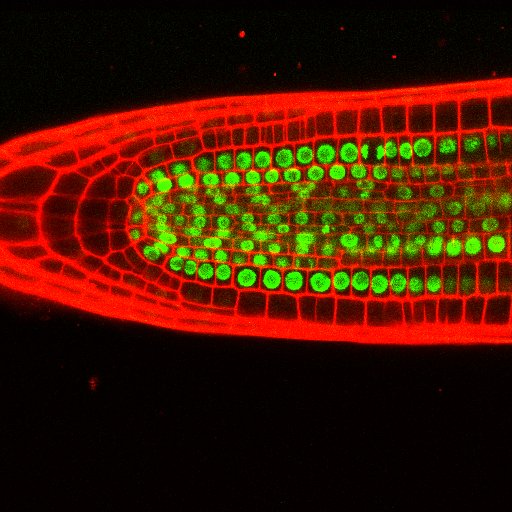

Supplement: Supplementary file 5 — Source Data Fig. 2 [file 44318_2024_71_MOESM5_ESM.zip › Fig 2/H/PHB H2B GFP.jpg]

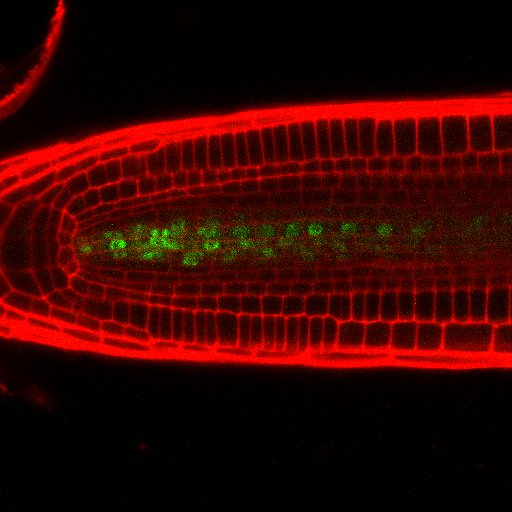

Supplement: Supplementary file 5 — Source Data Fig. 2 [file 44318_2024_71_MOESM5_ESM.zip › Fig 2/H/PHB PHB GFP.jpg]

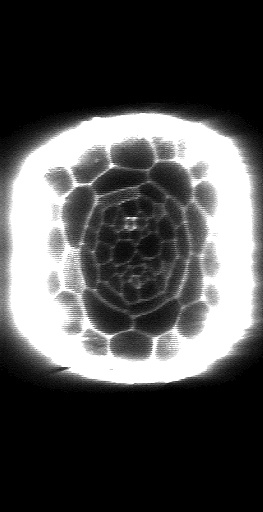

Supplement: Supplementary file 5 — Source Data Fig. 2 [file 44318_2024_71_MOESM5_ESM.zip › Fig 2/E/A10 OX/120uM above QC.tif]

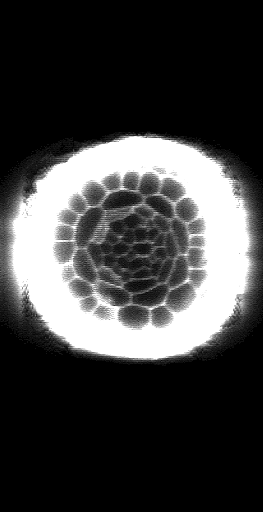

Supplement: Supplementary file 5 — Source Data Fig. 2 [file 44318_2024_71_MOESM5_ESM.zip › Fig 2/E/A10 OX/20uM above QC.tif]

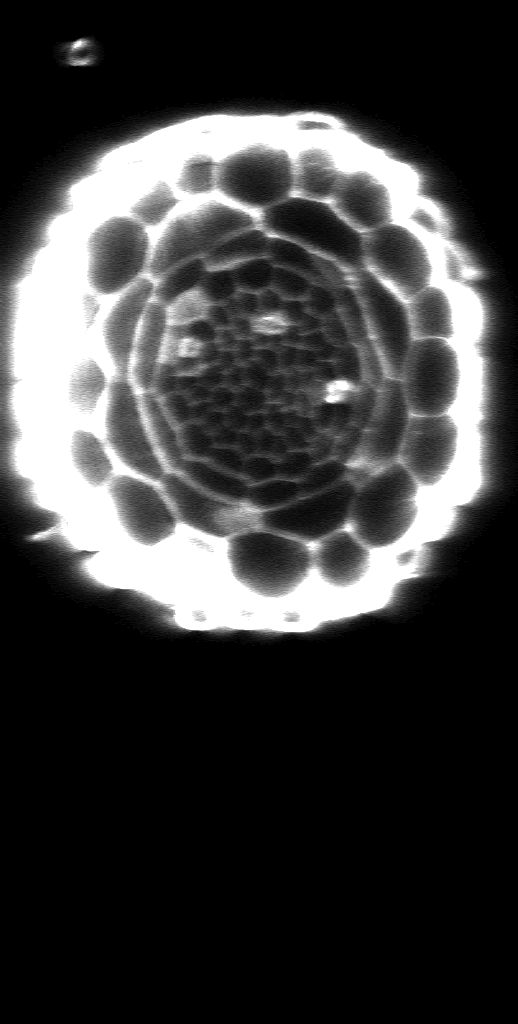

Supplement: Supplementary file 5 — Source Data Fig. 2 [file 44318_2024_71_MOESM5_ESM.zip › Fig 2/E/ago10-1/120uM above QC.tif]

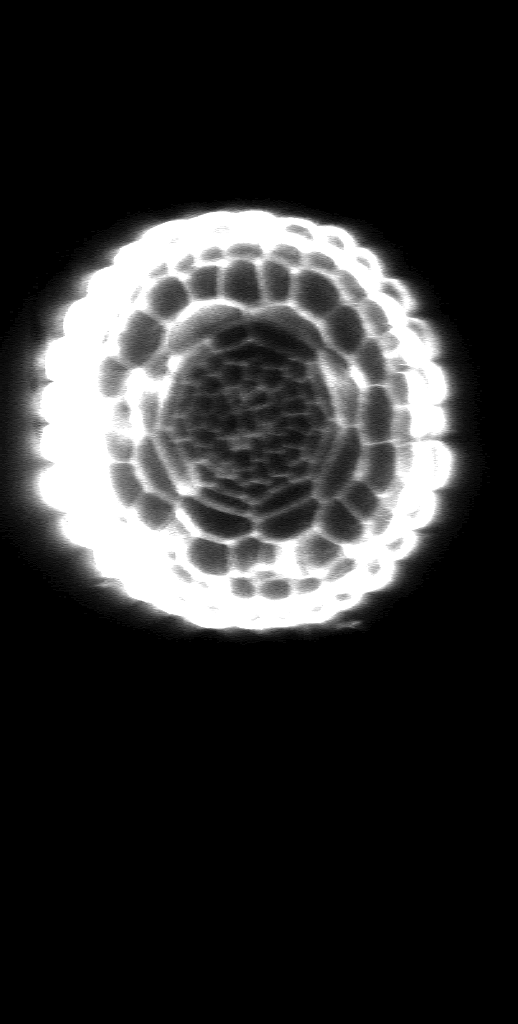

Supplement: Supplementary file 5 — Source Data Fig. 2 [file 44318_2024_71_MOESM5_ESM.zip › Fig 2/E/ago10-1/20uM above QC.tif]

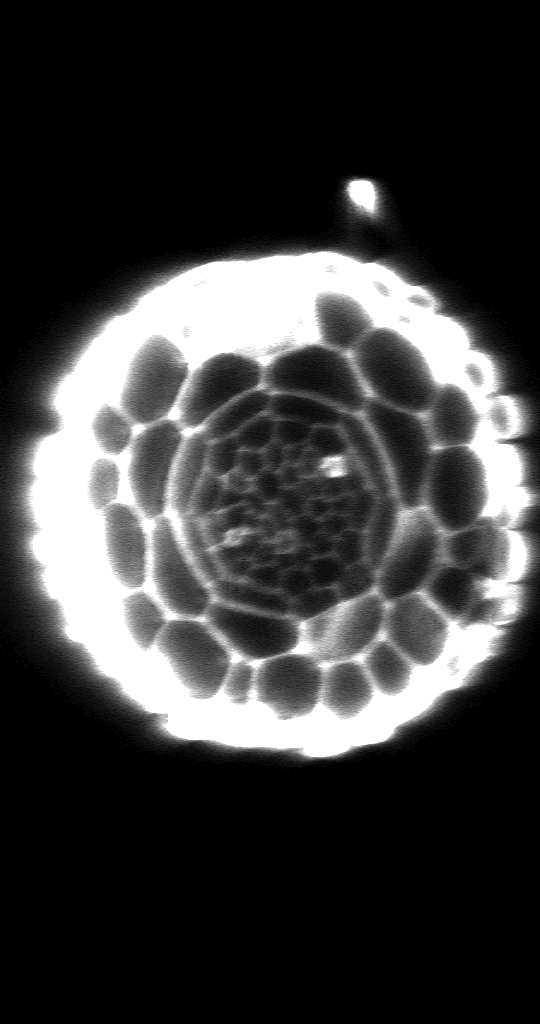

Supplement: Supplementary file 5 — Source Data Fig. 2 [file 44318_2024_71_MOESM5_ESM.zip › Fig 2/E/WT/120uM above QC.tif]

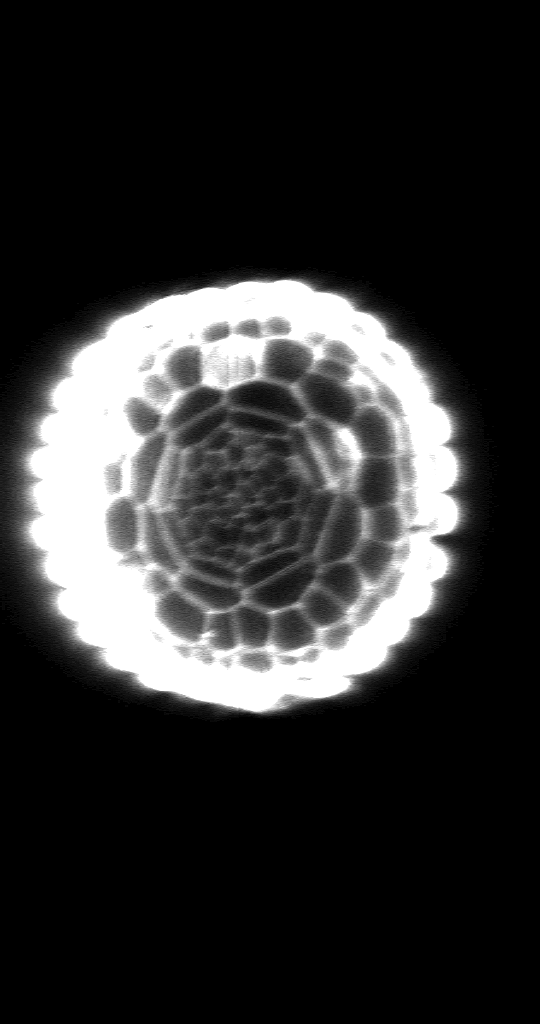

Supplement: Supplementary file 5 — Source Data Fig. 2 [file 44318_2024_71_MOESM5_ESM.zip › Fig 2/E/WT/20uM above QC.tif]

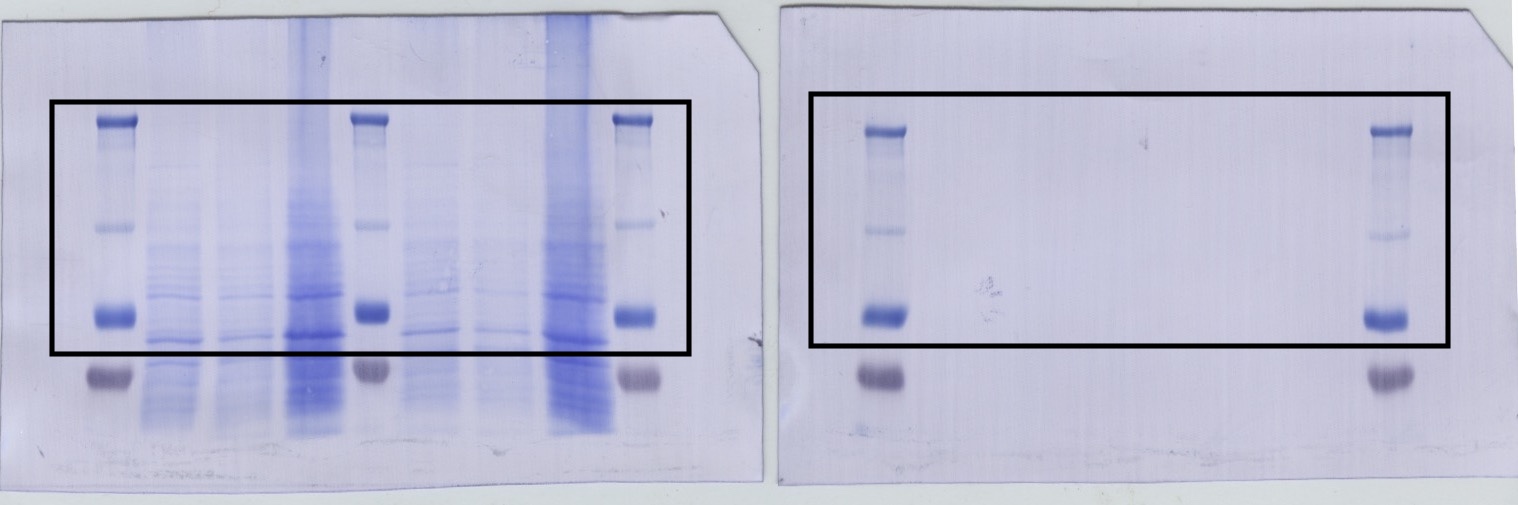

Supplement: Supplementary file 6 — Source Data Fig. 3 [file 44318_2024_71_MOESM6_ESM.zip › Fig 3/A/Figure_3_coomassie_WB.jpeg]

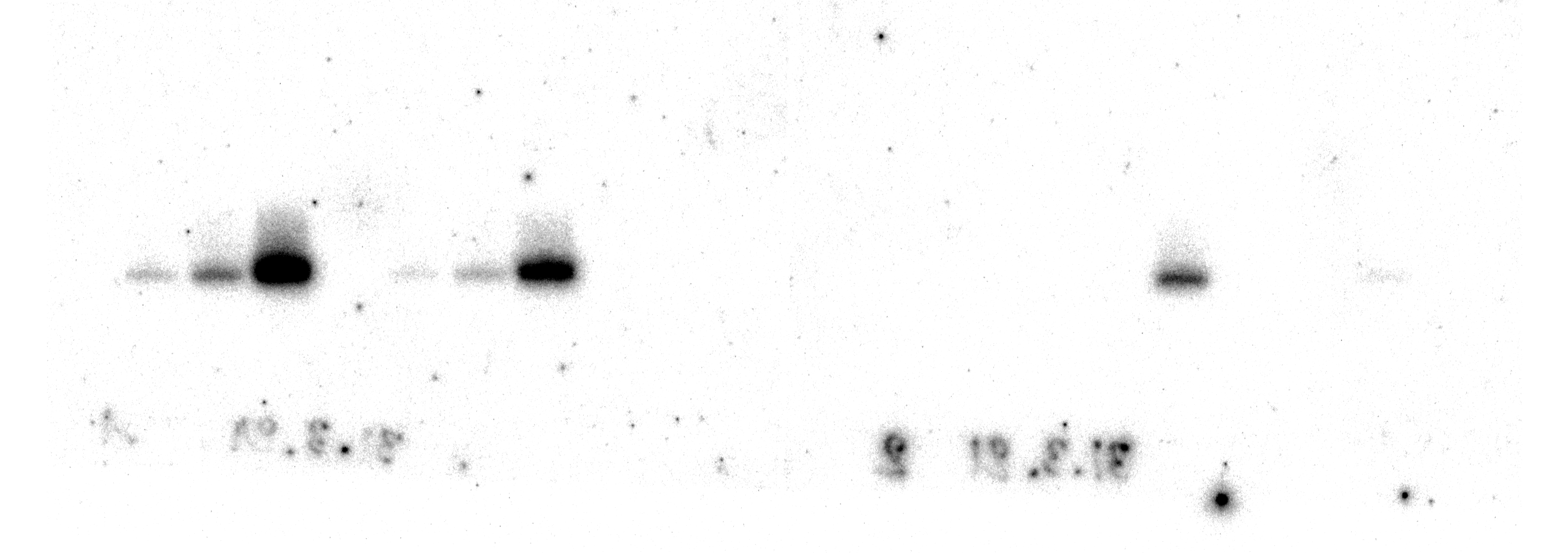

Supplement: Supplementary file 6 — Source Data Fig. 3 [file 44318_2024_71_MOESM6_ESM.zip › Fig 3/A/Figure_3A_NB_158.tif]

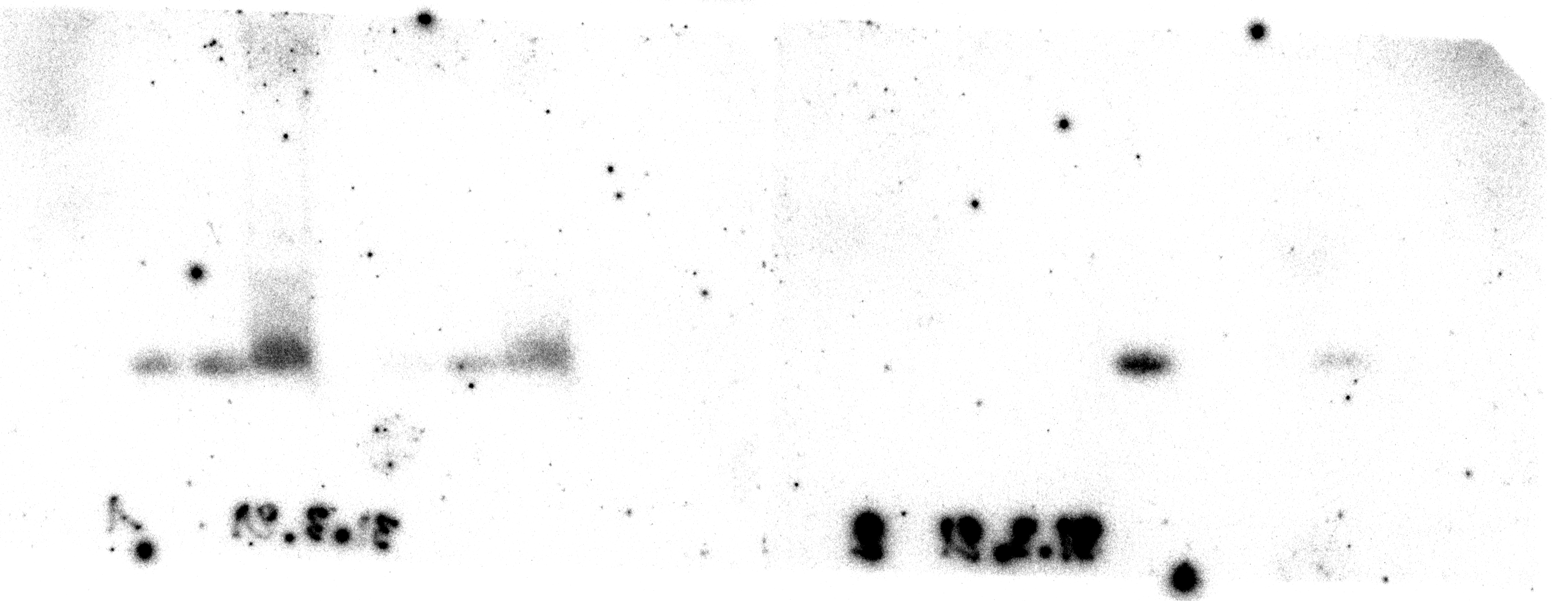

Supplement: Supplementary file 6 — Source Data Fig. 3 [file 44318_2024_71_MOESM6_ESM.zip › Fig 3/A/Figure_3A_NB_160.tif]

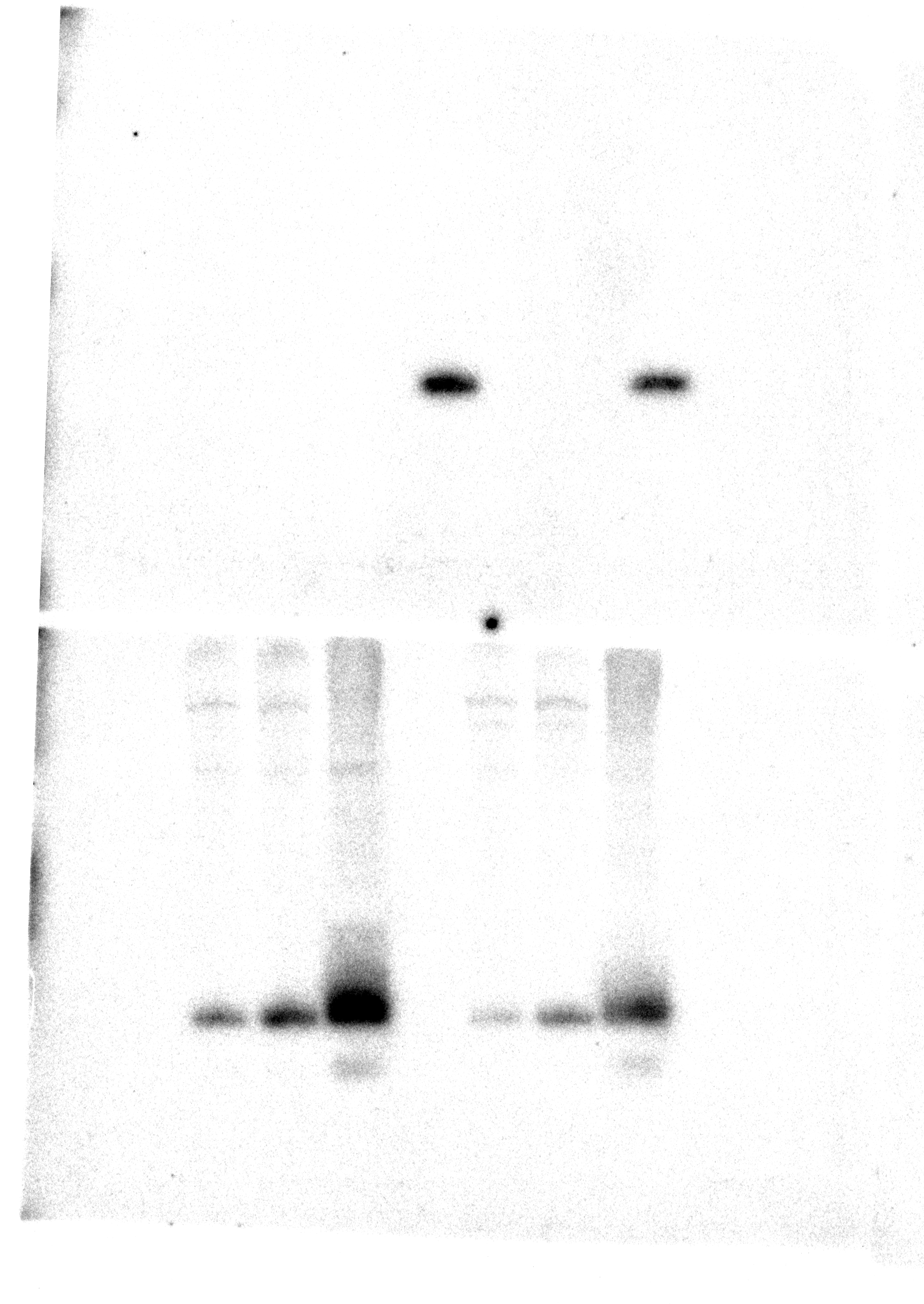

Supplement: Supplementary file 6 — Source Data Fig. 3 [file 44318_2024_71_MOESM6_ESM.zip › Fig 3/A/Figure_3A_NB_165_166.tif]

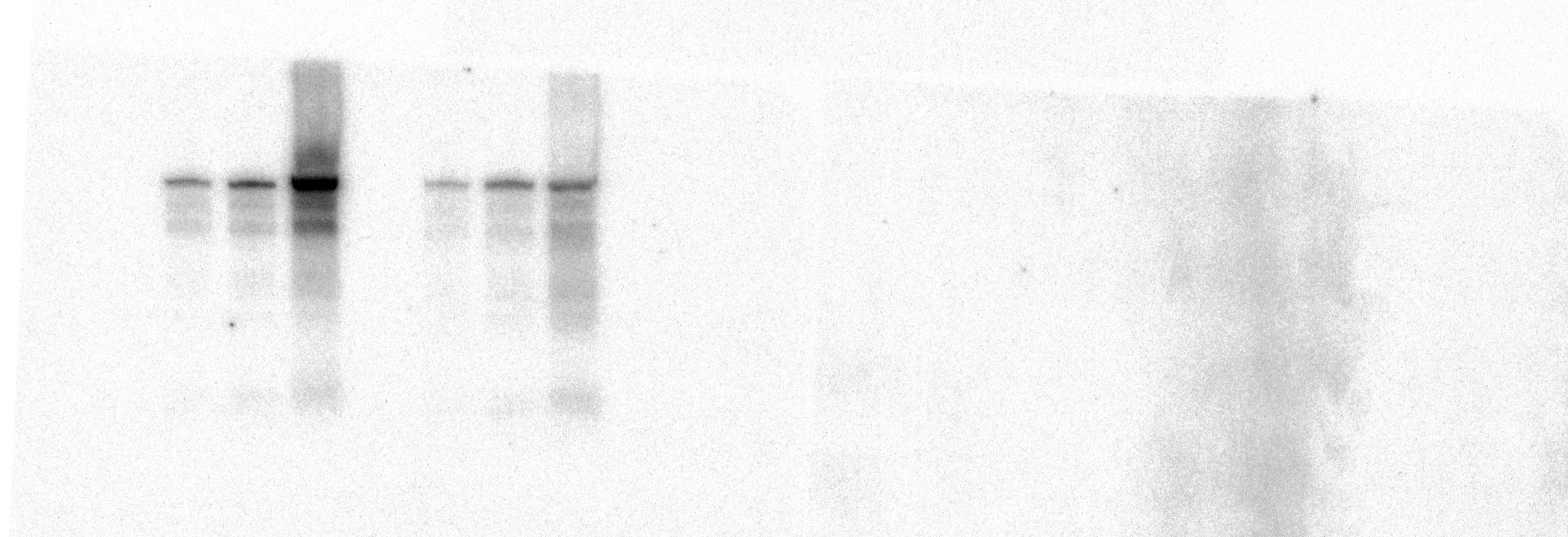

Supplement: Supplementary file 6 — Source Data Fig. 3 [file 44318_2024_71_MOESM6_ESM.zip › Fig 3/A/Figure_3A_NB_U6.tif]

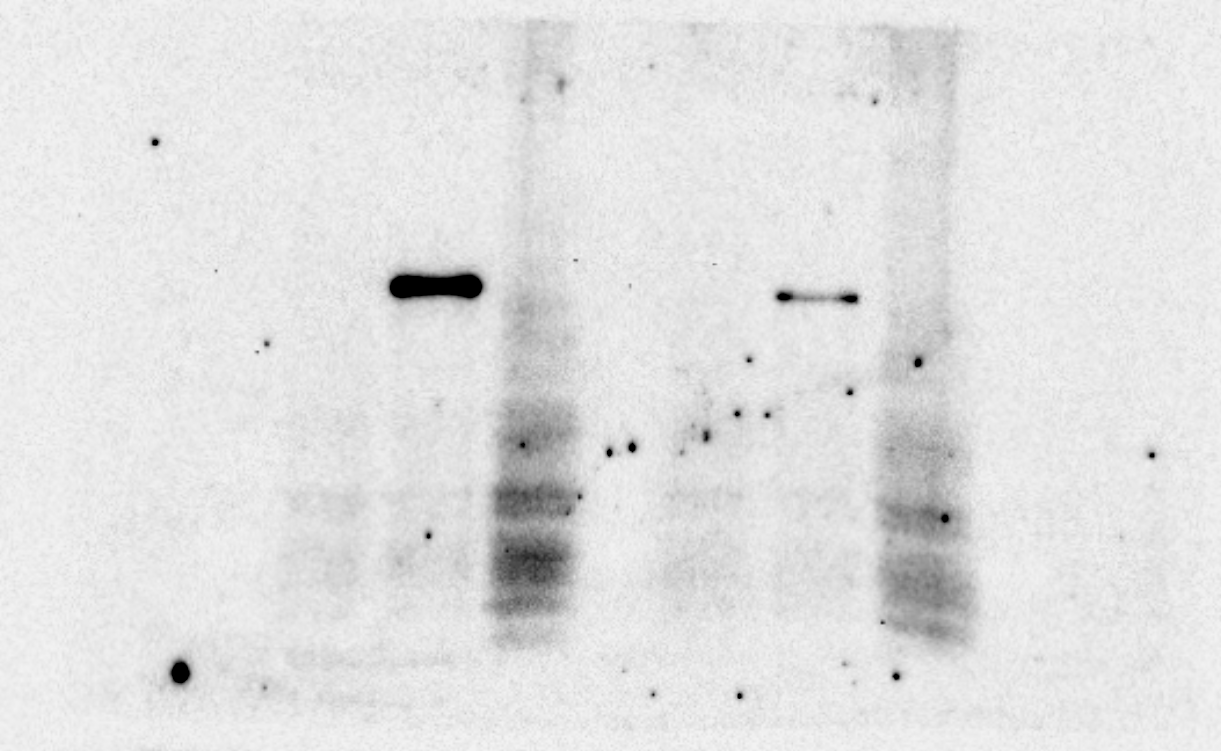

Supplement: Supplementary file 6 — Source Data Fig. 3 [file 44318_2024_71_MOESM6_ESM.zip › Fig 3/A/Figure_3A_WB_GFP_input.tif]

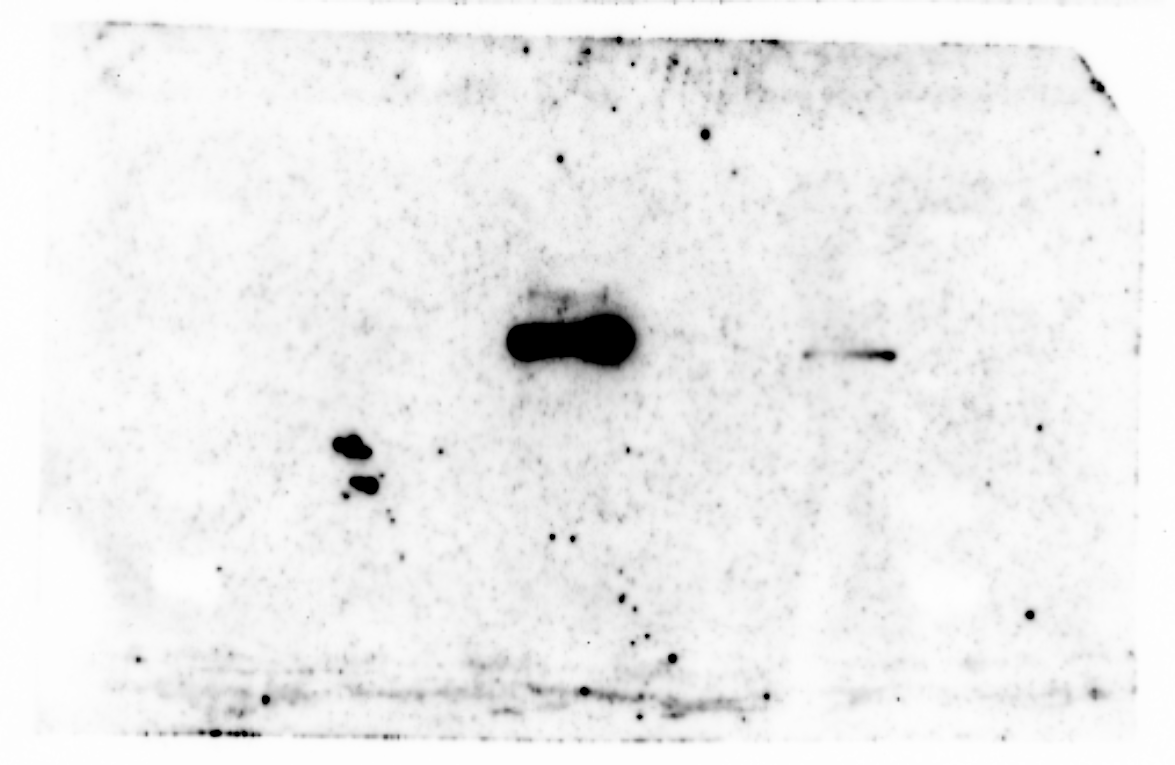

Supplement: Supplementary file 6 — Source Data Fig. 3 [file 44318_2024_71_MOESM6_ESM.zip › Fig 3/A/Figure_3A_WB_GFP_IP.tif]

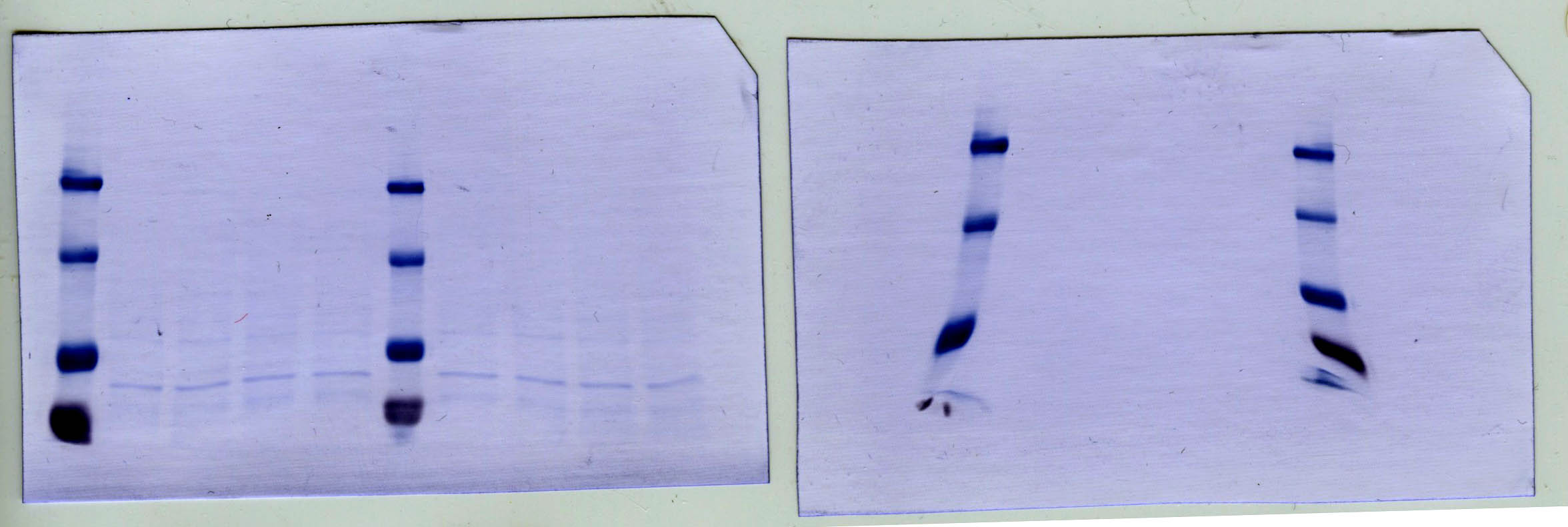

Supplement: Supplementary file 6 — Source Data Fig. 3 [file 44318_2024_71_MOESM6_ESM.zip › Fig 3/B/Figure_3B_coomassie_WB_IP.jpg]

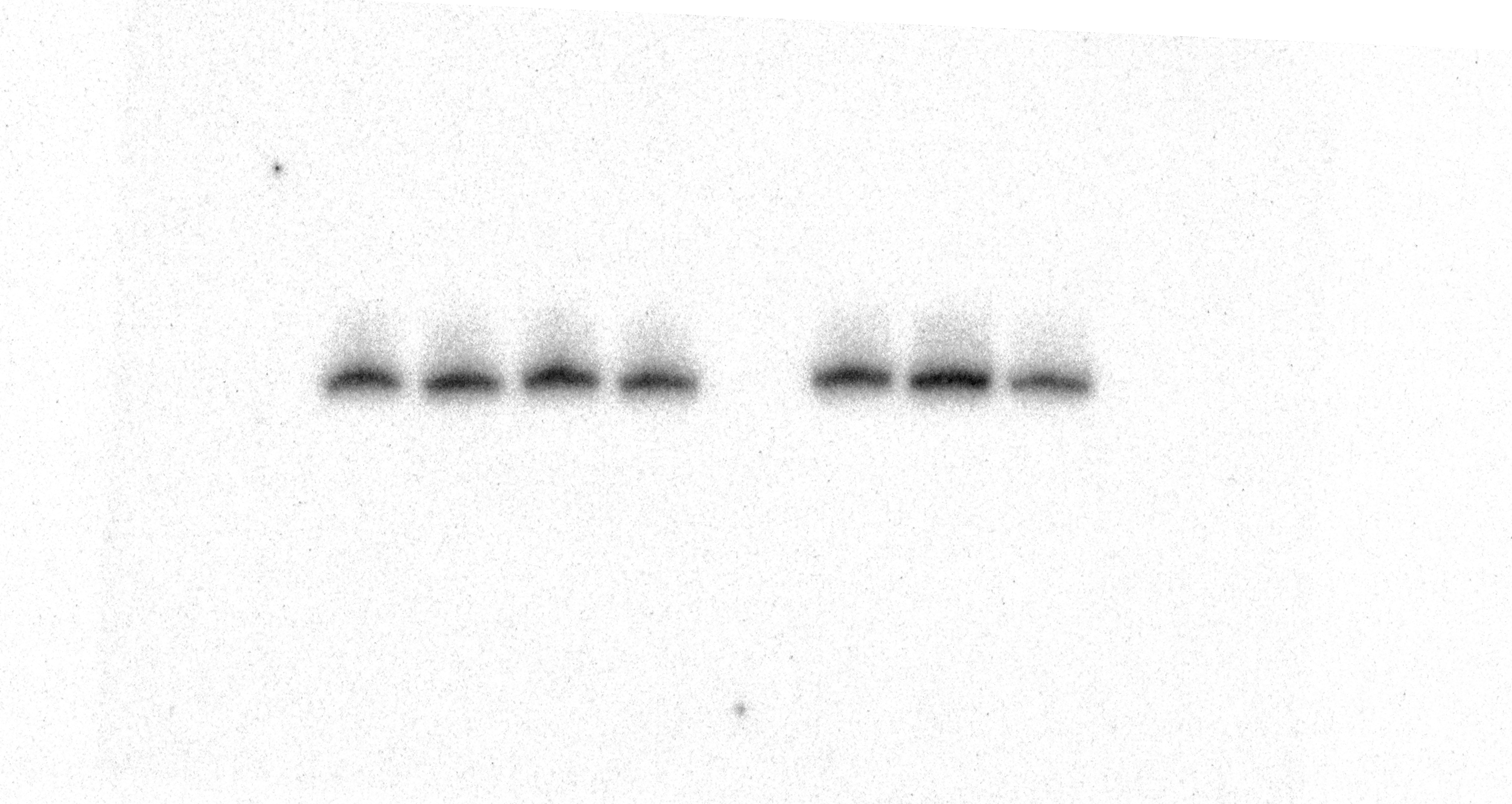

Supplement: Supplementary file 6 — Source Data Fig. 3 [file 44318_2024_71_MOESM6_ESM.zip › Fig 3/B/Figure_3B_NB_158.tif]

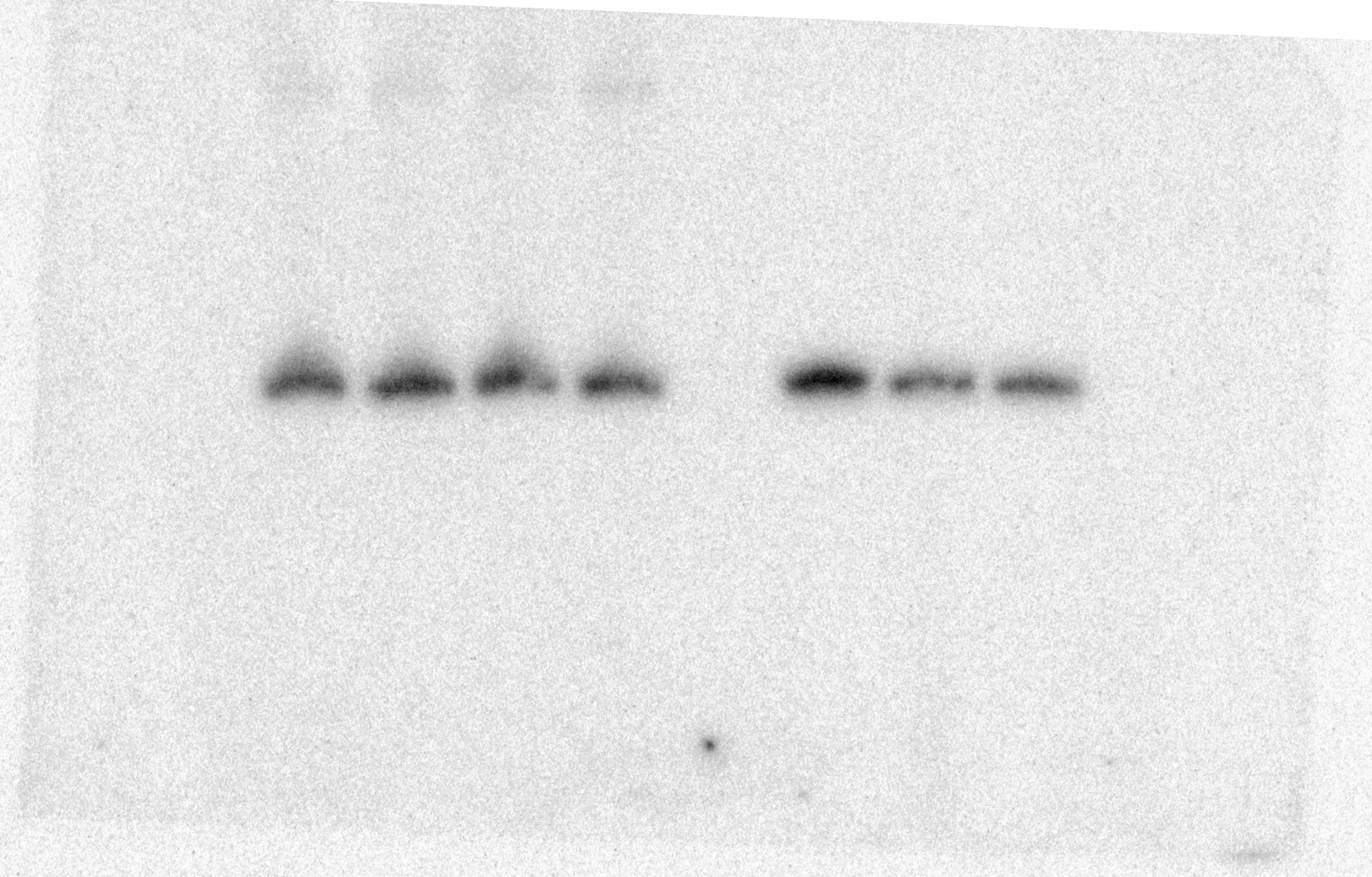

Supplement: Supplementary file 6 — Source Data Fig. 3 [file 44318_2024_71_MOESM6_ESM.zip › Fig 3/B/Figure_3B_NB_165_166.tif]

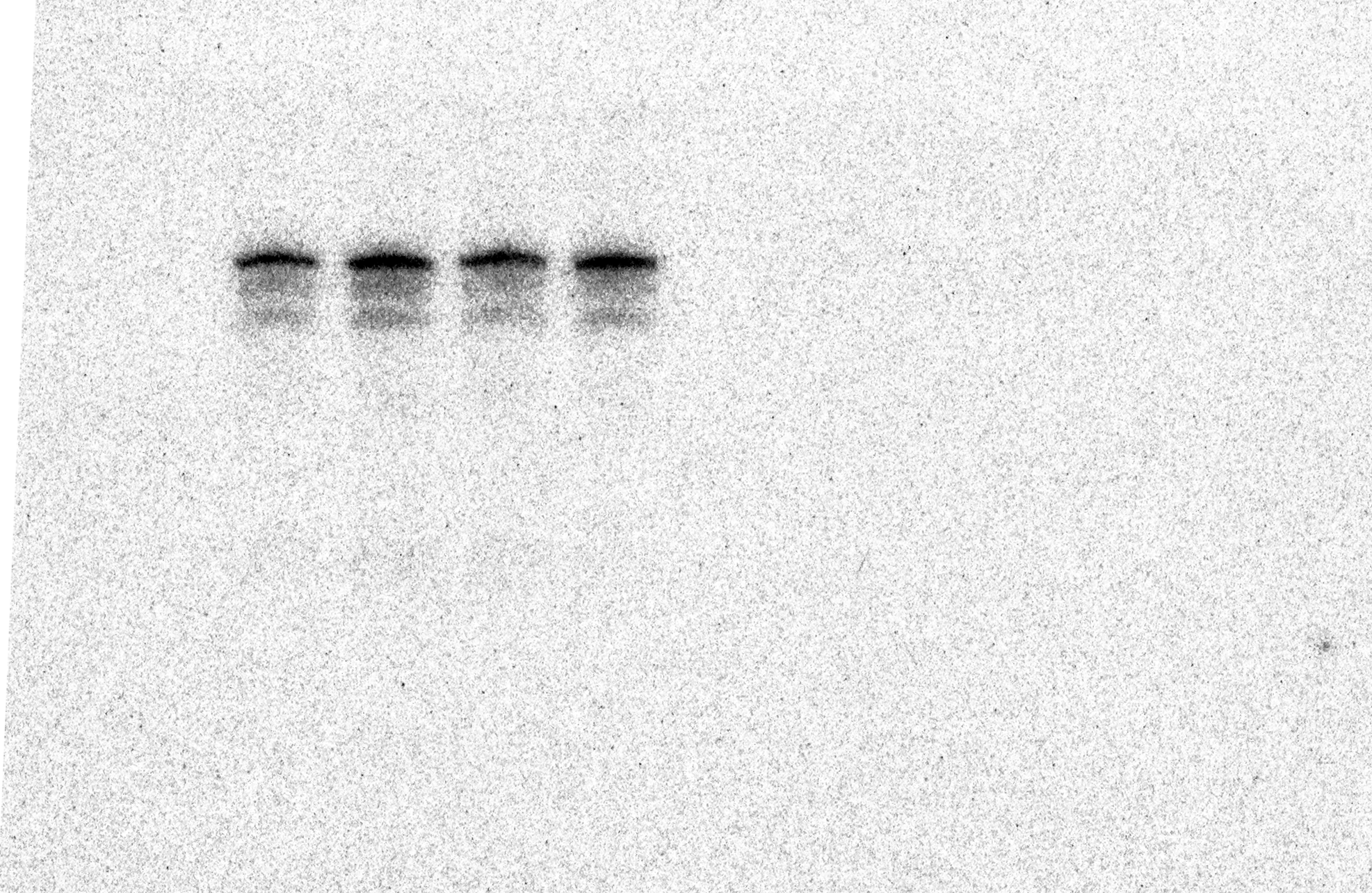

Supplement: Supplementary file 6 — Source Data Fig. 3 [file 44318_2024_71_MOESM6_ESM.zip › Fig 3/B/Figure_3B_NB_U6.tif]

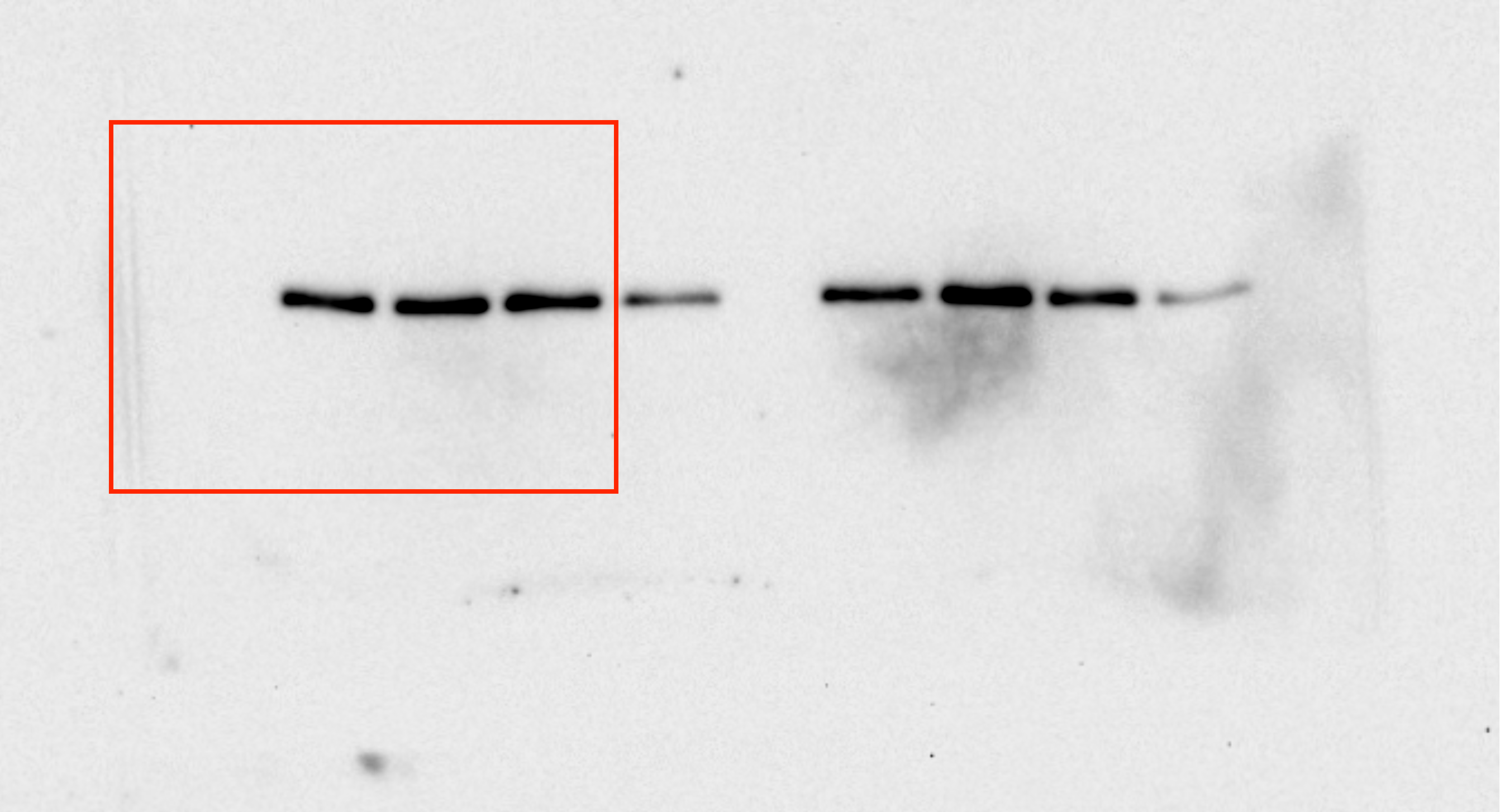

Supplement: Supplementary file 6 — Source Data Fig. 3 [file 44318_2024_71_MOESM6_ESM.zip › Fig 3/B/Figure_3B_WB_AGO1_Input.tif]

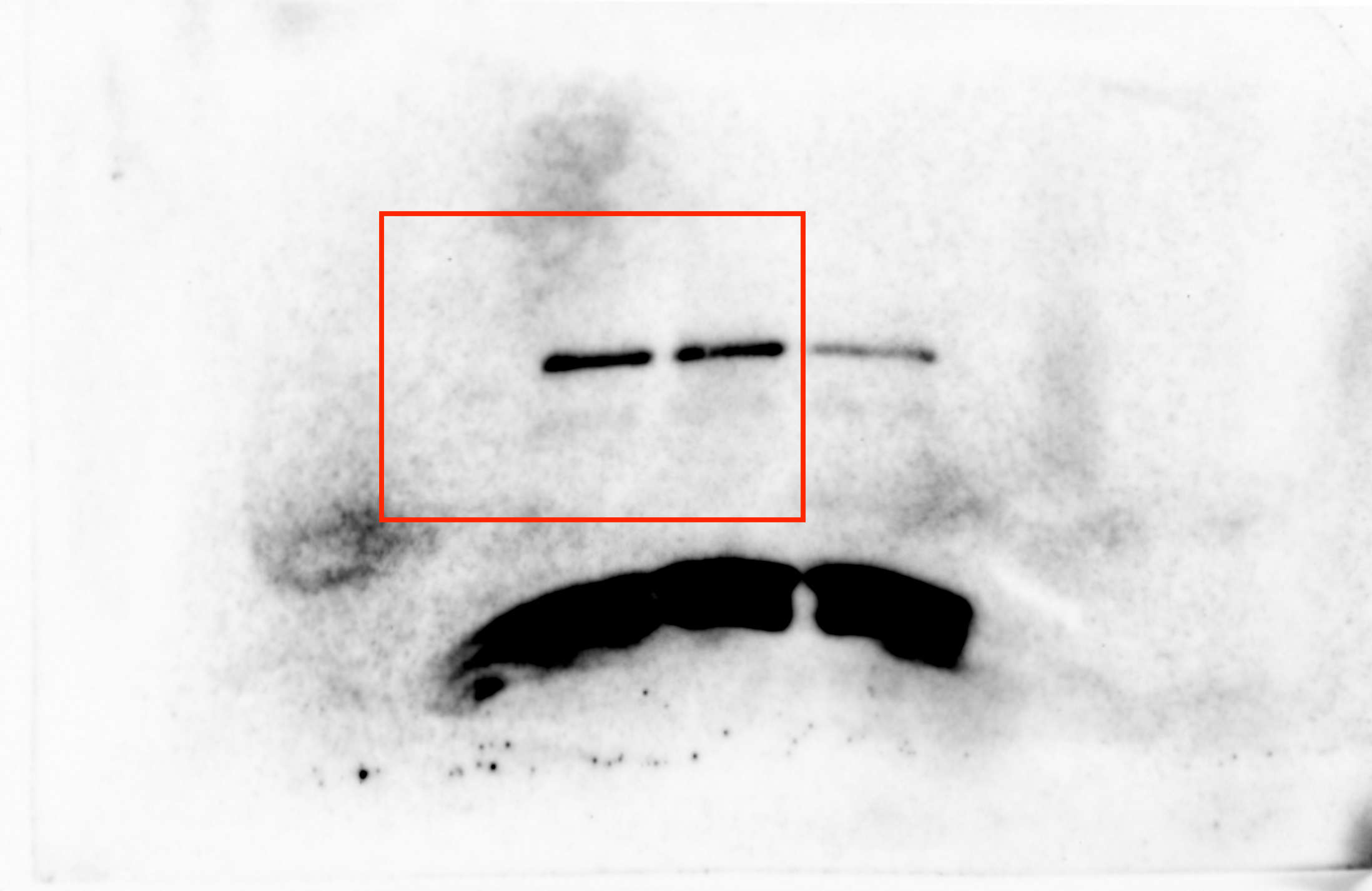

Supplement: Supplementary file 6 — Source Data Fig. 3 [file 44318_2024_71_MOESM6_ESM.zip › Fig 3/B/Figure_3B_WB_AGO1_IP.tif]

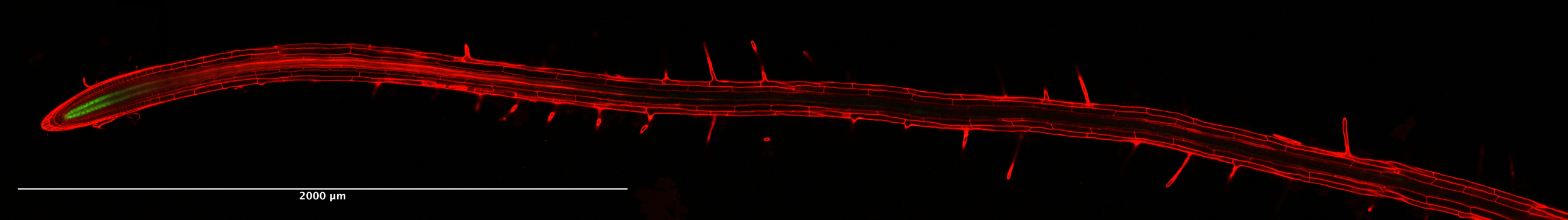

Supplement: Supplementary file 7 — Source Data Fig. 4 [file 44318_2024_71_MOESM7_ESM.zip › Fig 4/D/A10 NX.jpg]

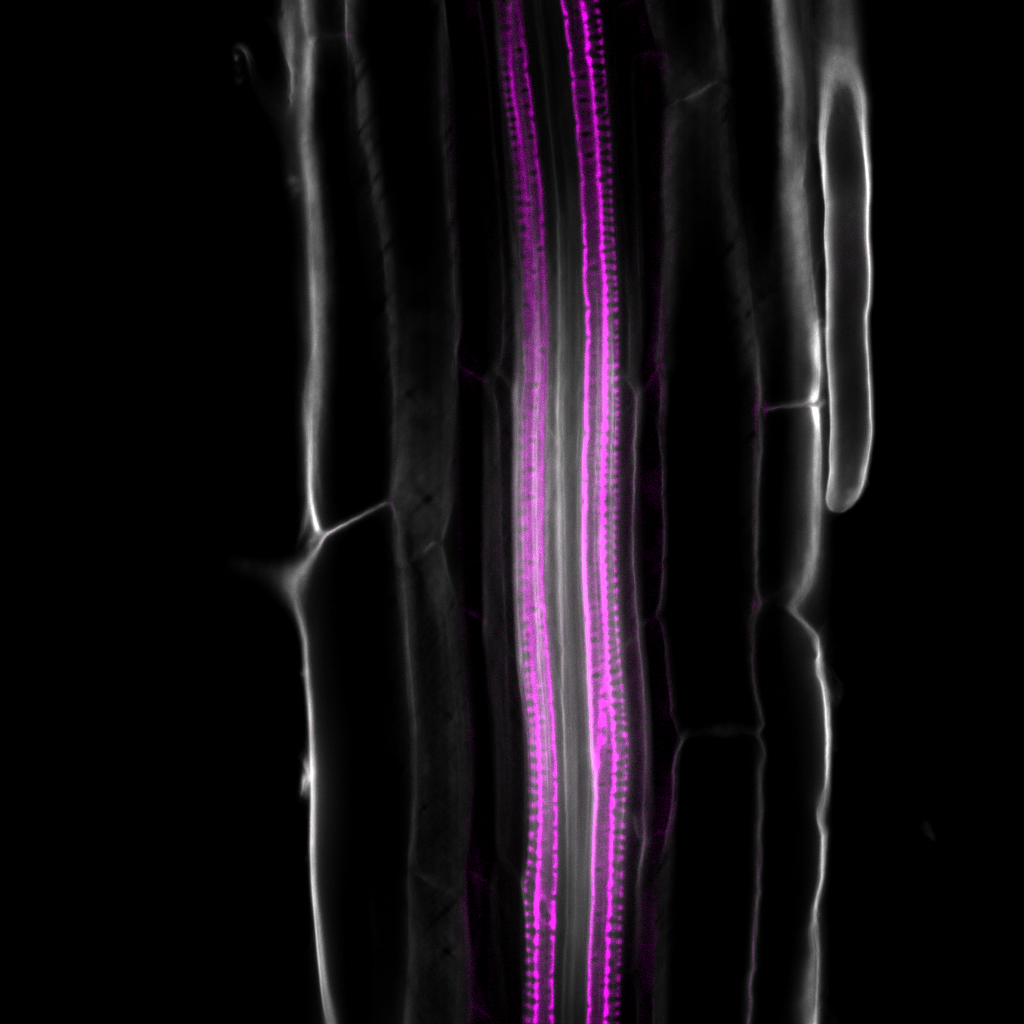

Supplement: Supplementary file 7 — Source Data Fig. 4 [file 44318_2024_71_MOESM7_ESM.zip › Fig 4/C/WT-QCP.tif]

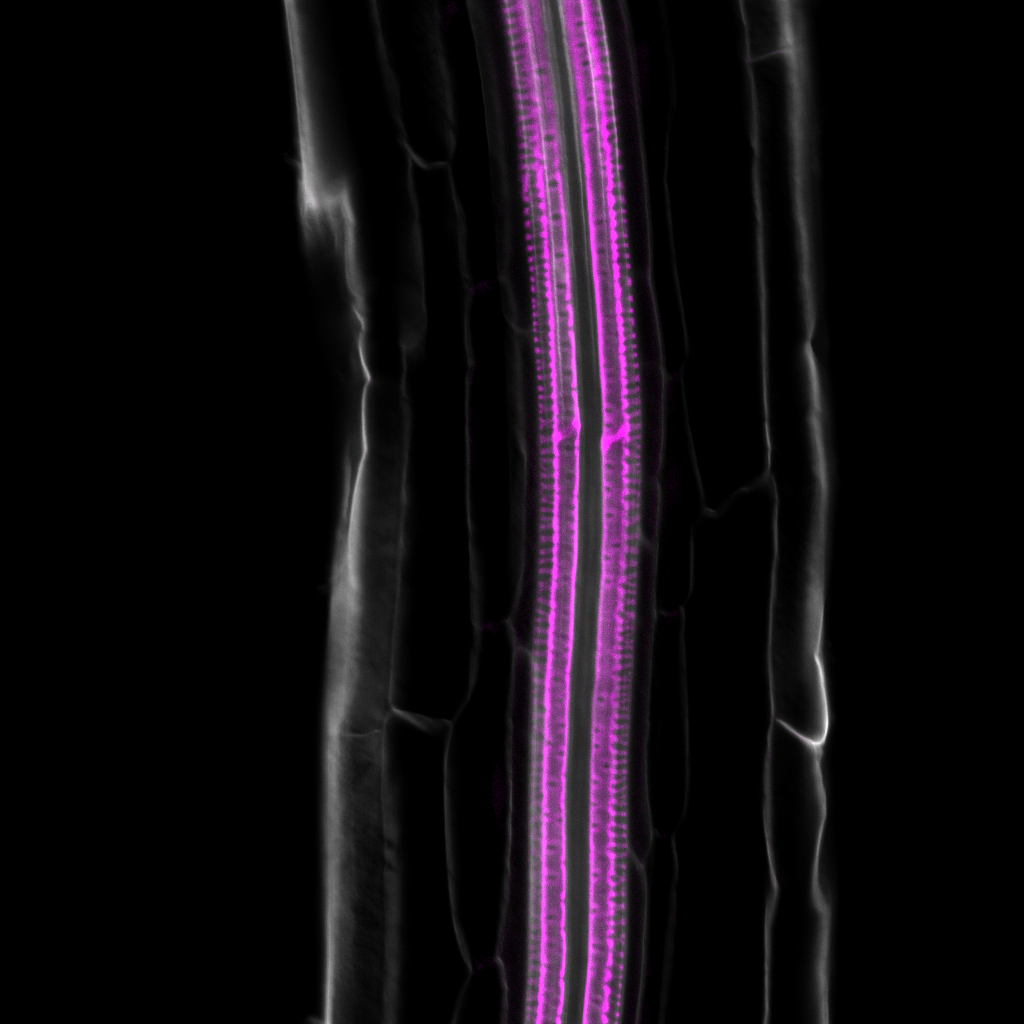

Supplement: Supplementary file 7 — Source Data Fig. 4 [file 44318_2024_71_MOESM7_ESM.zip › Fig 4/C/WT-QCD.tif]

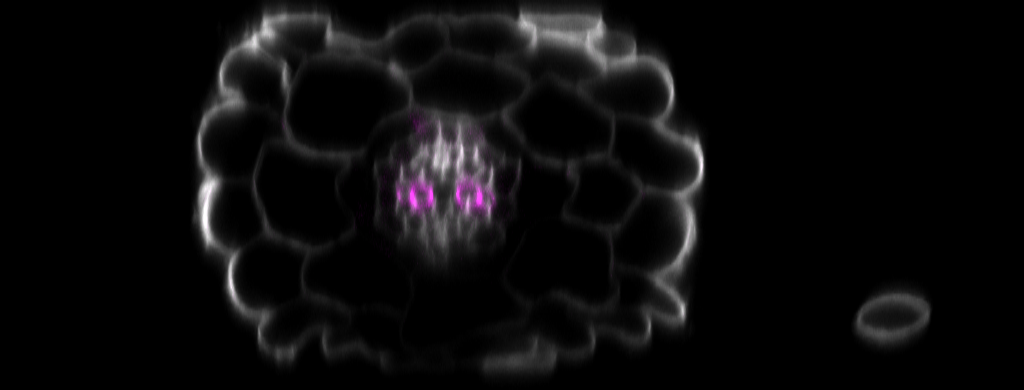

Supplement: Supplementary file 7 — Source Data Fig. 4 [file 44318_2024_71_MOESM7_ESM.zip › Fig 4/C/WT-QCD-CS.tif]

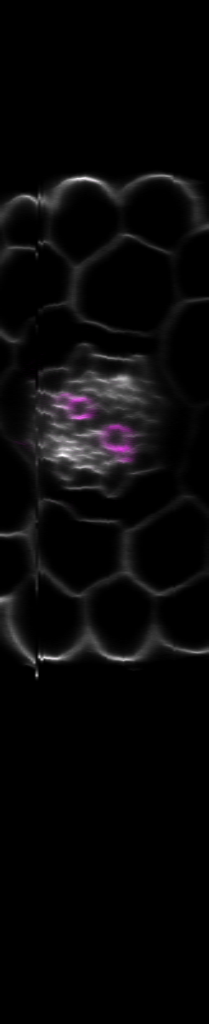

Supplement: Supplementary file 7 — Source Data Fig. 4 [file 44318_2024_71_MOESM7_ESM.zip › Fig 4/C/WT-QCP-CS.tif]

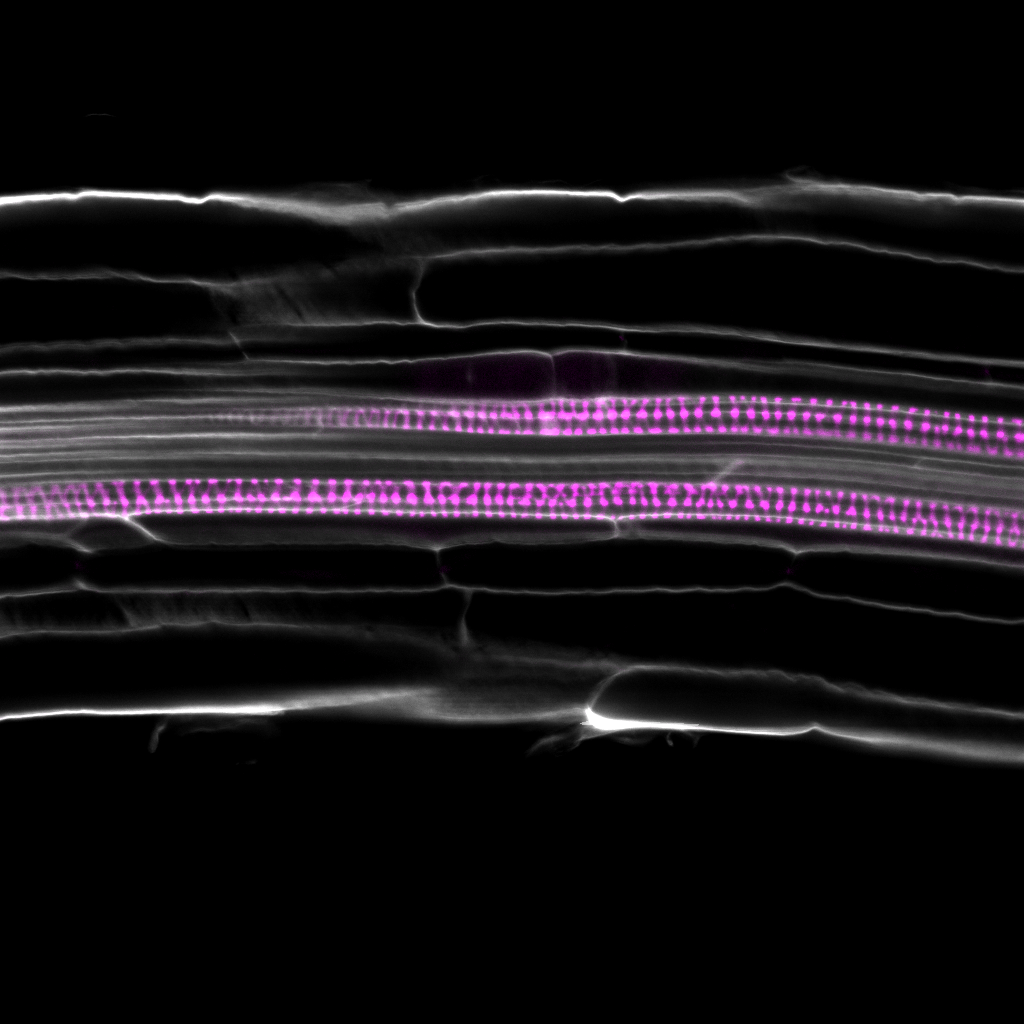

Supplement: Supplementary file 7 — Source Data Fig. 4 [file 44318_2024_71_MOESM7_ESM.zip › Fig 4/E/QCP-1.tif]

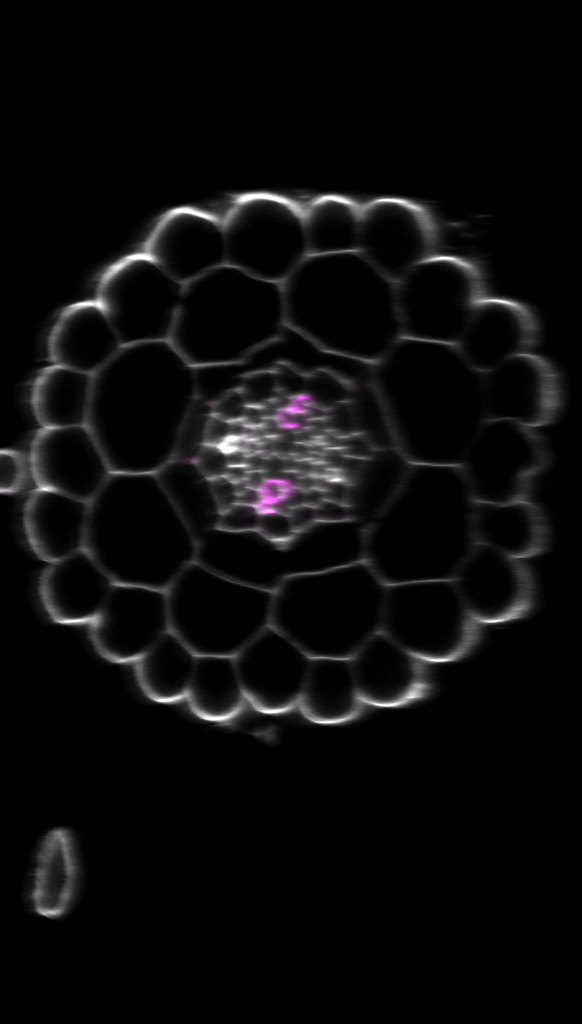

Supplement: Supplementary file 7 — Source Data Fig. 4 [file 44318_2024_71_MOESM7_ESM.zip › Fig 4/E/QCP-1-CS.tif]

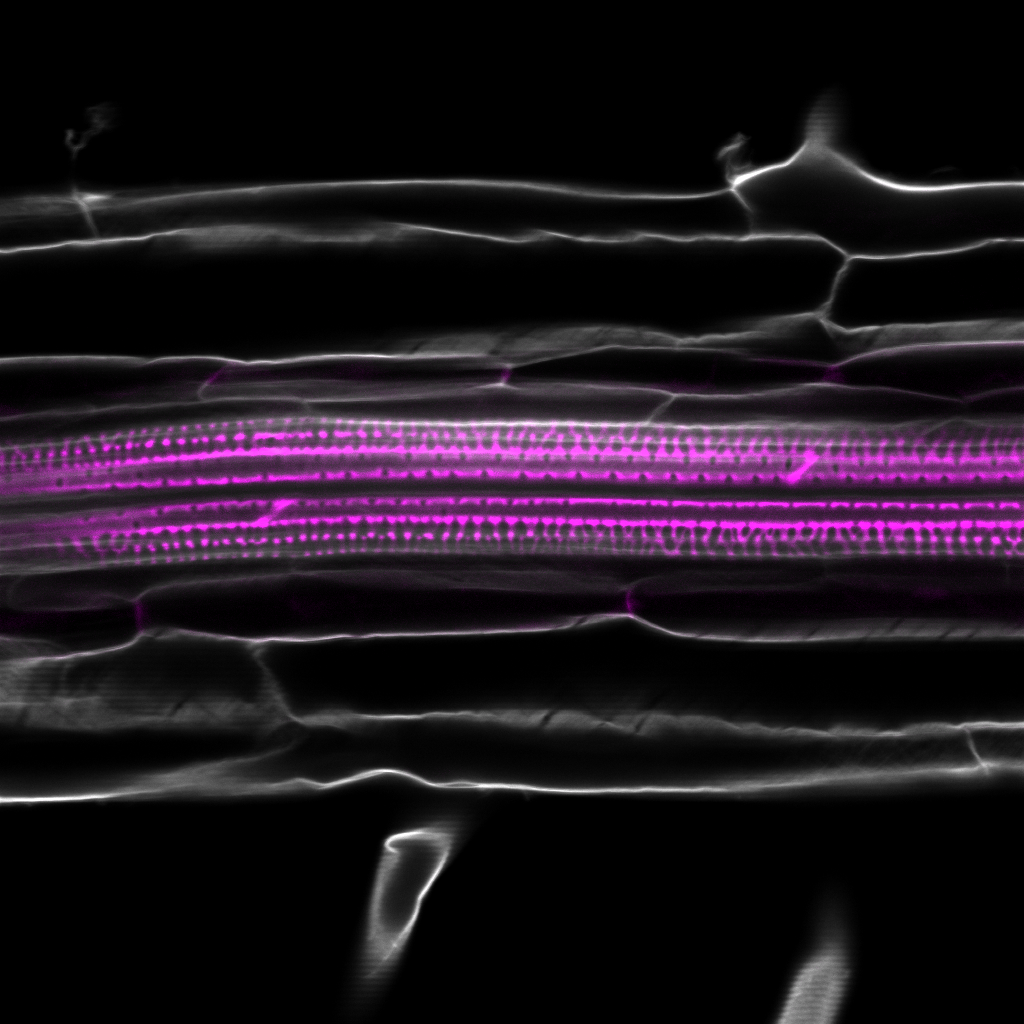

Supplement: Supplementary file 7 — Source Data Fig. 4 [file 44318_2024_71_MOESM7_ESM.zip › Fig 4/E/QCP-3.tif]

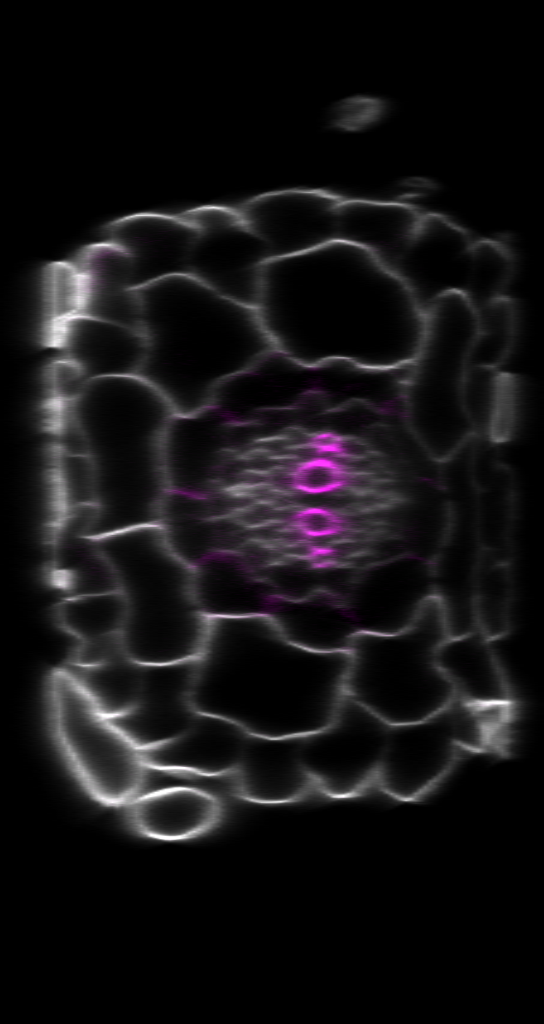

Supplement: Supplementary file 7 — Source Data Fig. 4 [file 44318_2024_71_MOESM7_ESM.zip › Fig 4/E/QCP-3-CS.tif]

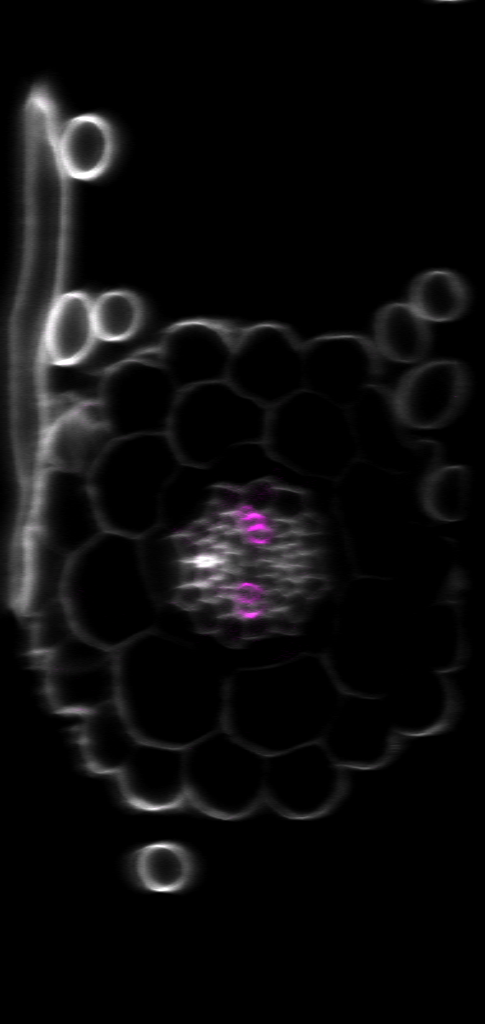

Supplement: Supplementary file 7 — Source Data Fig. 4 [file 44318_2024_71_MOESM7_ESM.zip › Fig 4/E/QCP-2-CS.tif]

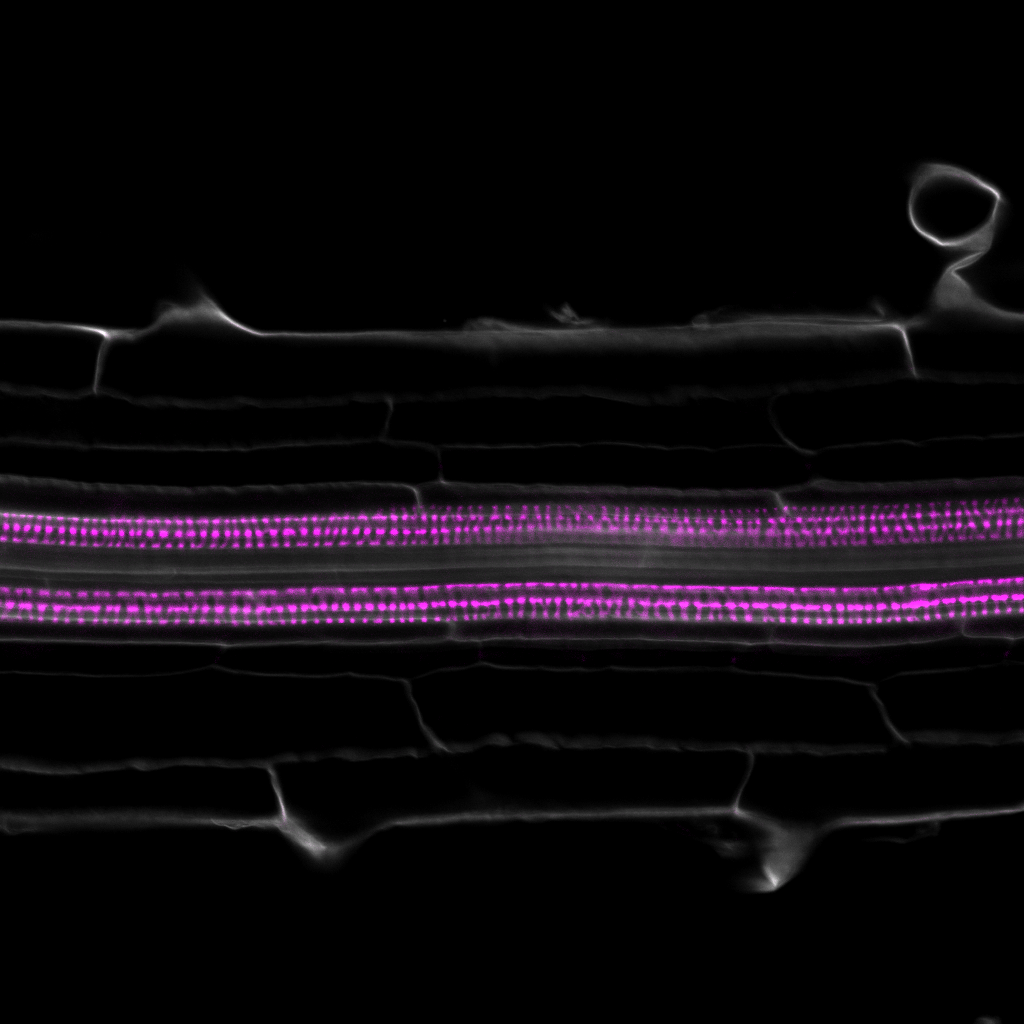

Supplement: Supplementary file 7 — Source Data Fig. 4 [file 44318_2024_71_MOESM7_ESM.zip › Fig 4/E/QCP-2.tif]

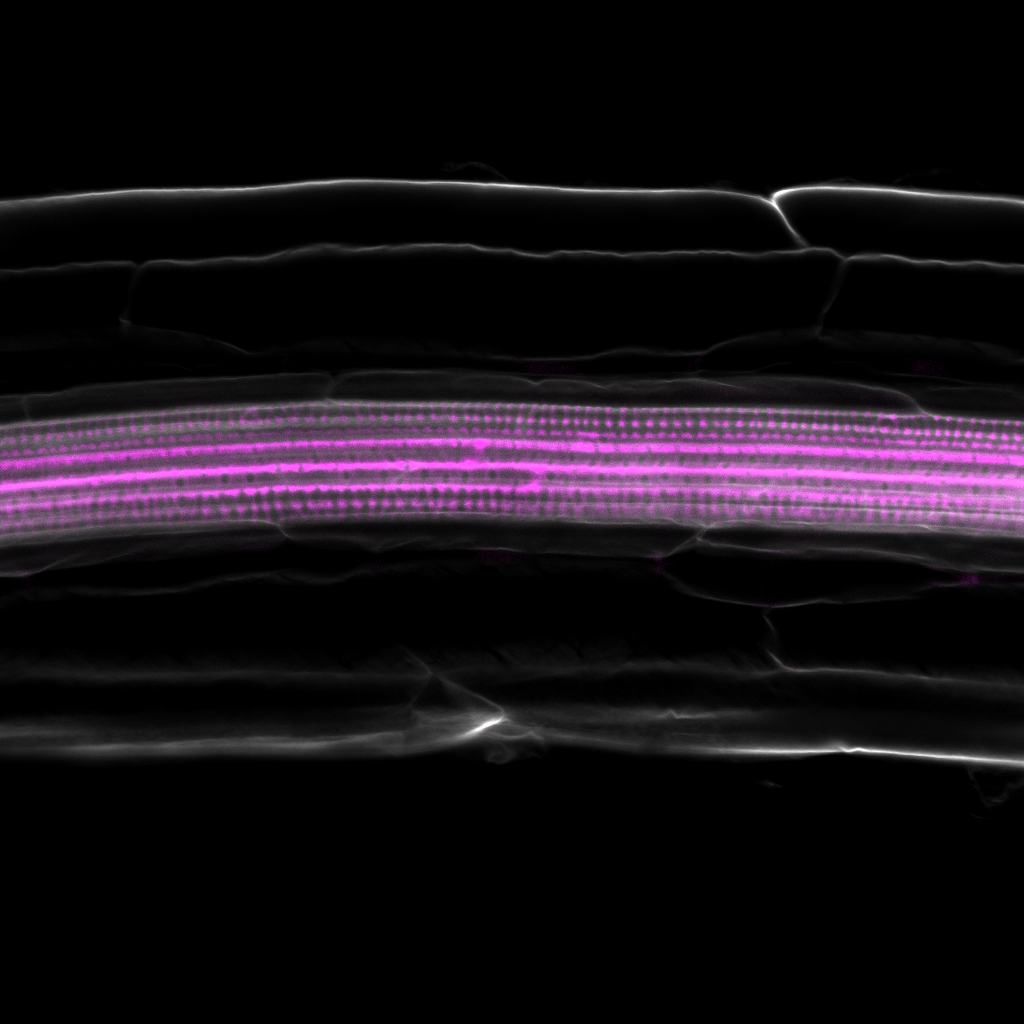

Supplement: Supplementary file 7 — Source Data Fig. 4 [file 44318_2024_71_MOESM7_ESM.zip › Fig 4/E/QCD-2.tif]

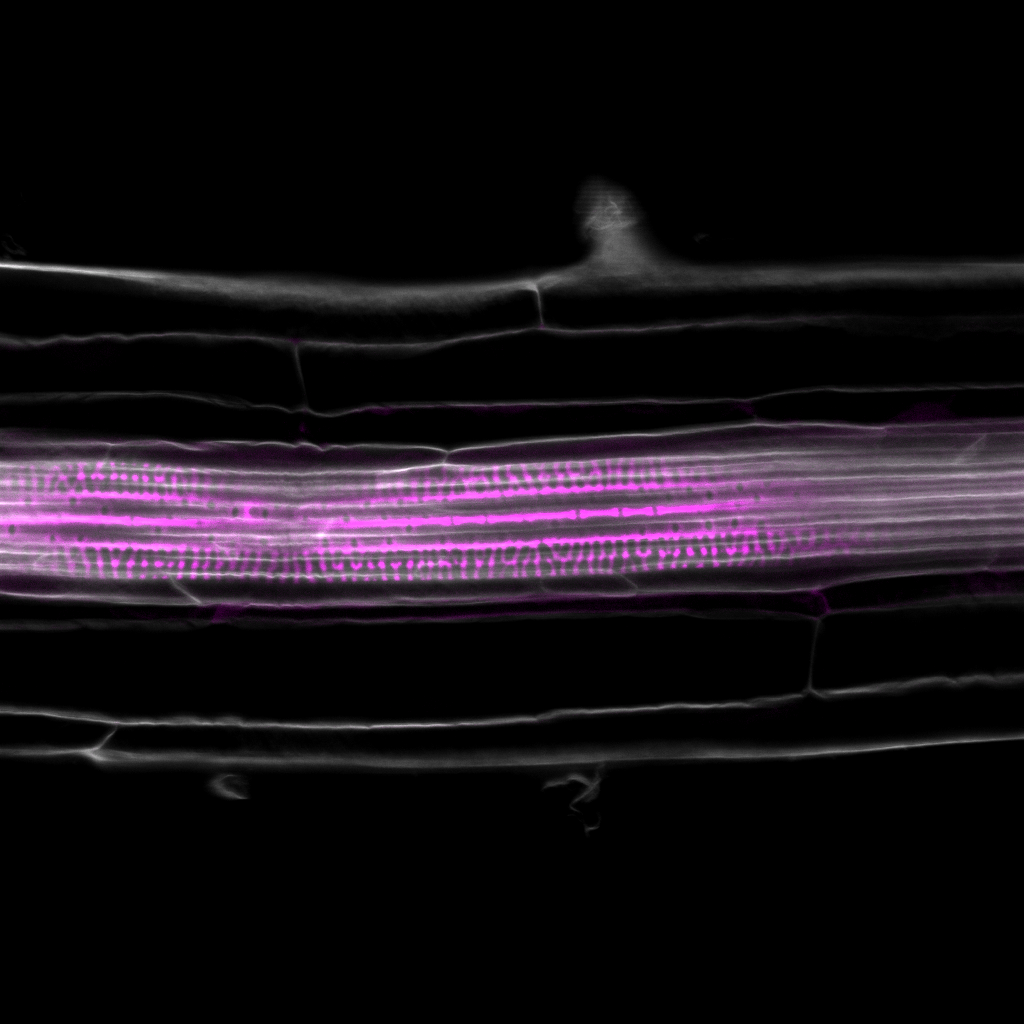

Supplement: Supplementary file 7 — Source Data Fig. 4 [file 44318_2024_71_MOESM7_ESM.zip › Fig 4/E/QCD-1.tif]

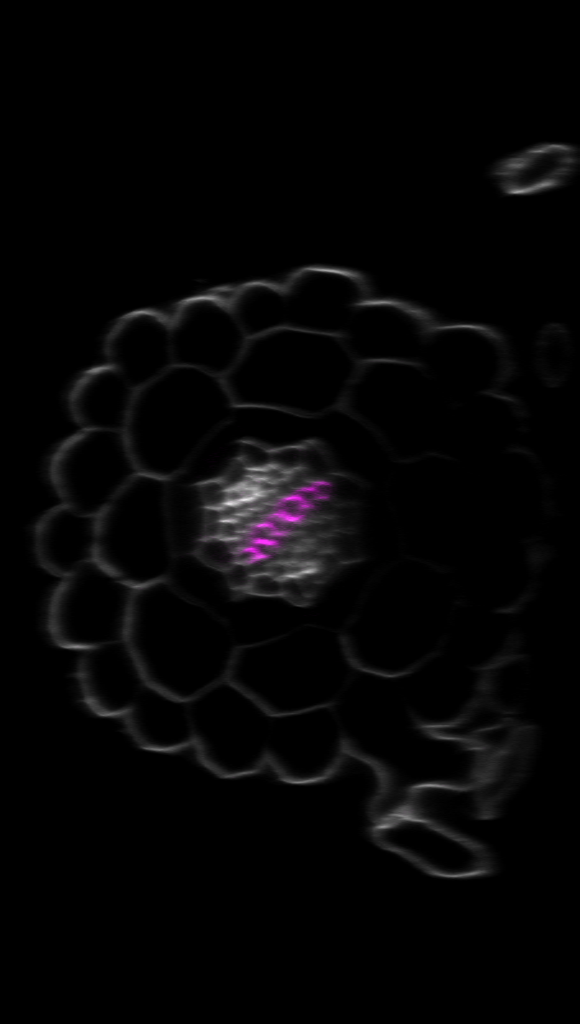

Supplement: Supplementary file 7 — Source Data Fig. 4 [file 44318_2024_71_MOESM7_ESM.zip › Fig 4/E/QCD-2-CS.tif]

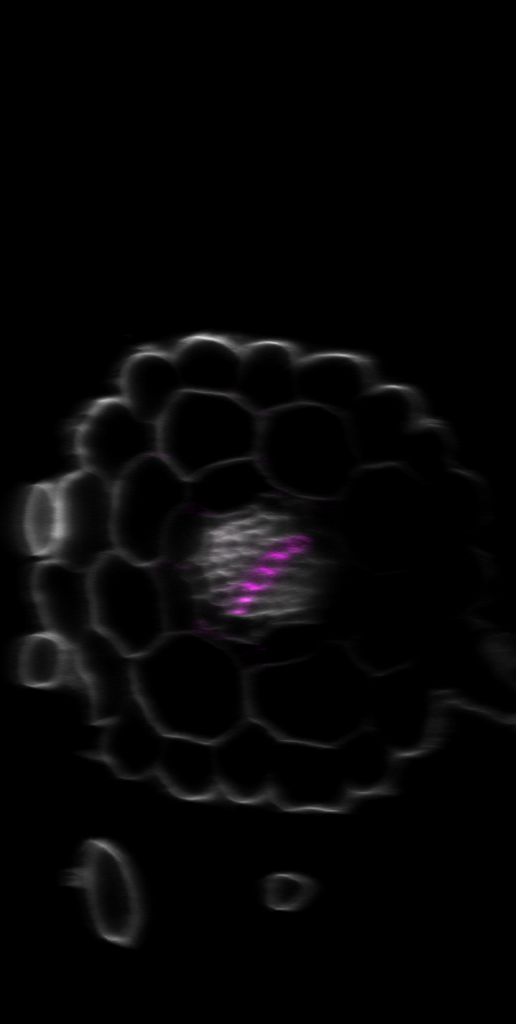

Supplement: Supplementary file 7 — Source Data Fig. 4 [file 44318_2024_71_MOESM7_ESM.zip › Fig 4/E/QCD-1-CS.tif]

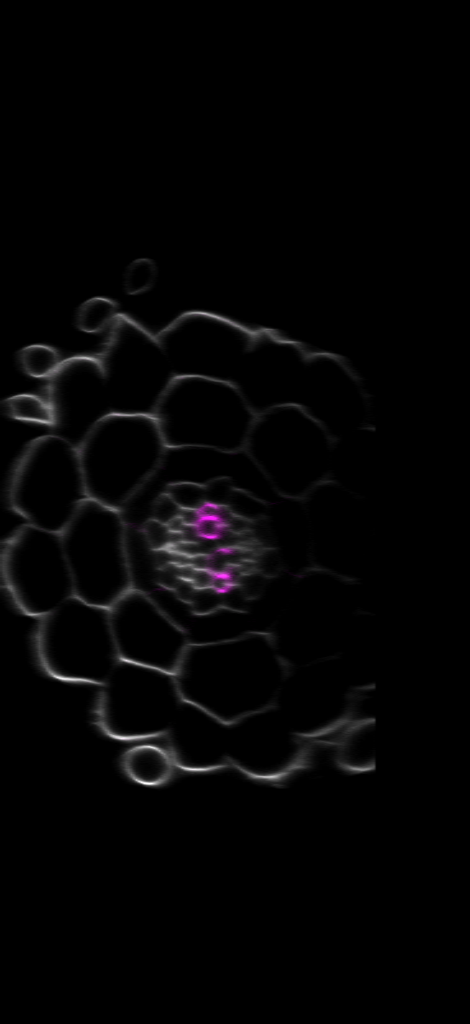

Supplement: Supplementary file 7 — Source Data Fig. 4 [file 44318_2024_71_MOESM7_ESM.zip › Fig 4/F/er2-QCP-CS.tif]

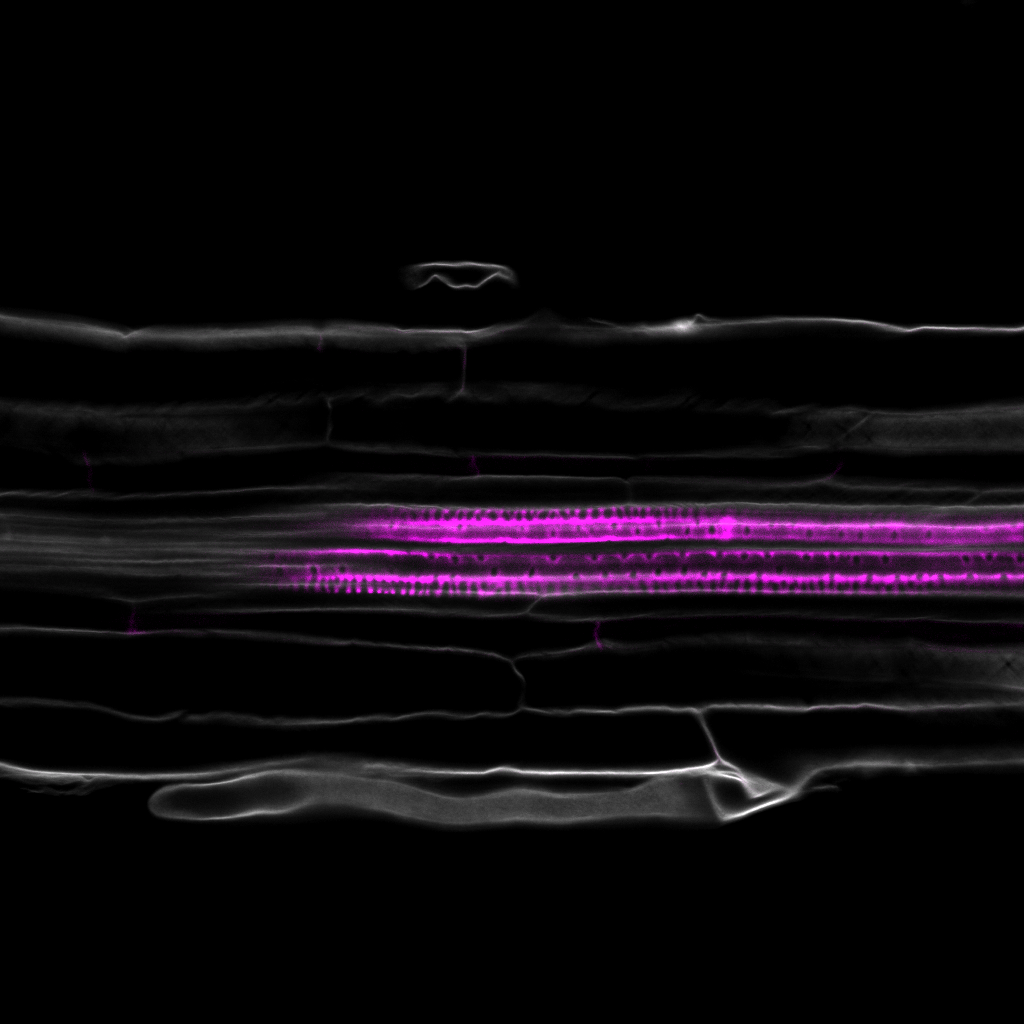

Supplement: Supplementary file 7 — Source Data Fig. 4 [file 44318_2024_71_MOESM7_ESM.zip › Fig 4/F/er2-QCP.tif]

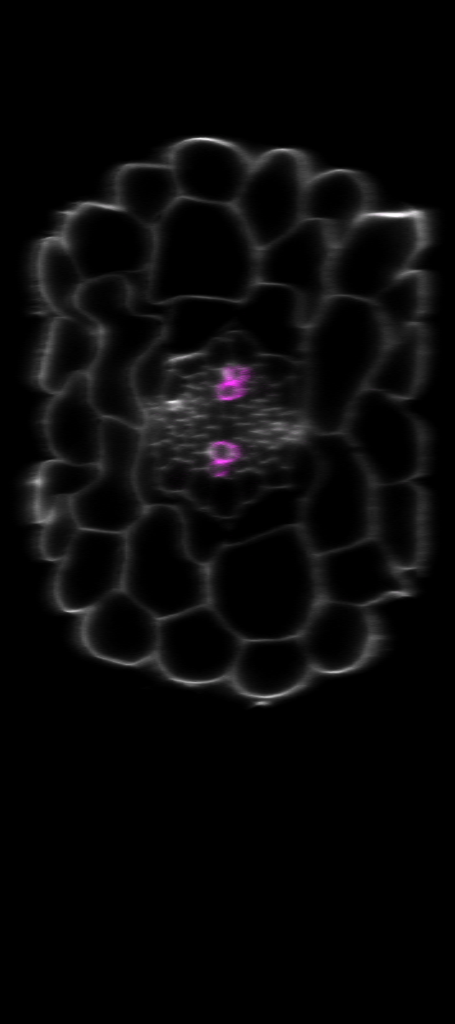

Supplement: Supplementary file 7 — Source Data Fig. 4 [file 44318_2024_71_MOESM7_ESM.zip › Fig 4/F/phbphvcna-QCP-CS.tif]

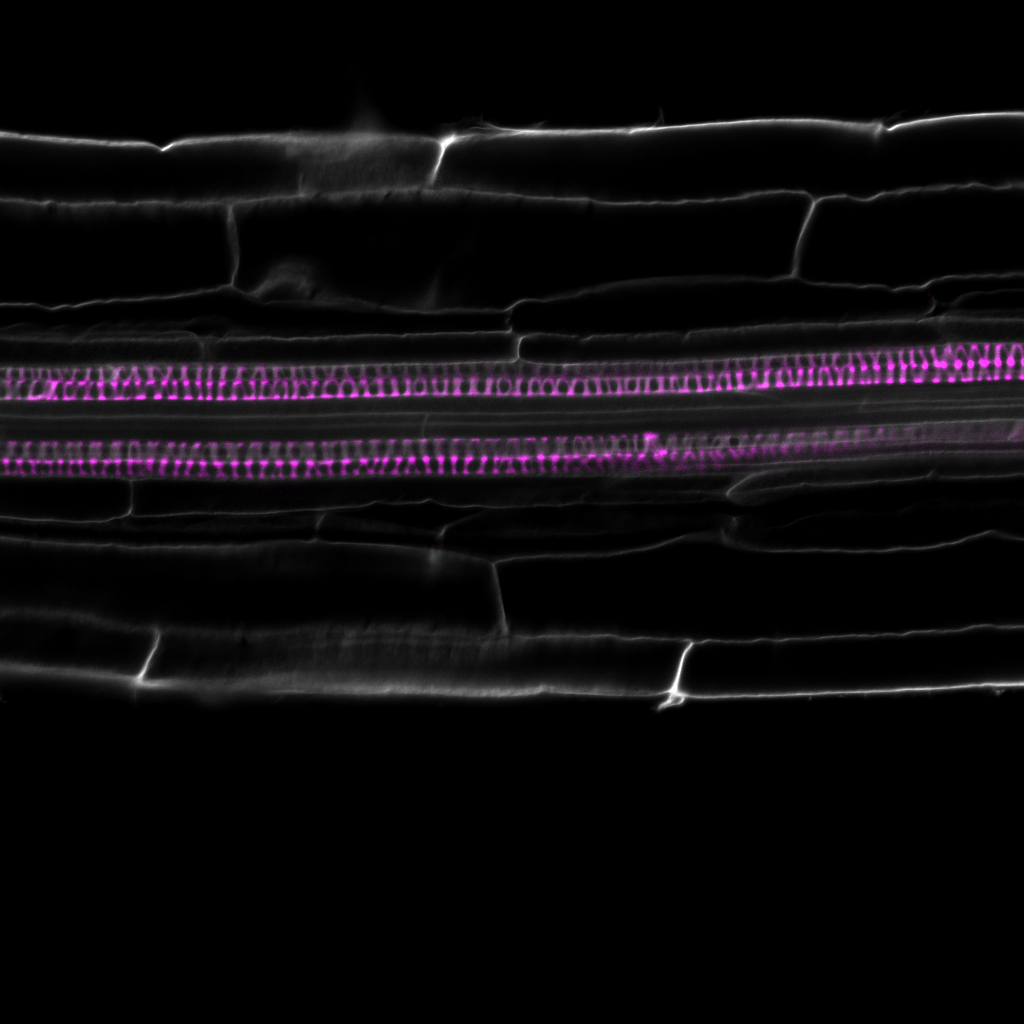

Supplement: Supplementary file 7 — Source Data Fig. 4 [file 44318_2024_71_MOESM7_ESM.zip › Fig 4/F/phbphvcna-QCP.tif]

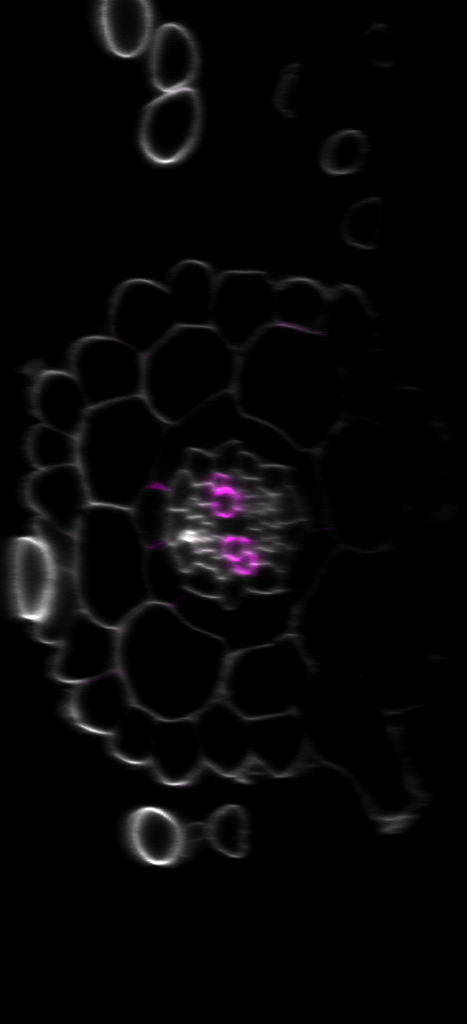

Supplement: Supplementary file 7 — Source Data Fig. 4 [file 44318_2024_71_MOESM7_ESM.zip › Fig 4/F/er2-QCD-CS.tif]

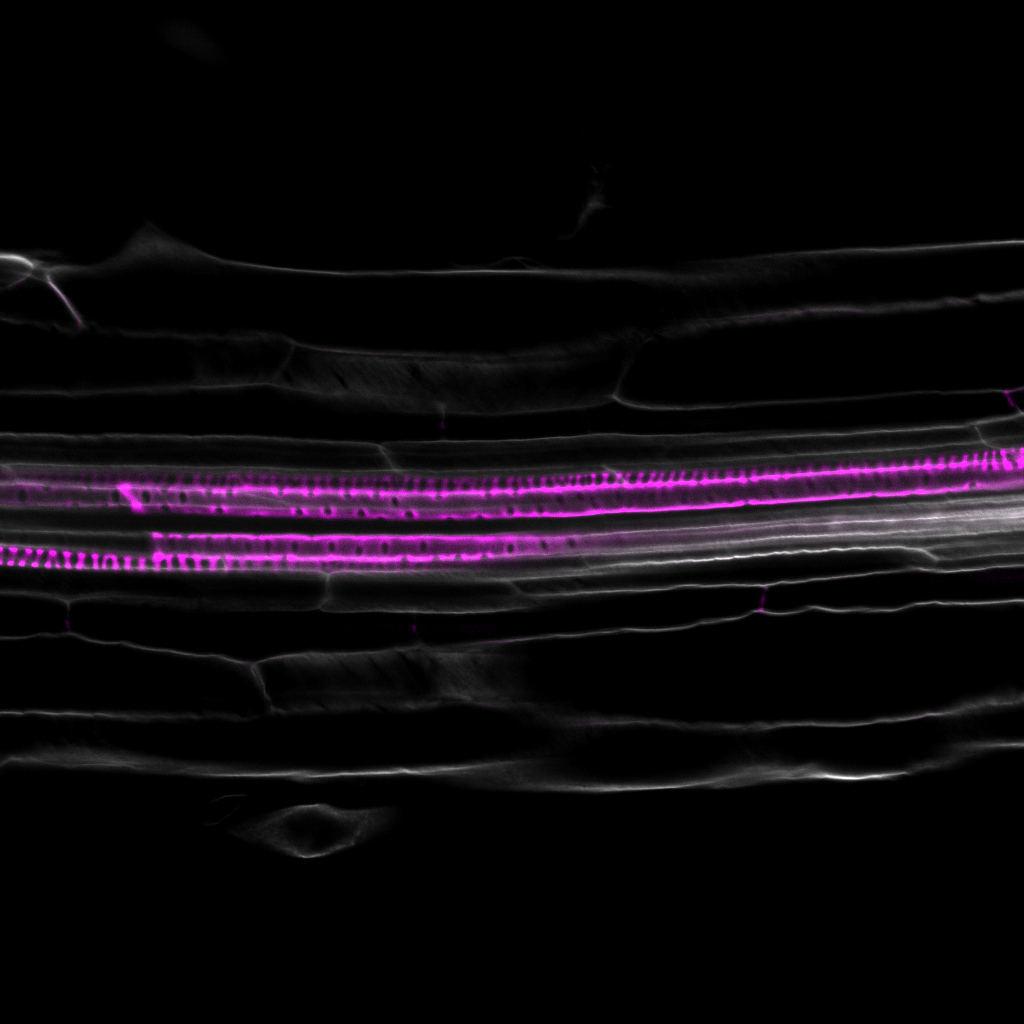

Supplement: Supplementary file 7 — Source Data Fig. 4 [file 44318_2024_71_MOESM7_ESM.zip › Fig 4/F/er2-QCD.tif]

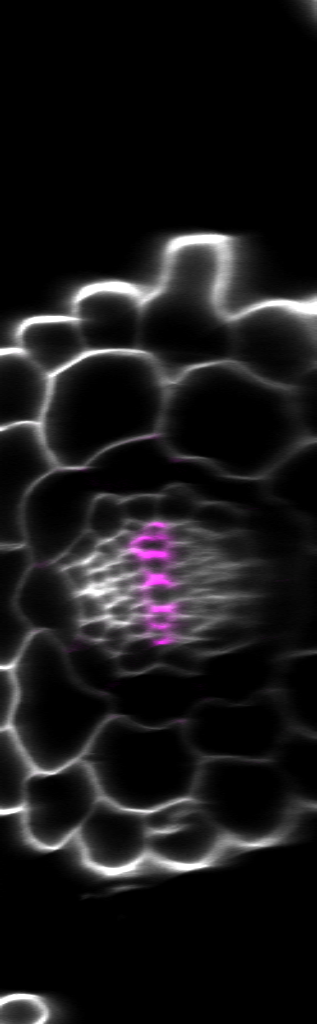

Supplement: Supplementary file 7 — Source Data Fig. 4 [file 44318_2024_71_MOESM7_ESM.zip › Fig 4/F/phbphvcna-QCD-CS.tif]

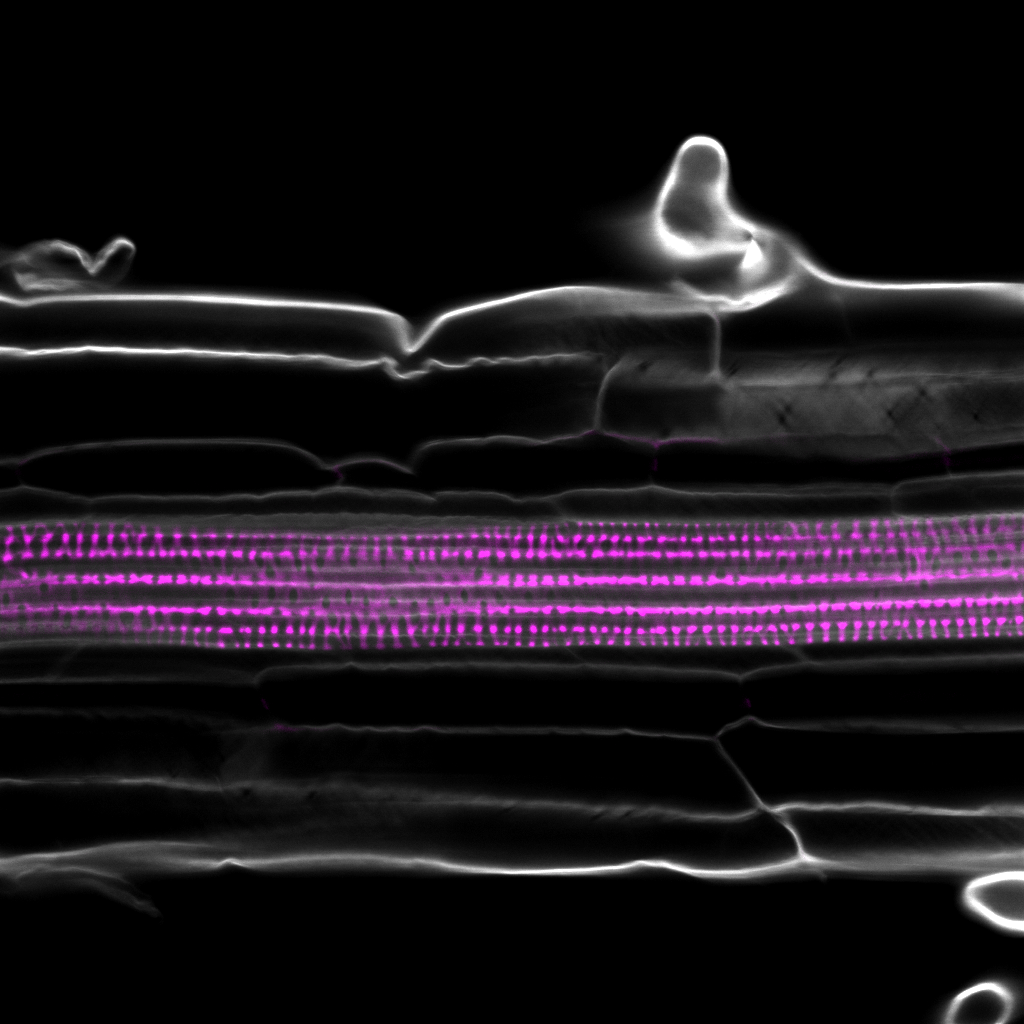

Supplement: Supplementary file 7 — Source Data Fig. 4 [file 44318_2024_71_MOESM7_ESM.zip › Fig 4/F/phbphvcna-QCD.tif]

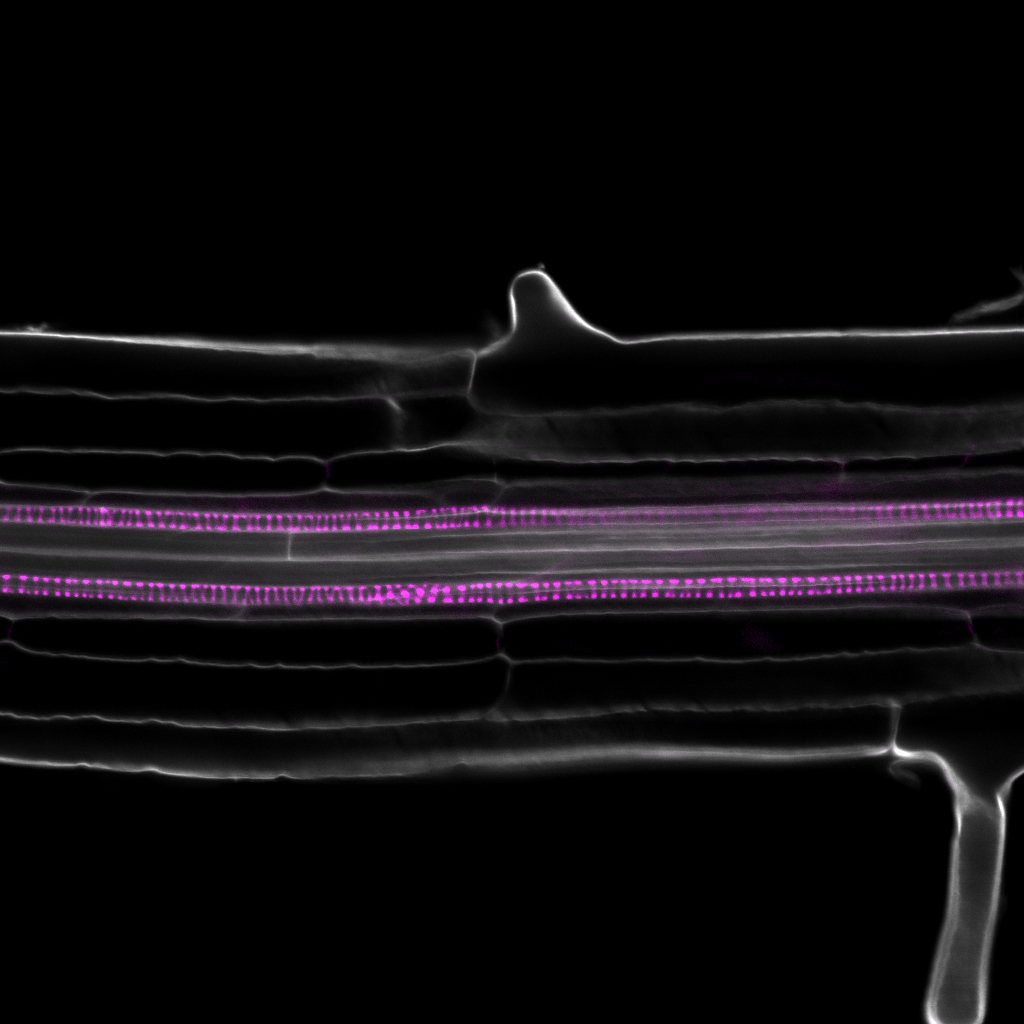

Supplement: Supplementary file 7 — Source Data Fig. 4 [file 44318_2024_71_MOESM7_ESM.zip › Fig 4/G/sdn-QCP.tif]

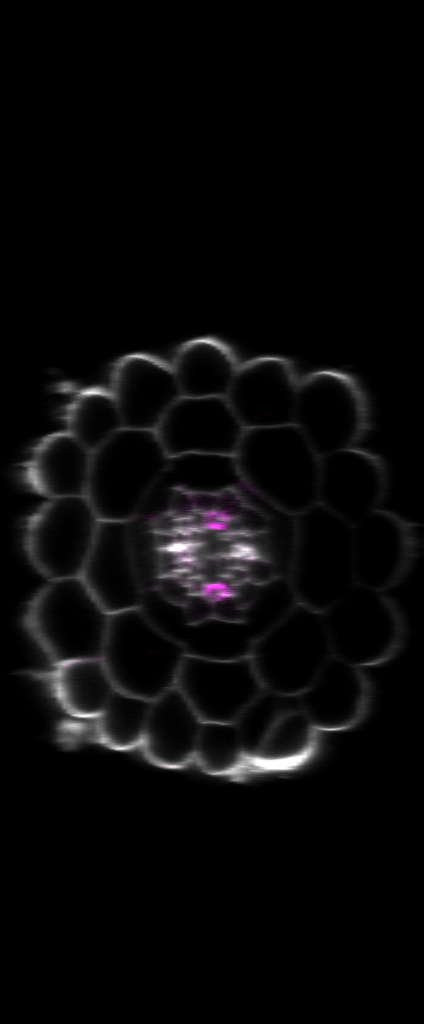

Supplement: Supplementary file 7 — Source Data Fig. 4 [file 44318_2024_71_MOESM7_ESM.zip › Fig 4/G/sdn-QCP-CS.tif]

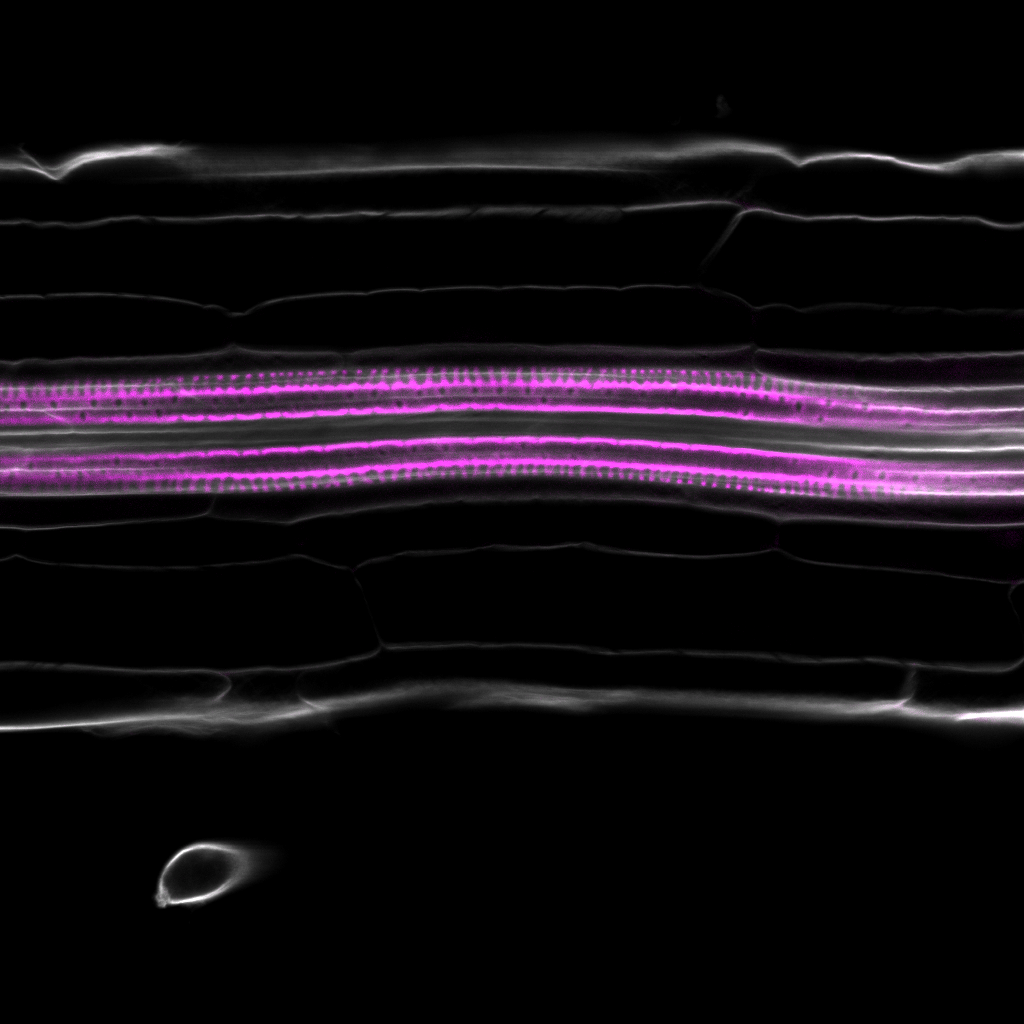

Supplement: Supplementary file 7 — Source Data Fig. 4 [file 44318_2024_71_MOESM7_ESM.zip › Fig 4/G/sdn-QCD-2.tif]

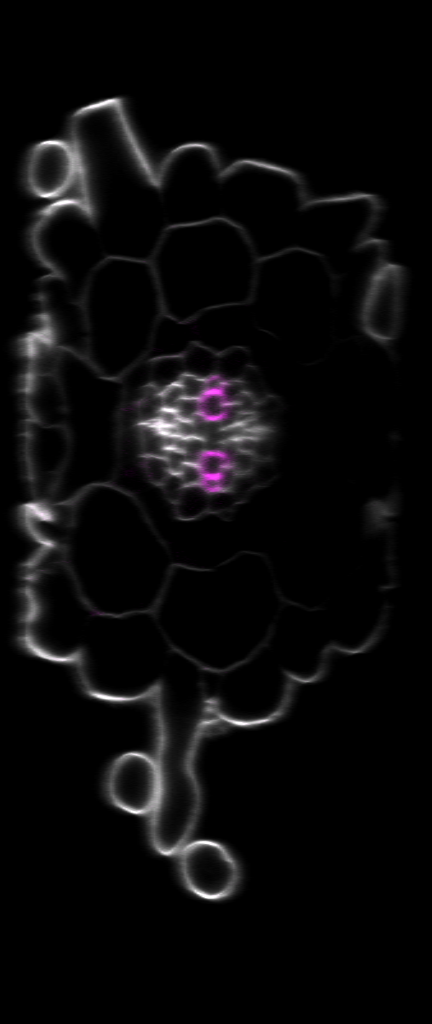

Supplement: Supplementary file 7 — Source Data Fig. 4 [file 44318_2024_71_MOESM7_ESM.zip › Fig 4/G/sdn-QCD-2-CS.tif]

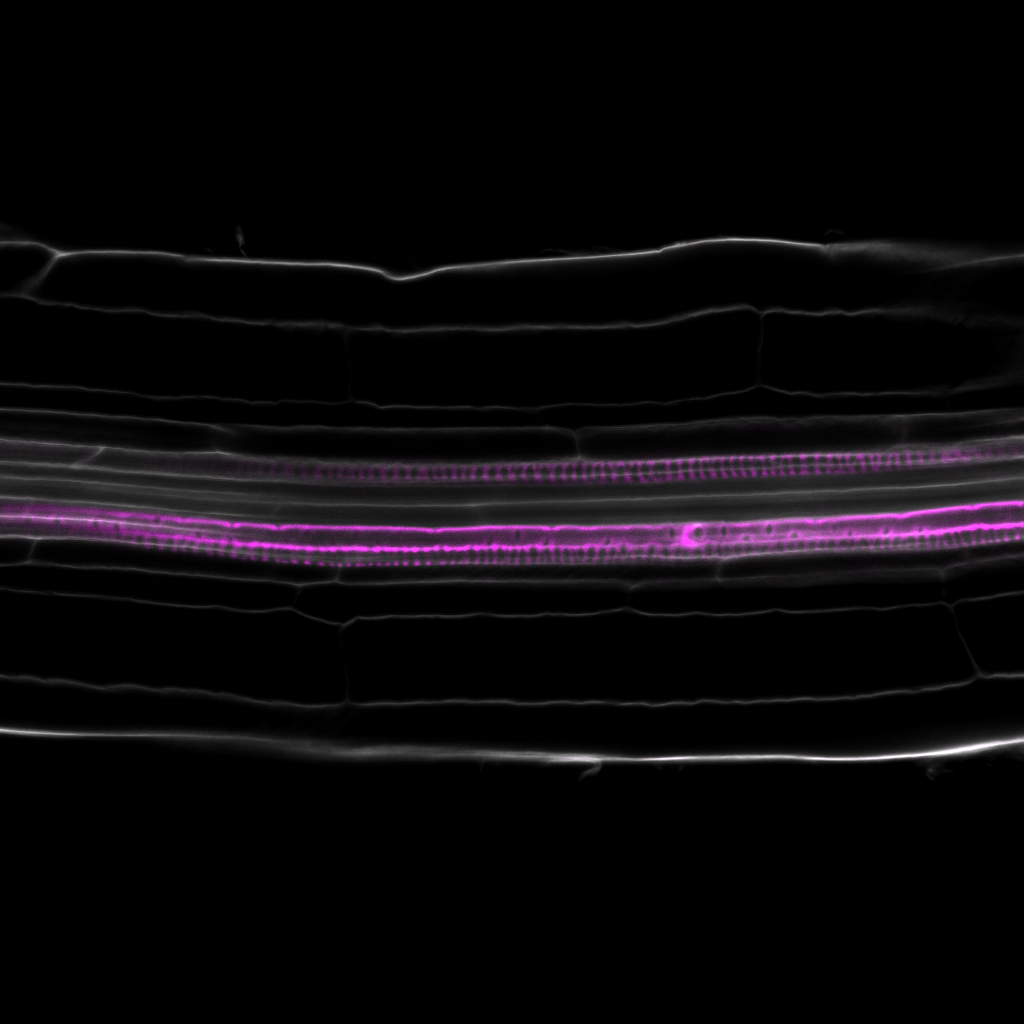

Supplement: Supplementary file 7 — Source Data Fig. 4 [file 44318_2024_71_MOESM7_ESM.zip › Fig 4/G/sdn-QCD-1.tif]

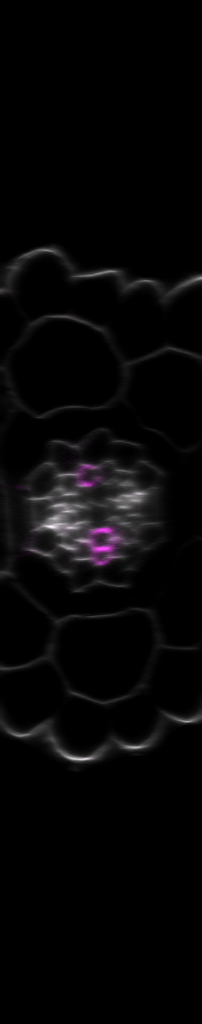

Supplement: Supplementary file 7 — Source Data Fig. 4 [file 44318_2024_71_MOESM7_ESM.zip › Fig 4/G/sdn-QCD-1-CS.tif]

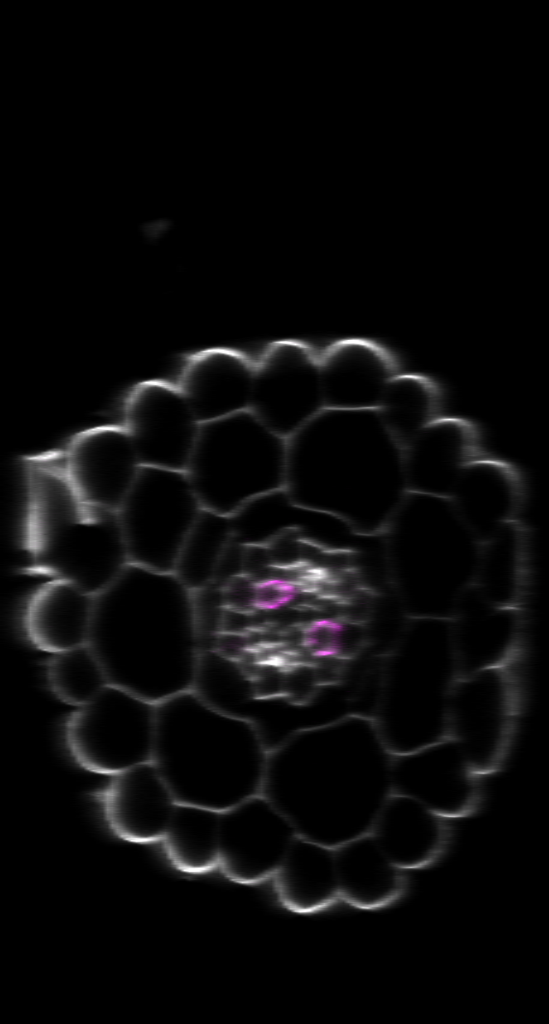

Supplement: Supplementary file 7 — Source Data Fig. 4 [file 44318_2024_71_MOESM7_ESM.zip › Fig 4/H/QCP-1-CS.tif]

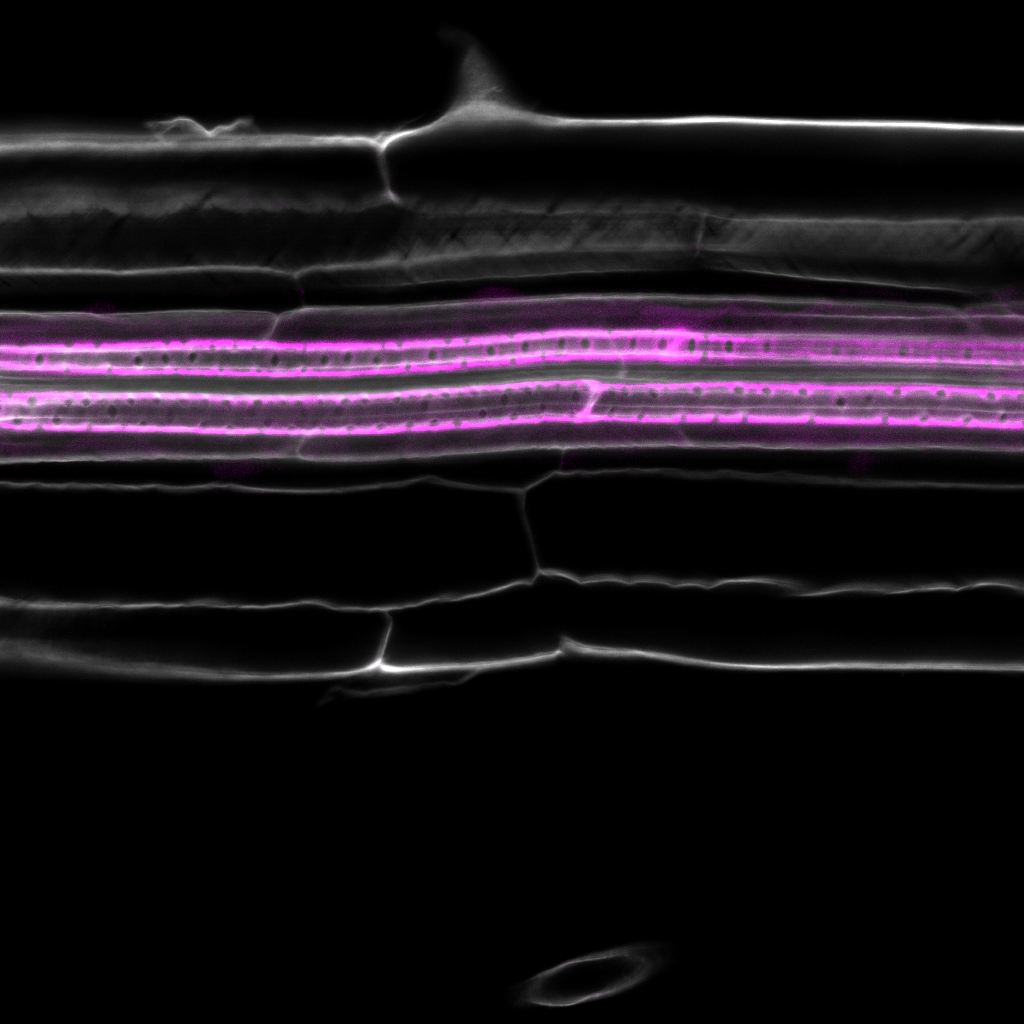

Supplement: Supplementary file 7 — Source Data Fig. 4 [file 44318_2024_71_MOESM7_ESM.zip › Fig 4/H/QCP-1.tif]

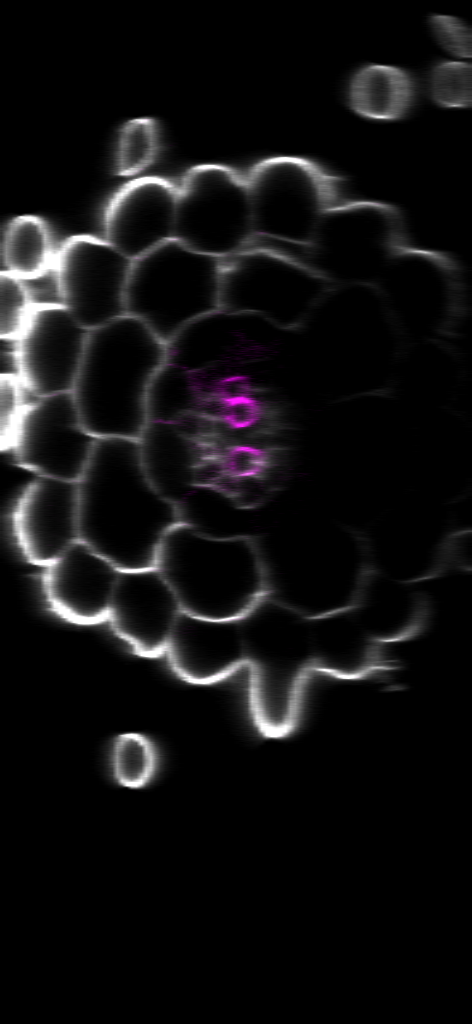

Supplement: Supplementary file 7 — Source Data Fig. 4 [file 44318_2024_71_MOESM7_ESM.zip › Fig 4/H/QCP-2-CS.tif]

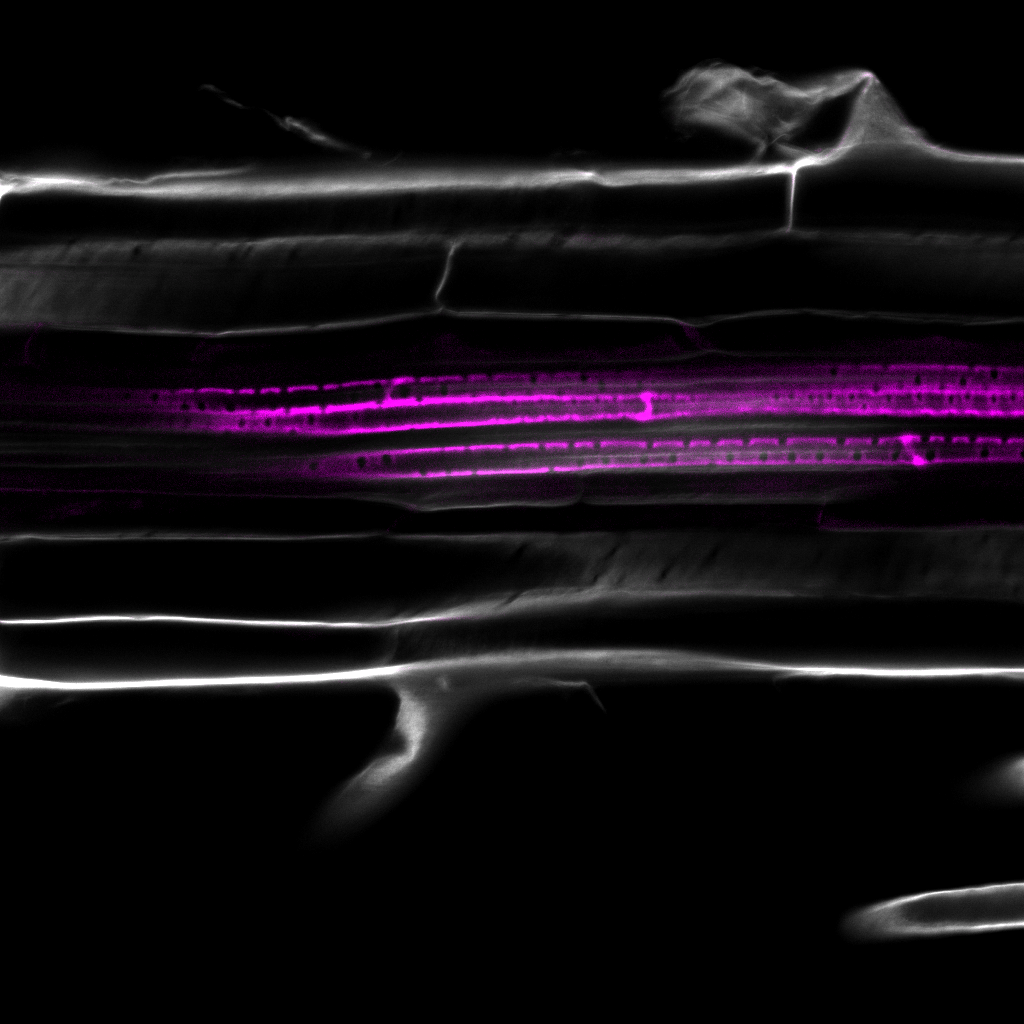

Supplement: Supplementary file 7 — Source Data Fig. 4 [file 44318_2024_71_MOESM7_ESM.zip › Fig 4/H/QCP-2.tif]

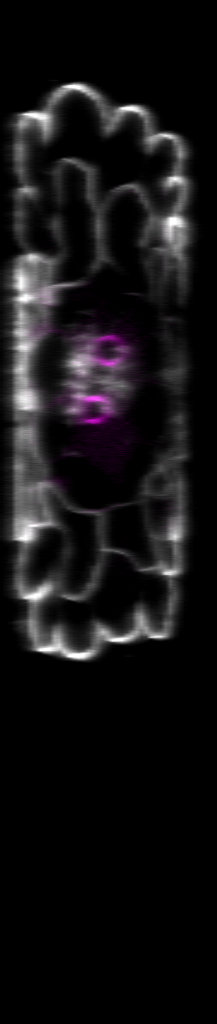

Supplement: Supplementary file 7 — Source Data Fig. 4 [file 44318_2024_71_MOESM7_ESM.zip › Fig 4/H/QCD-1-CS.tif]

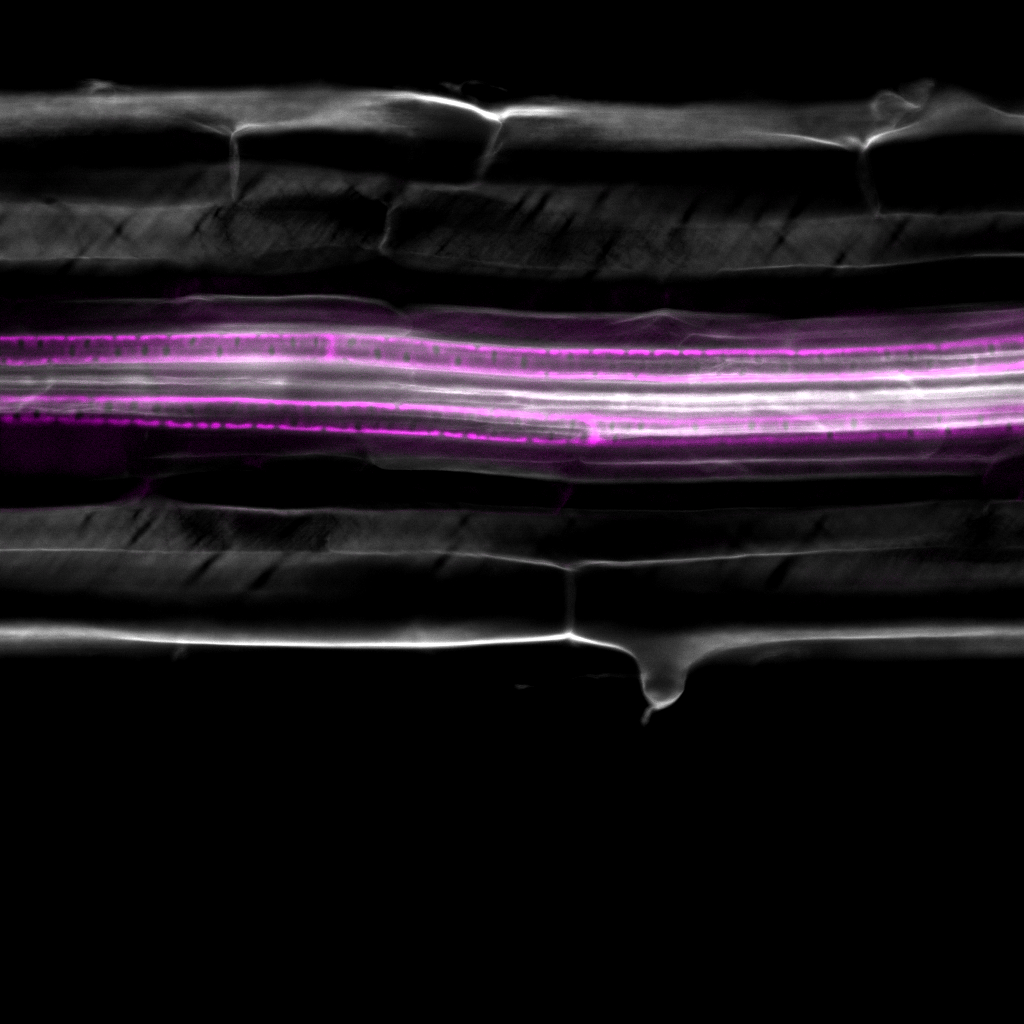

Supplement: Supplementary file 7 — Source Data Fig. 4 [file 44318_2024_71_MOESM7_ESM.zip › Fig 4/H/QCD-1.tif]
