# Supplementary figures and images for: An AGO10:miR165/6 module regulates meristem activity and xylem development in the Arabidopsis root (part 2 of 2)
Source: EMBO J. 2024 Apr 2;43(9):8. doi: 10.1038/s44318-024-00071-y (PMC11066010; doi:10.1038/s44318-024-00071-y)

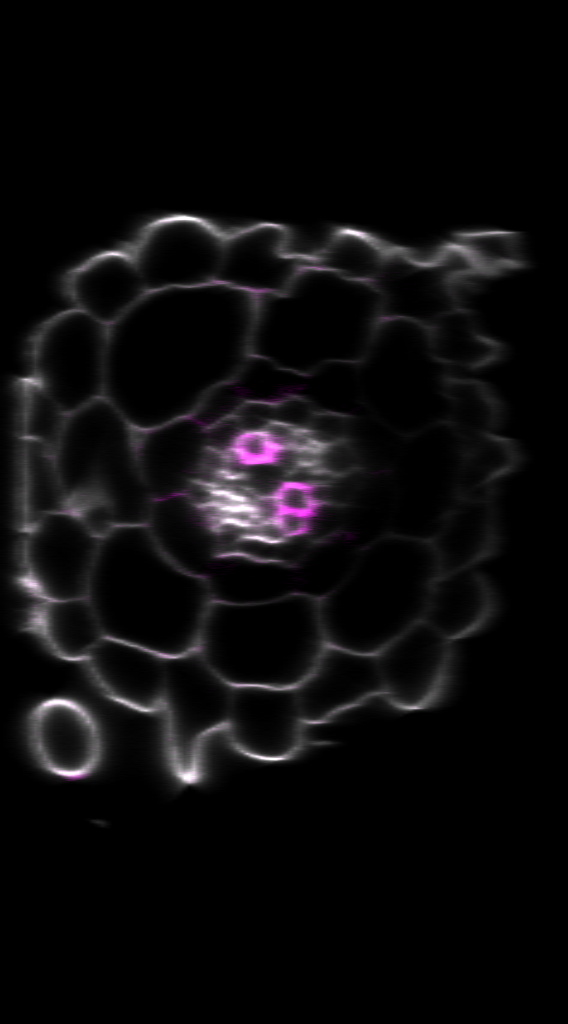

Supplement: Supplementary file 7 — Source Data Fig. 4 [file 44318_2024_71_MOESM7_ESM.zip › Fig 4/H/QCD-2-CS.tif]

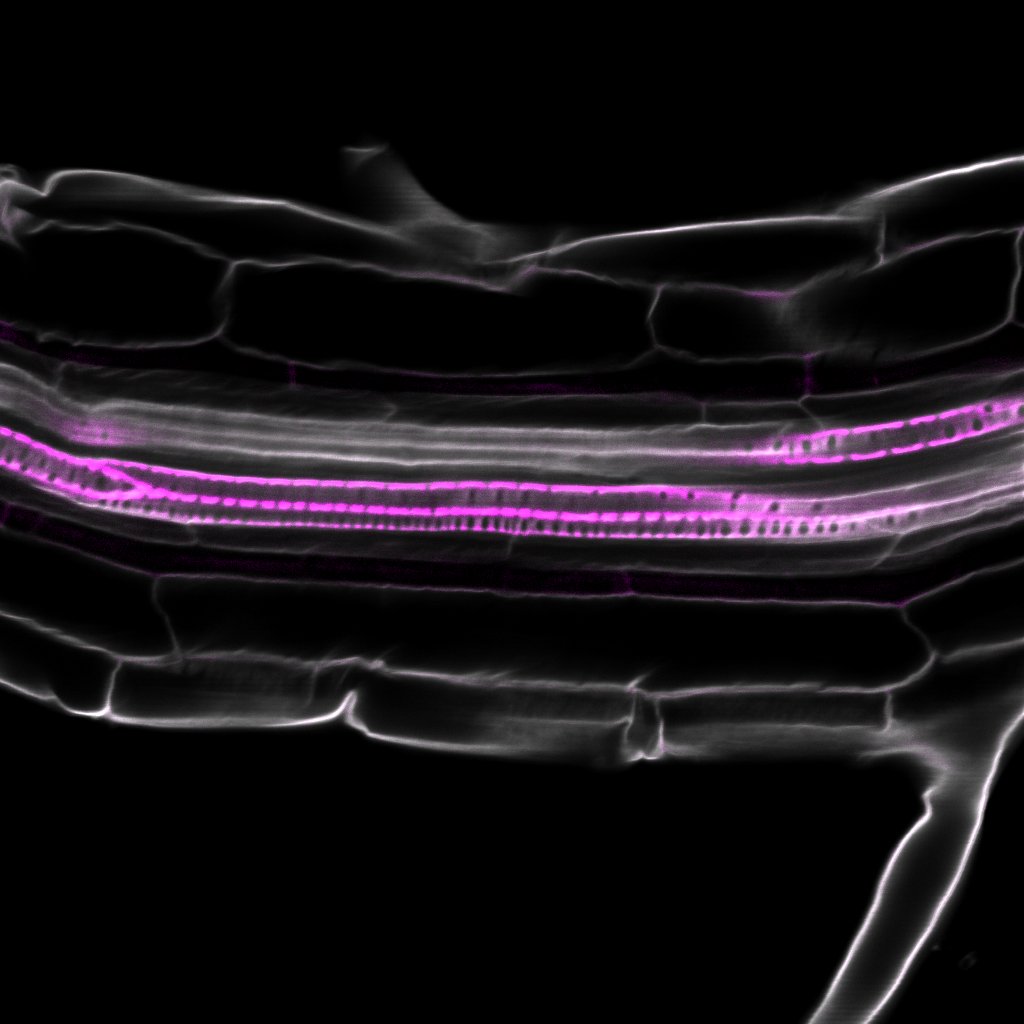

Supplement: Supplementary file 7 — Source Data Fig. 4 [file 44318_2024_71_MOESM7_ESM.zip › Fig 4/H/QCD-2.jpg]

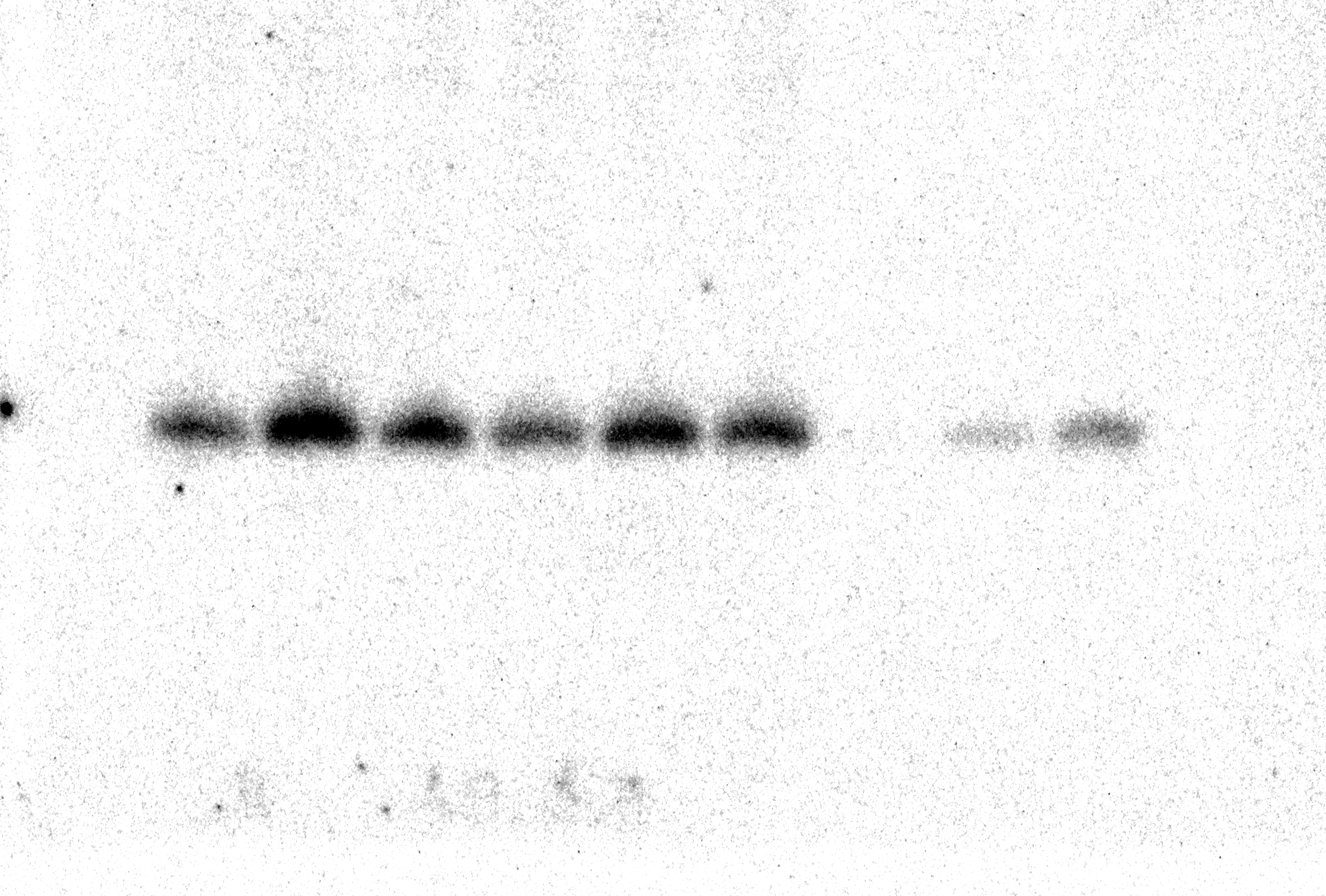

Supplement: Supplementary file 8 — Source Data Fig. 5 [file 44318_2024_71_MOESM8_ESM.zip › Fig 5/D/Figure_5D_NB_165_166.tif]

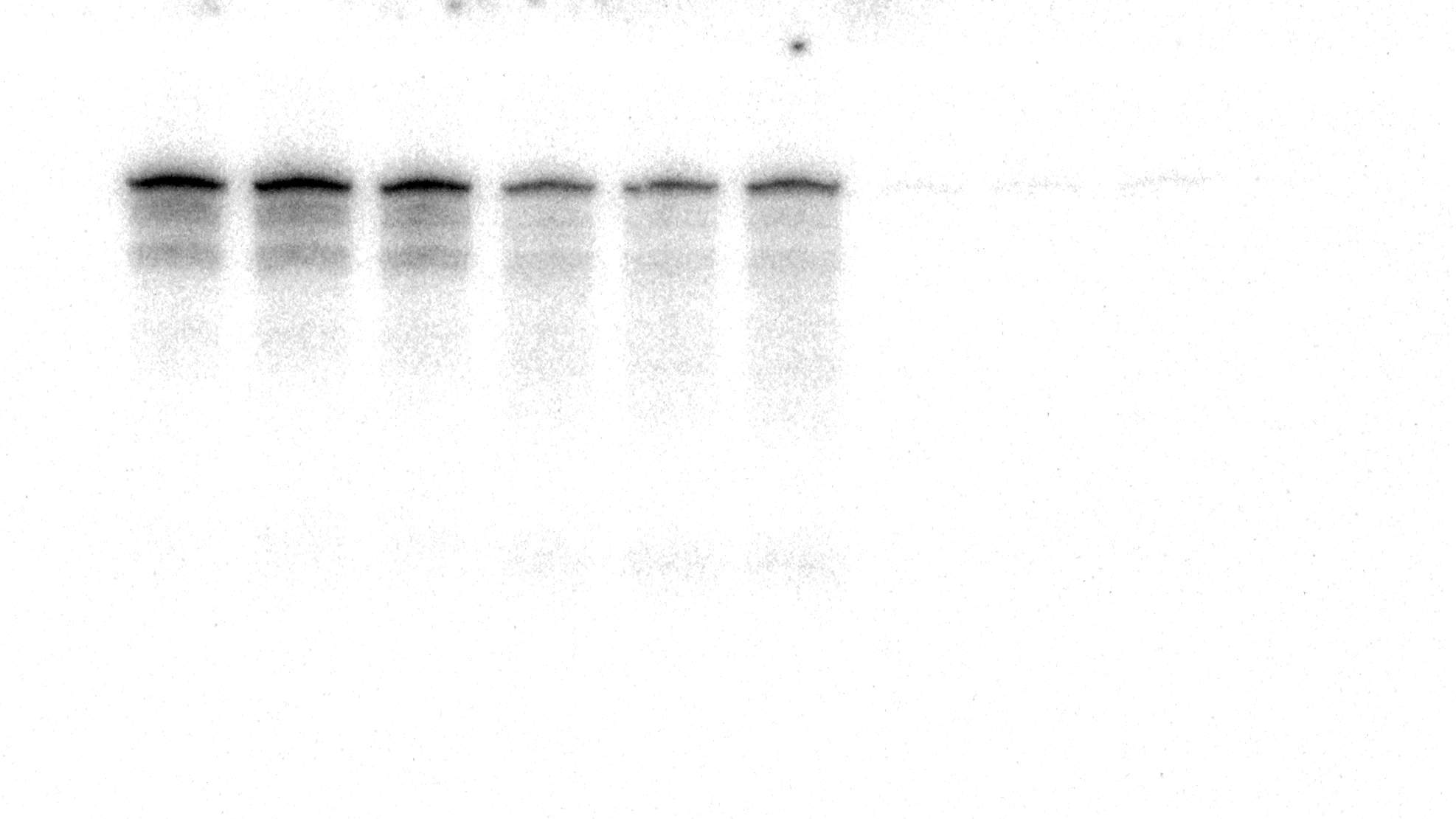

Supplement: Supplementary file 8 — Source Data Fig. 5 [file 44318_2024_71_MOESM8_ESM.zip › Fig 5/D/Figure_5D_NB_U6.tif]

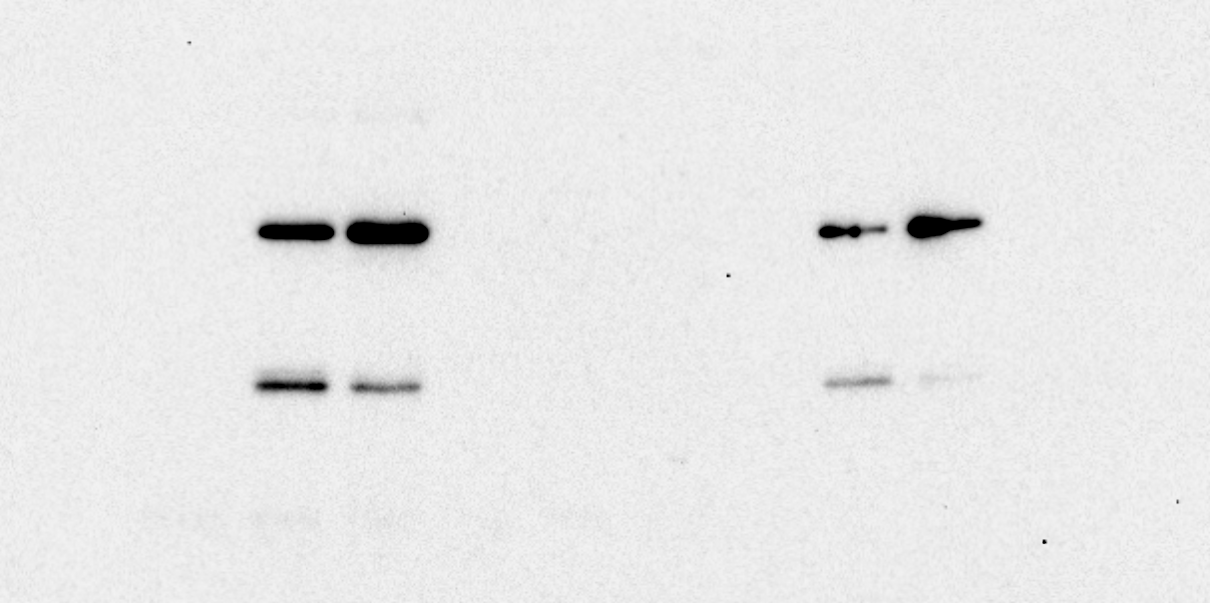

Supplement: Supplementary file 8 — Source Data Fig. 5 [file 44318_2024_71_MOESM8_ESM.zip › Fig 5/D/Figure_5D_WB_GFP.tif]

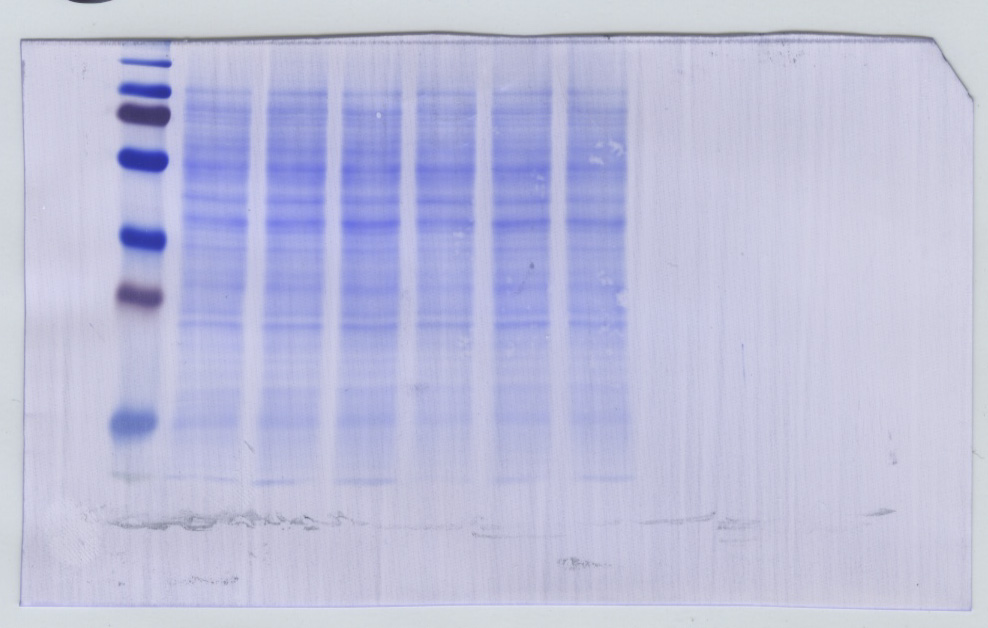

Supplement: Supplementary file 8 — Source Data Fig. 5 [file 44318_2024_71_MOESM8_ESM.zip › Fig 5/D/Figure_5D_coomassie_WB.jpg]

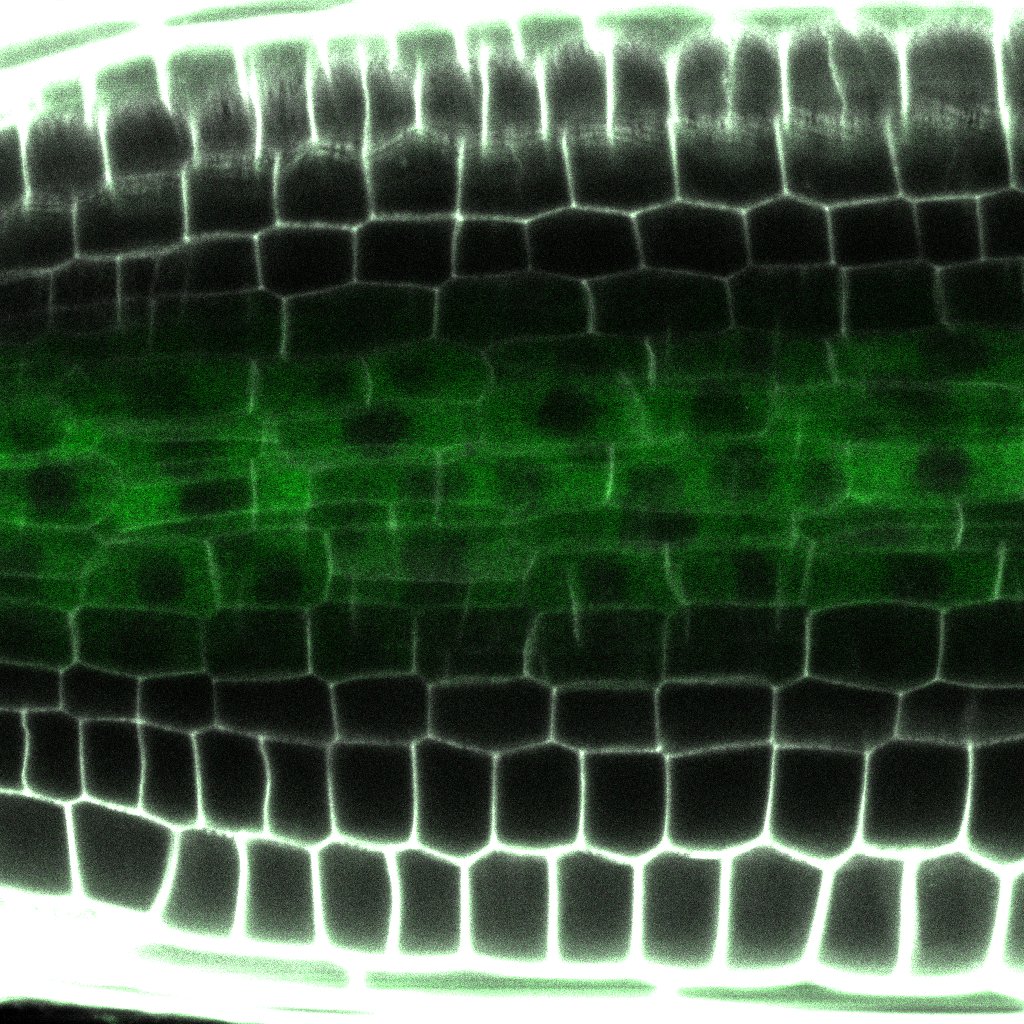

Supplement: Supplementary file 8 — Source Data Fig. 5 [file 44318_2024_71_MOESM8_ESM.zip › Fig 5/E/1.jpg]

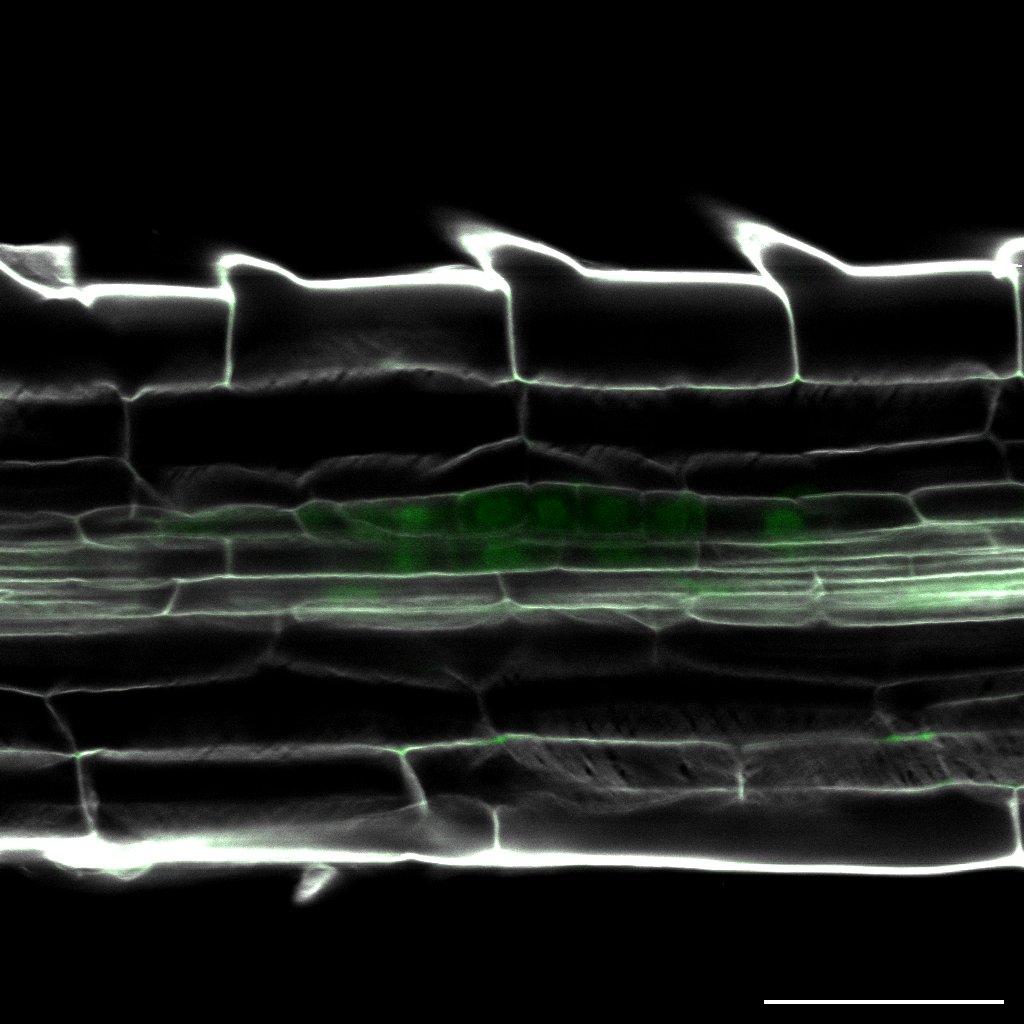

Supplement: Supplementary file 8 — Source Data Fig. 5 [file 44318_2024_71_MOESM8_ESM.zip › Fig 5/E/2.jpg]

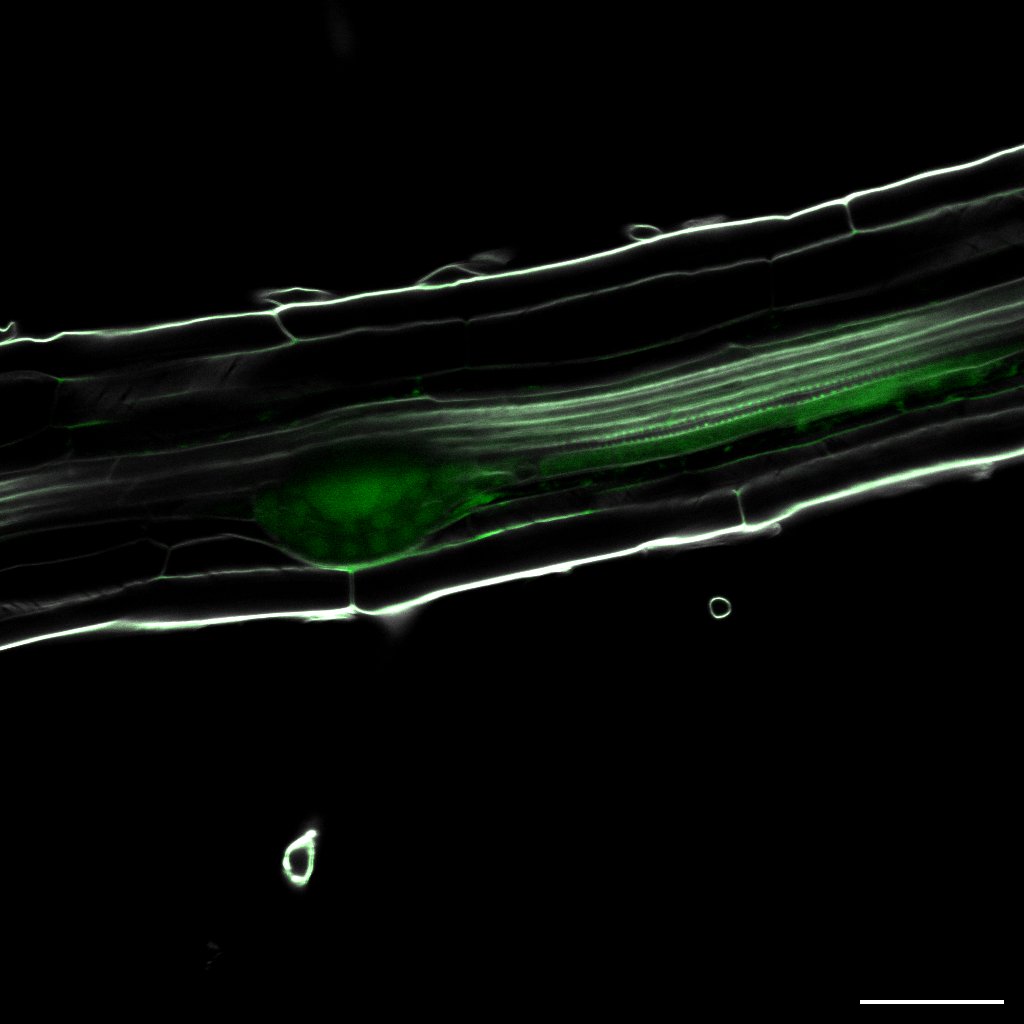

Supplement: Supplementary file 8 — Source Data Fig. 5 [file 44318_2024_71_MOESM8_ESM.zip › Fig 5/E/3.jpg]

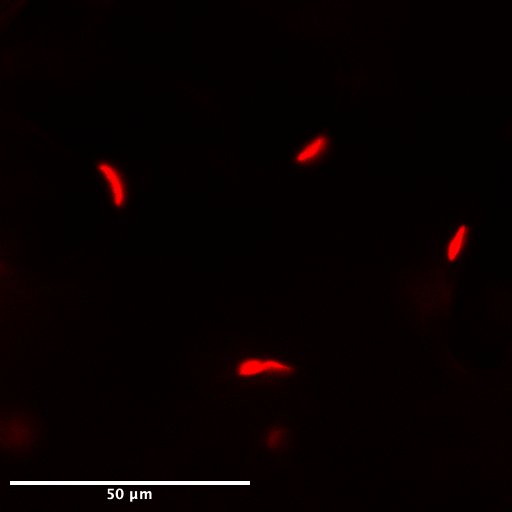

Supplement: Supplementary file 8 — Source Data Fig. 5 [file 44318_2024_71_MOESM8_ESM.zip › Fig 5/F/35SGFPAGO1/2.jpg]

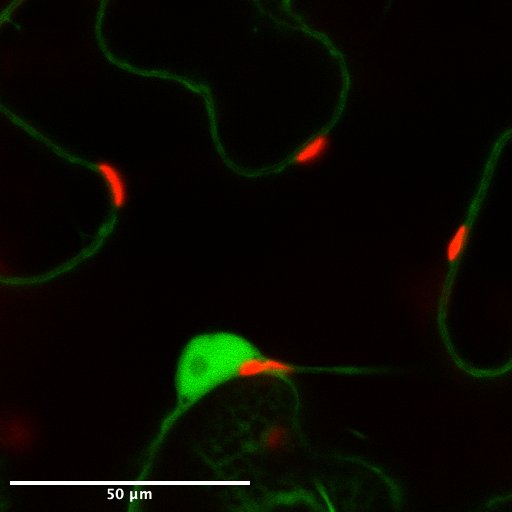

Supplement: Supplementary file 8 — Source Data Fig. 5 [file 44318_2024_71_MOESM8_ESM.zip › Fig 5/F/35SGFPAGO1/3.jpg]

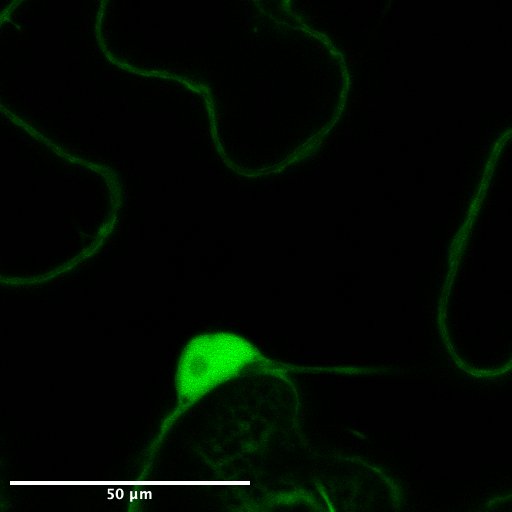

Supplement: Supplementary file 8 — Source Data Fig. 5 [file 44318_2024_71_MOESM8_ESM.zip › Fig 5/F/35SGFPAGO1/1.jpg]

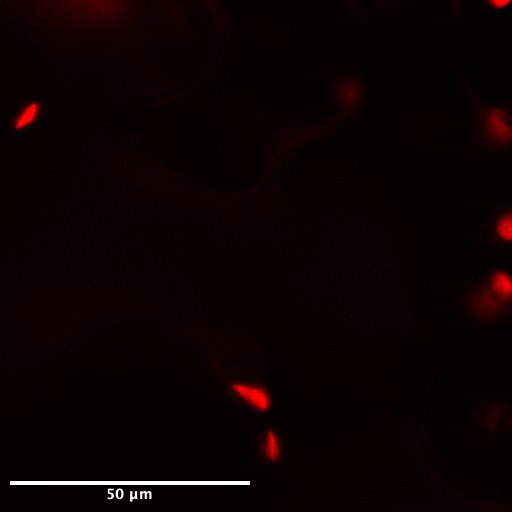

Supplement: Supplementary file 8 — Source Data Fig. 5 [file 44318_2024_71_MOESM8_ESM.zip › Fig 5/F/35SeGFP/2.jpg]

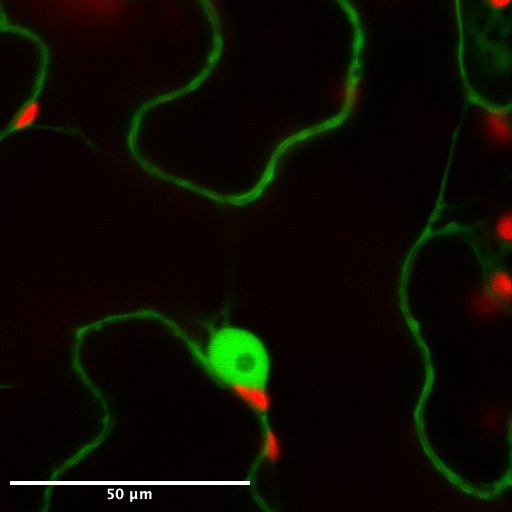

Supplement: Supplementary file 8 — Source Data Fig. 5 [file 44318_2024_71_MOESM8_ESM.zip › Fig 5/F/35SeGFP/3.jpg]

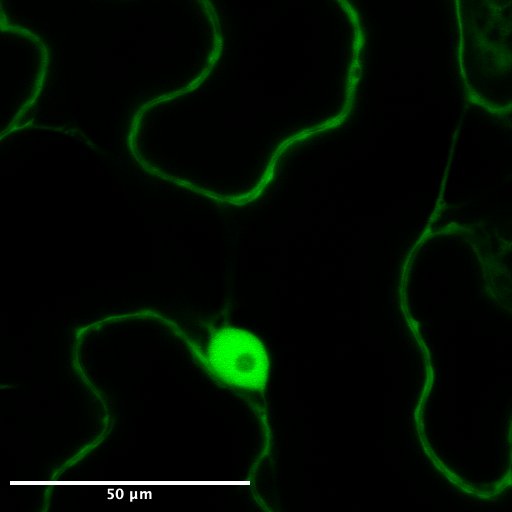

Supplement: Supplementary file 8 — Source Data Fig. 5 [file 44318_2024_71_MOESM8_ESM.zip › Fig 5/F/35SeGFP/1.jpg]

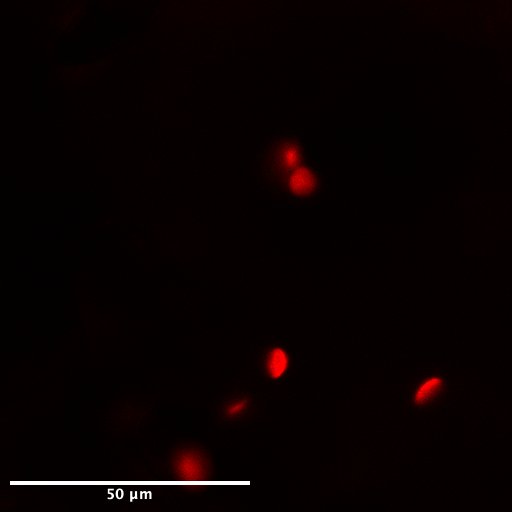

Supplement: Supplementary file 8 — Source Data Fig. 5 [file 44318_2024_71_MOESM8_ESM.zip › Fig 5/F/35SGFPAGO10/2.jpg]

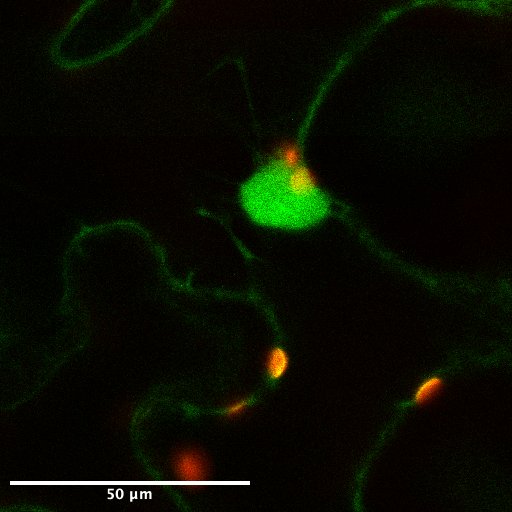

Supplement: Supplementary file 8 — Source Data Fig. 5 [file 44318_2024_71_MOESM8_ESM.zip › Fig 5/F/35SGFPAGO10/3.jpg]

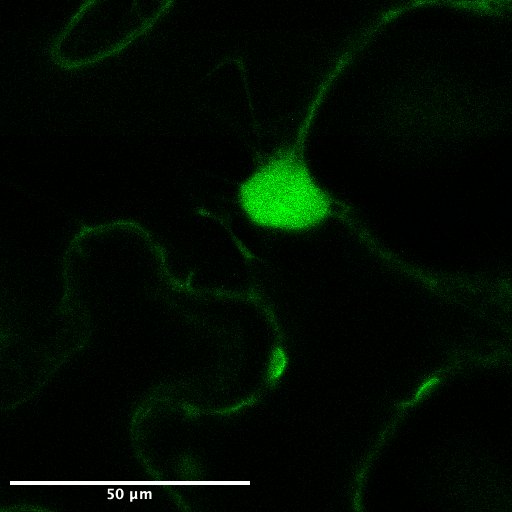

Supplement: Supplementary file 8 — Source Data Fig. 5 [file 44318_2024_71_MOESM8_ESM.zip › Fig 5/F/35SGFPAGO10/1.jpg]

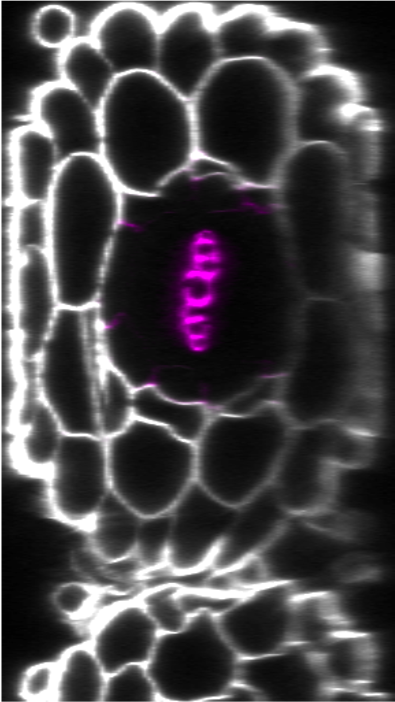

Supplement: Supplementary file 8 — Source Data Fig. 5 [file 44318_2024_71_MOESM8_ESM.zip › Fig 5/B/lower panel/SCR 1.png]

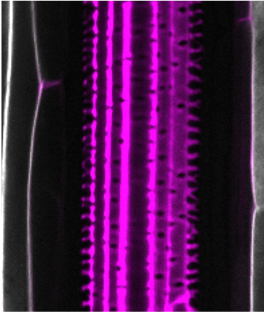

Supplement: Supplementary file 8 — Source Data Fig. 5 [file 44318_2024_71_MOESM8_ESM.zip › Fig 5/B/lower panel/SCR.png]

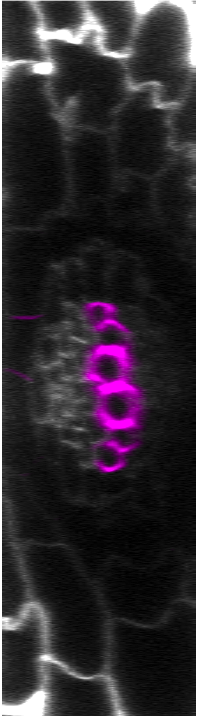

Supplement: Supplementary file 8 — Source Data Fig. 5 [file 44318_2024_71_MOESM8_ESM.zip › Fig 5/B/lower panel/SHR 1.png]

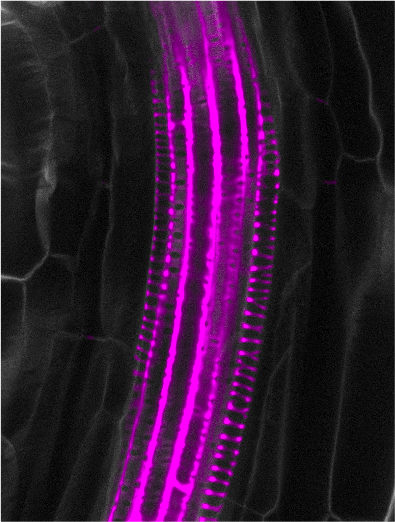

Supplement: Supplementary file 8 — Source Data Fig. 5 [file 44318_2024_71_MOESM8_ESM.zip › Fig 5/B/lower panel/SHR.png]

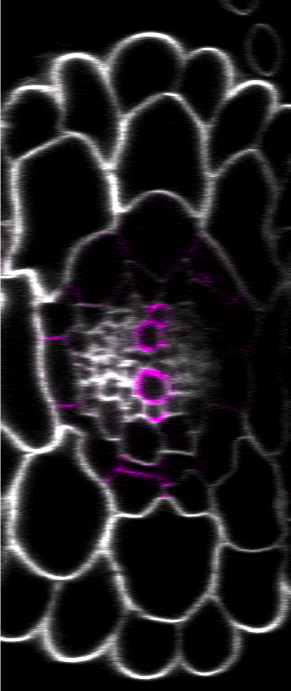

Supplement: Supplementary file 8 — Source Data Fig. 5 [file 44318_2024_71_MOESM8_ESM.zip › Fig 5/B/lower panel/WER 1.png]

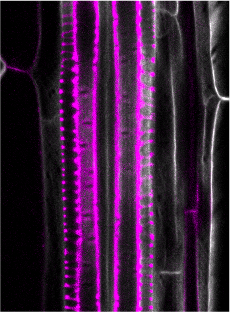

Supplement: Supplementary file 8 — Source Data Fig. 5 [file 44318_2024_71_MOESM8_ESM.zip › Fig 5/B/lower panel/WER.png]

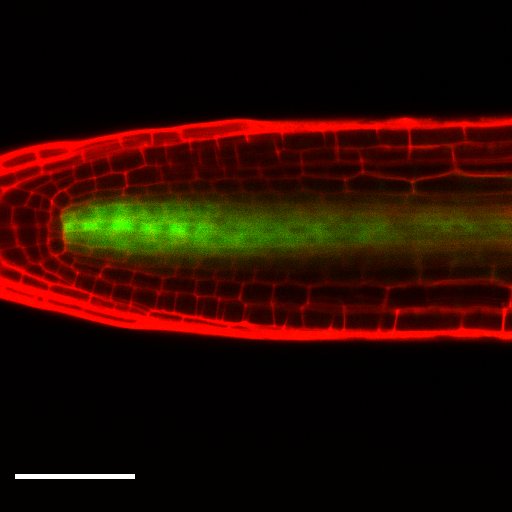

Supplement: Supplementary file 8 — Source Data Fig. 5 [file 44318_2024_71_MOESM8_ESM.zip › Fig 5/B/upper panel/SHRGFPp19.jpg]

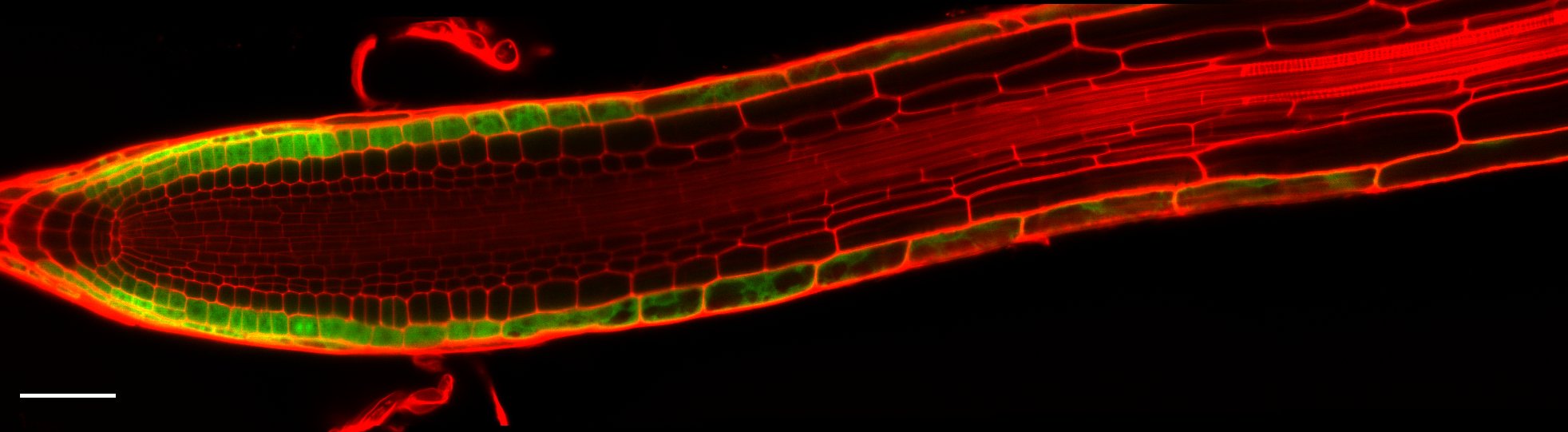

Supplement: Supplementary file 8 — Source Data Fig. 5 [file 44318_2024_71_MOESM8_ESM.zip › Fig 5/B/upper panel/WERGFPp19.jpg]

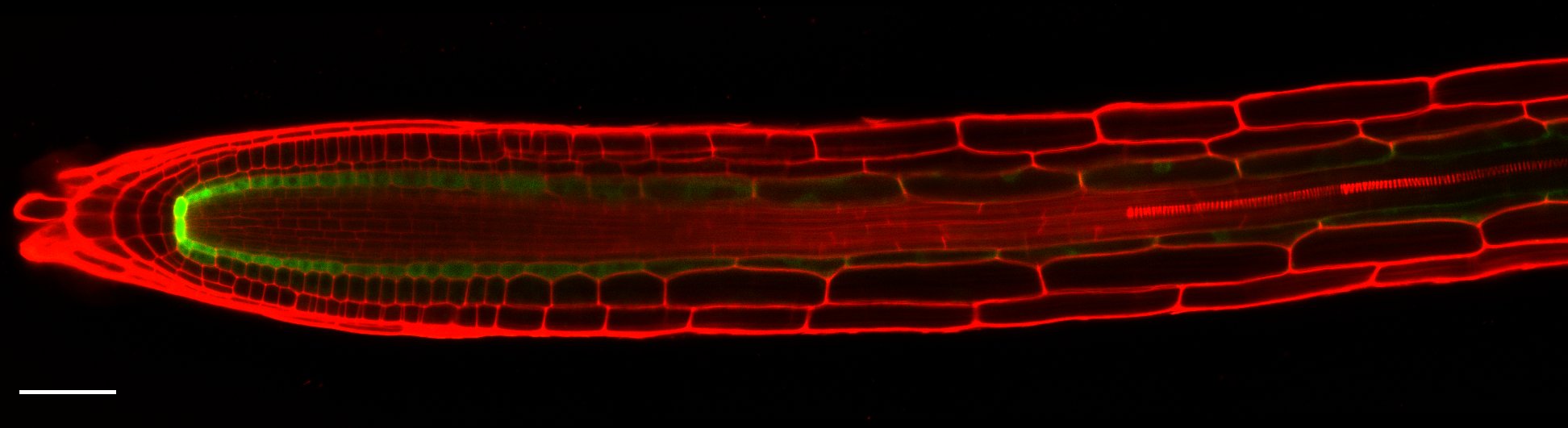

Supplement: Supplementary file 8 — Source Data Fig. 5 [file 44318_2024_71_MOESM8_ESM.zip › Fig 5/B/upper panel/SCRGFPp19.jpg]

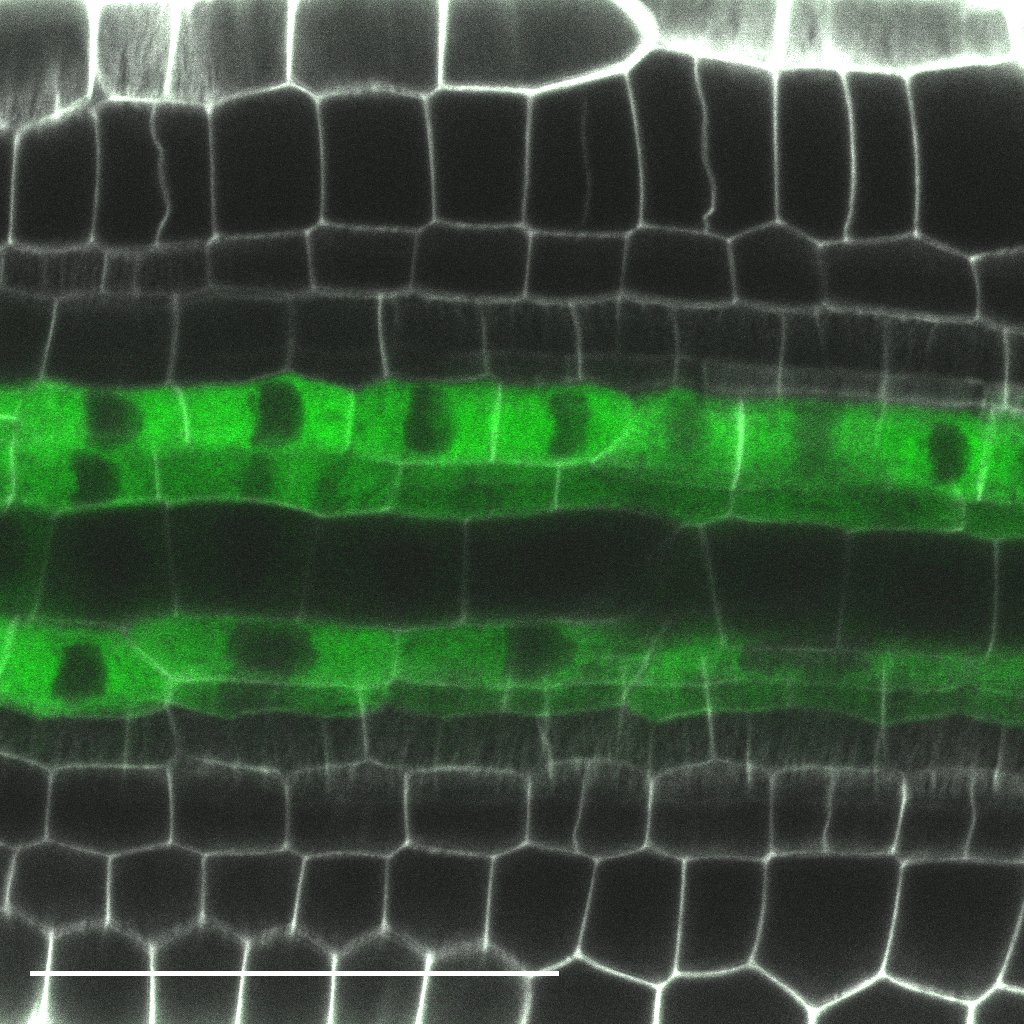

Supplement: Supplementary file 9 — Source Data Fig. 6 [file 44318_2024_71_MOESM9_ESM.zip › Fig 6/B/2.jpg]

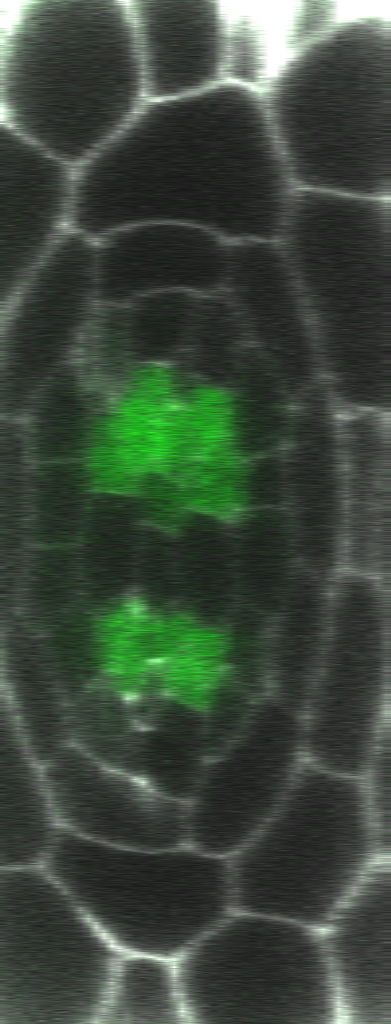

Supplement: Supplementary file 9 — Source Data Fig. 6 [file 44318_2024_71_MOESM9_ESM.zip › Fig 6/B/3.tif]

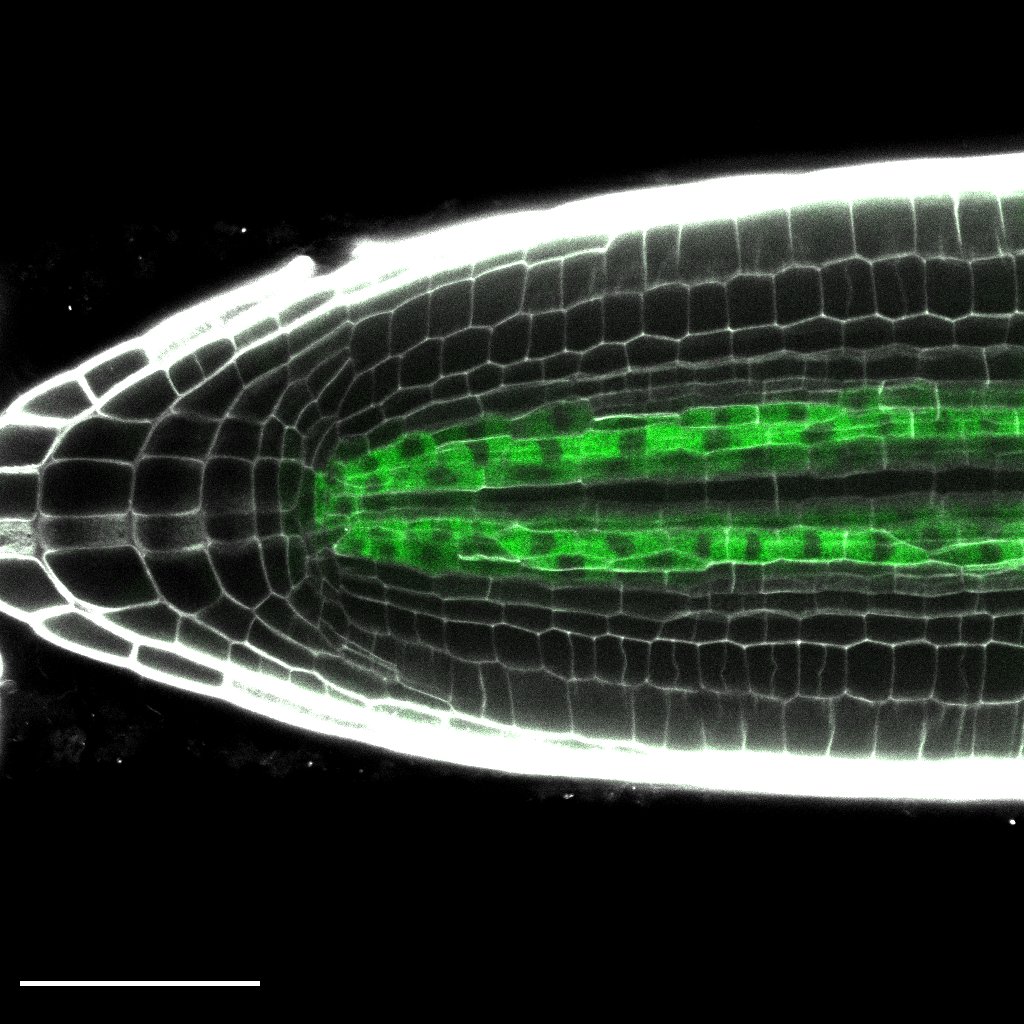

Supplement: Supplementary file 9 — Source Data Fig. 6 [file 44318_2024_71_MOESM9_ESM.zip › Fig 6/B/1.jpg]

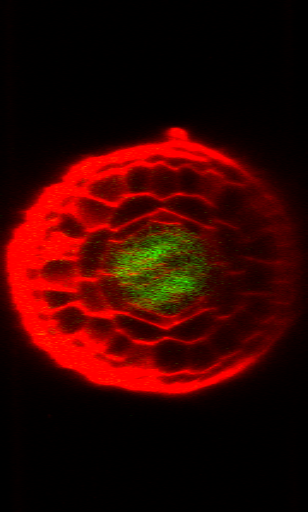

Supplement: Supplementary file 9 — Source Data Fig. 6 [file 44318_2024_71_MOESM9_ESM.zip › Fig 6/D/A10 NX-CS.tif]

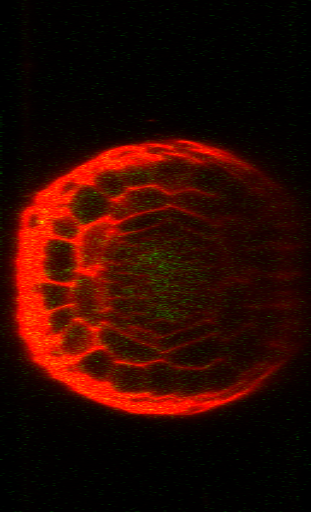

Supplement: Supplementary file 9 — Source Data Fig. 6 [file 44318_2024_71_MOESM9_ESM.zip › Fig 6/D/PHBPHBGFP in ago10-1-CS.tif]

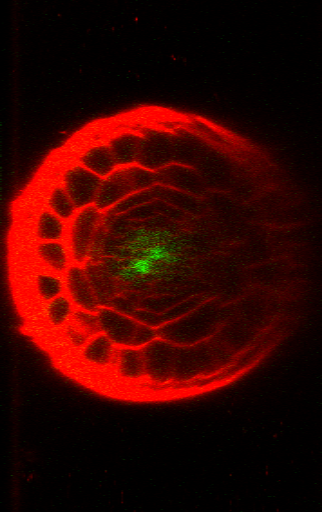

Supplement: Supplementary file 9 — Source Data Fig. 6 [file 44318_2024_71_MOESM9_ESM.zip › Fig 6/D/PHBPHBGFP-CS.tif]

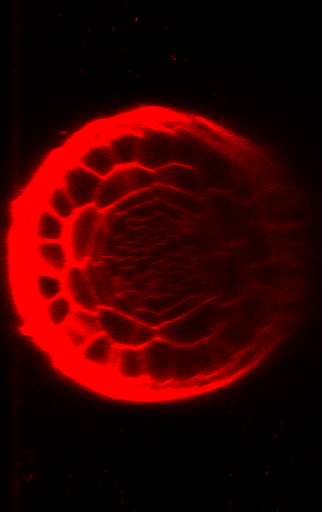

Supplement: Supplementary file 9 — Source Data Fig. 6 [file 44318_2024_71_MOESM9_ESM.zip › Fig 6/D/PHBPHBGFP-CS-minus GFP signal.tif]

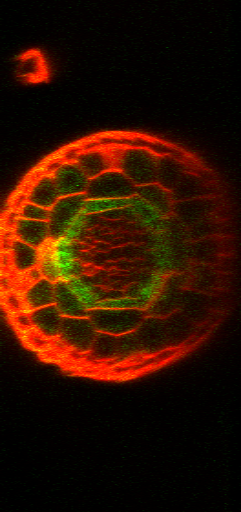

Supplement: Supplementary file 9 — Source Data Fig. 6 [file 44318_2024_71_MOESM9_ESM.zip › Fig 6/D/pmiR166bGFP-CS.tif]

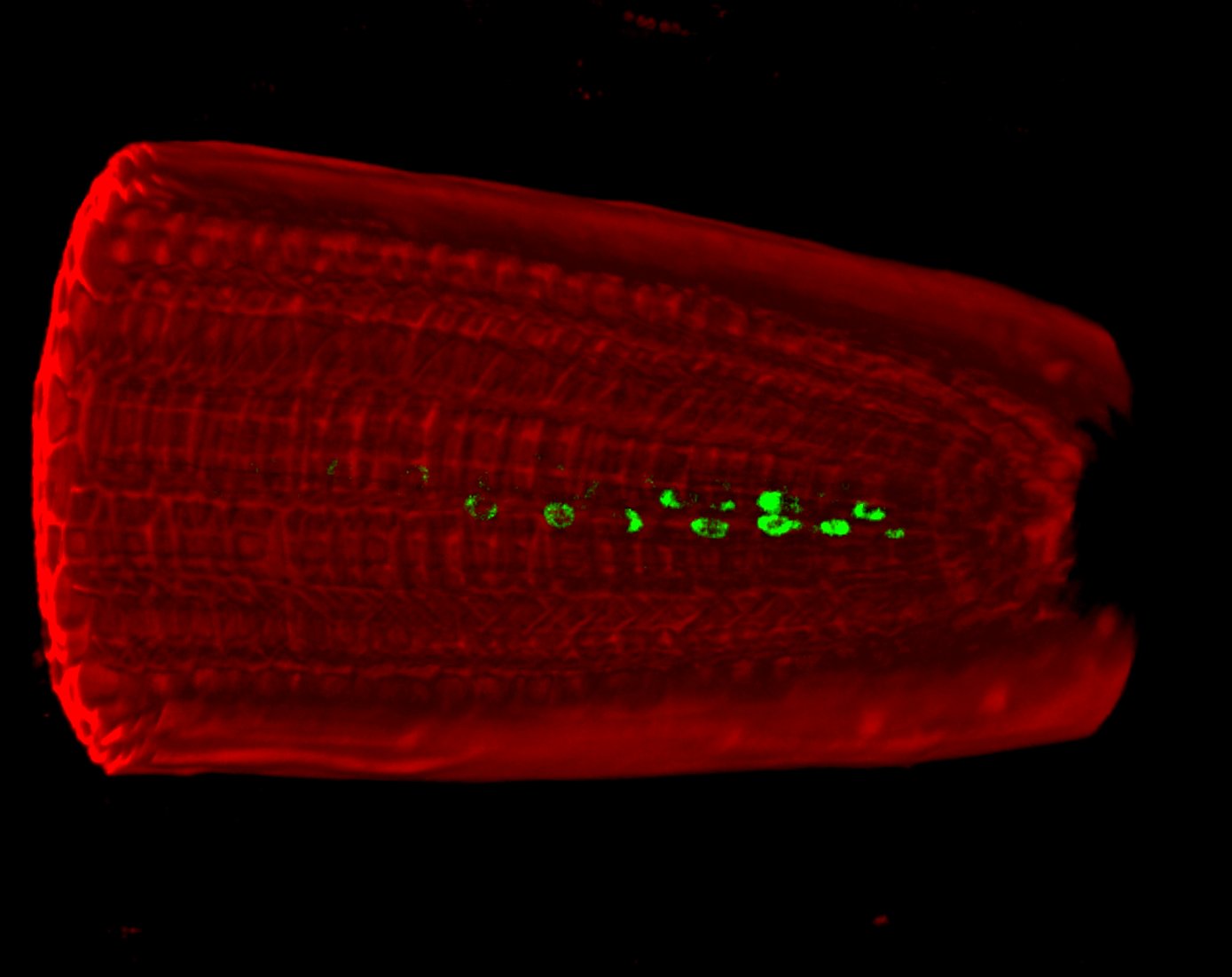

Supplement: Supplementary file 9 — Source Data Fig. 6 [file 44318_2024_71_MOESM9_ESM.zip › Fig 6/D/PHBPHBGFP 3D image.jpg]

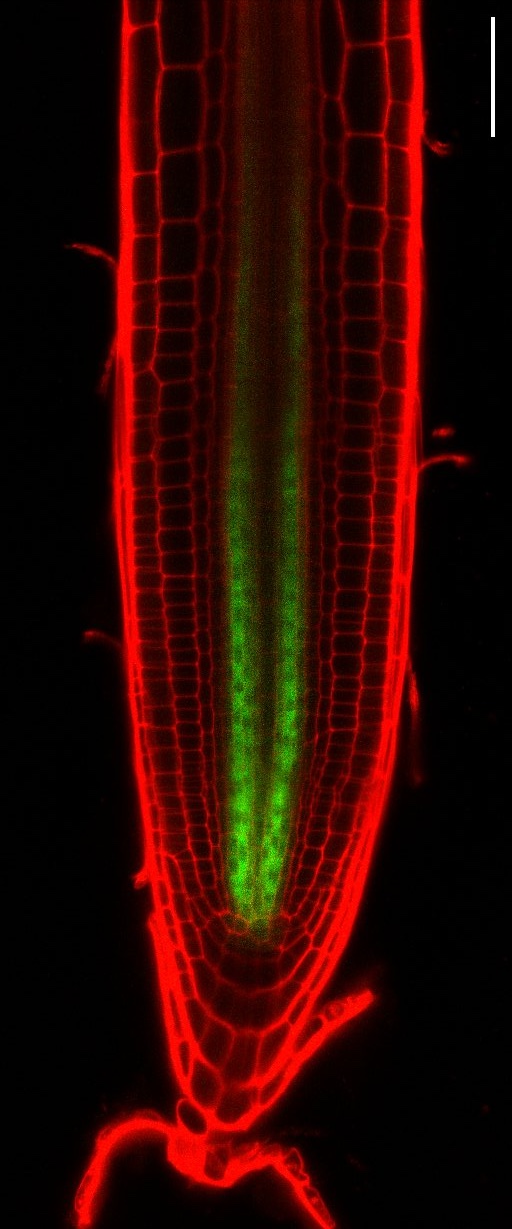

Supplement: Supplementary file 9 — Source Data Fig. 6 [file 44318_2024_71_MOESM9_ESM.zip › Fig 6/E/A10 NX.jpg]

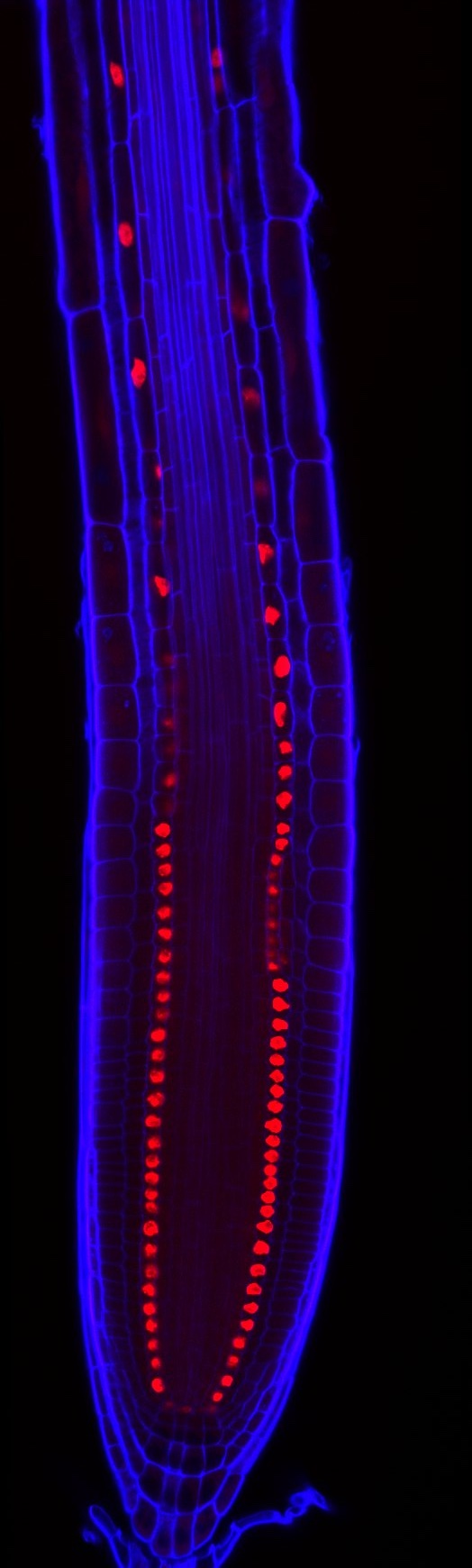

Supplement: Supplementary file 9 — Source Data Fig. 6 [file 44318_2024_71_MOESM9_ESM.zip › Fig 6/E/pmiR165amch.jpg]

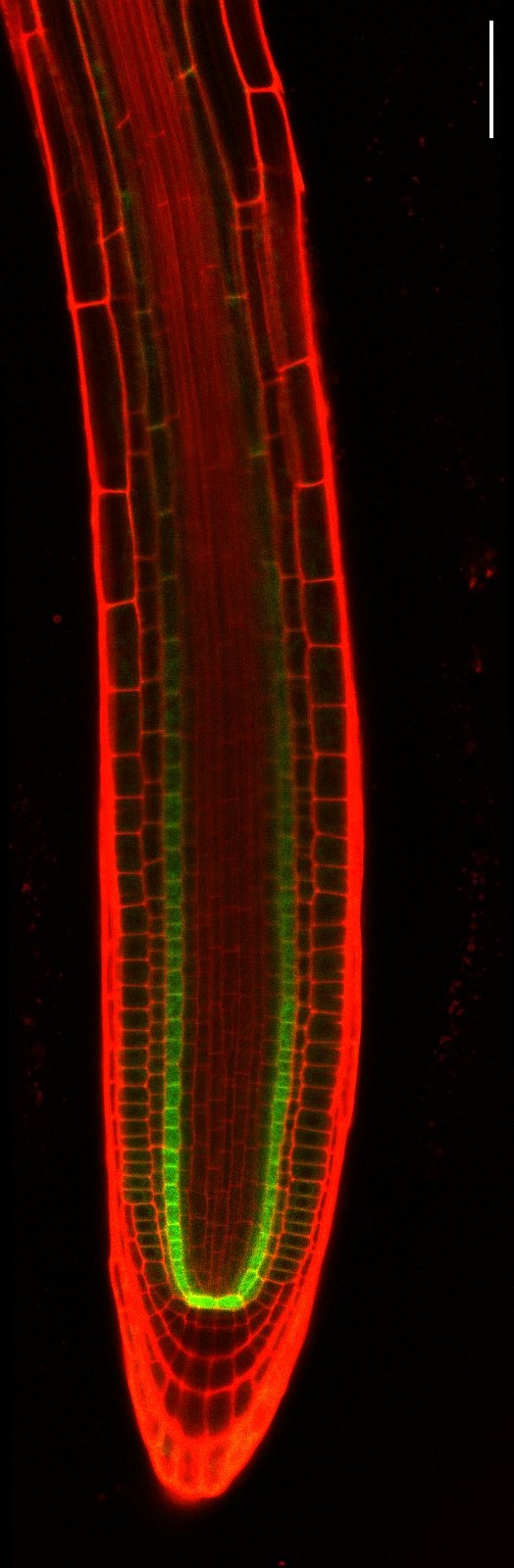

Supplement: Supplementary file 9 — Source Data Fig. 6 [file 44318_2024_71_MOESM9_ESM.zip › Fig 6/E/pmiR166bGFP.jpg]

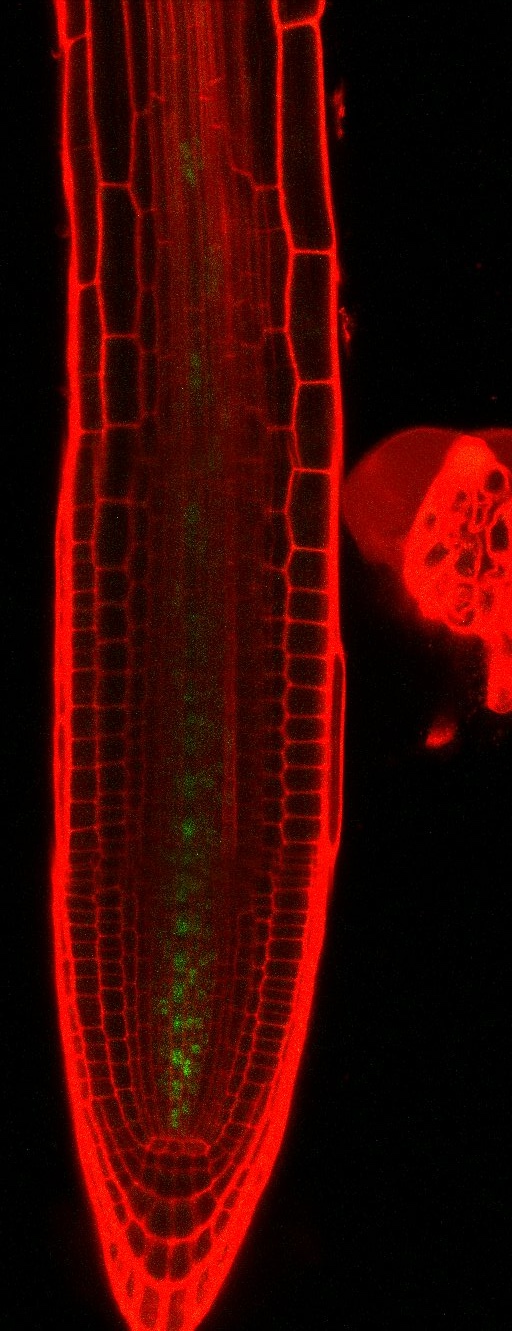

Supplement: Supplementary file 9 — Source Data Fig. 6 [file 44318_2024_71_MOESM9_ESM.zip › Fig 6/E/PHBPHBGFP.jpg]

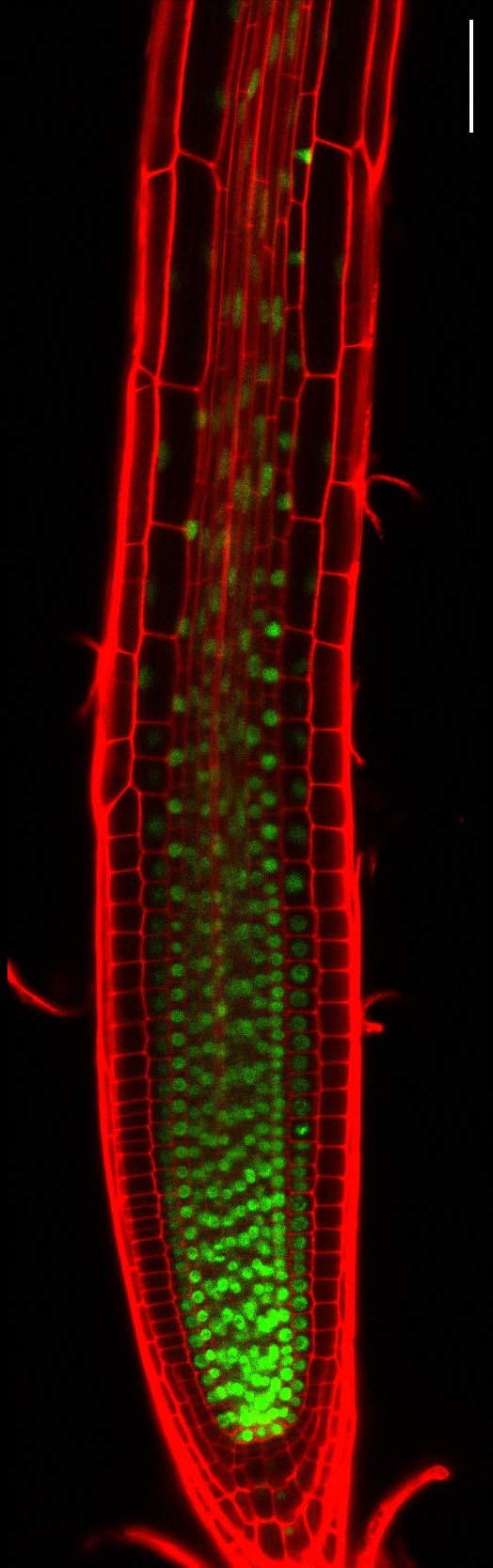

Supplement: Supplementary file 9 — Source Data Fig. 6 [file 44318_2024_71_MOESM9_ESM.zip › Fig 6/E/PHBH2BGFP.jpg]

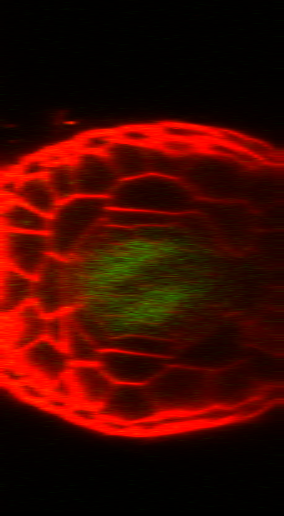

Supplement: Supplementary file 9 — Source Data Fig. 6 [file 44318_2024_71_MOESM9_ESM.zip › Fig 6/A/Day series of A10GFP/6 DAG-CS.tif]

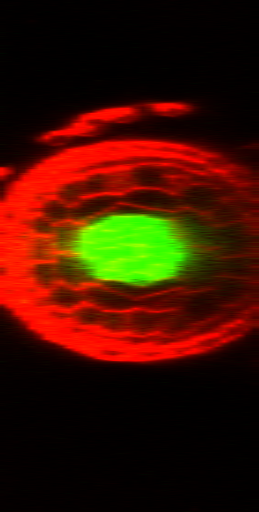

Supplement: Supplementary file 9 — Source Data Fig. 6 [file 44318_2024_71_MOESM9_ESM.zip › Fig 6/A/Day series of A10GFP/3 DAG-CS.tif]

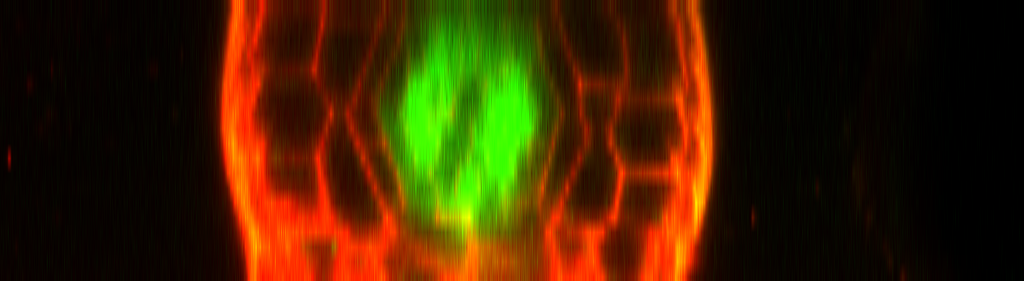

Supplement: Supplementary file 9 — Source Data Fig. 6 [file 44318_2024_71_MOESM9_ESM.zip › Fig 6/A/Day series of A10GFP/4 DAG-CS.tif]

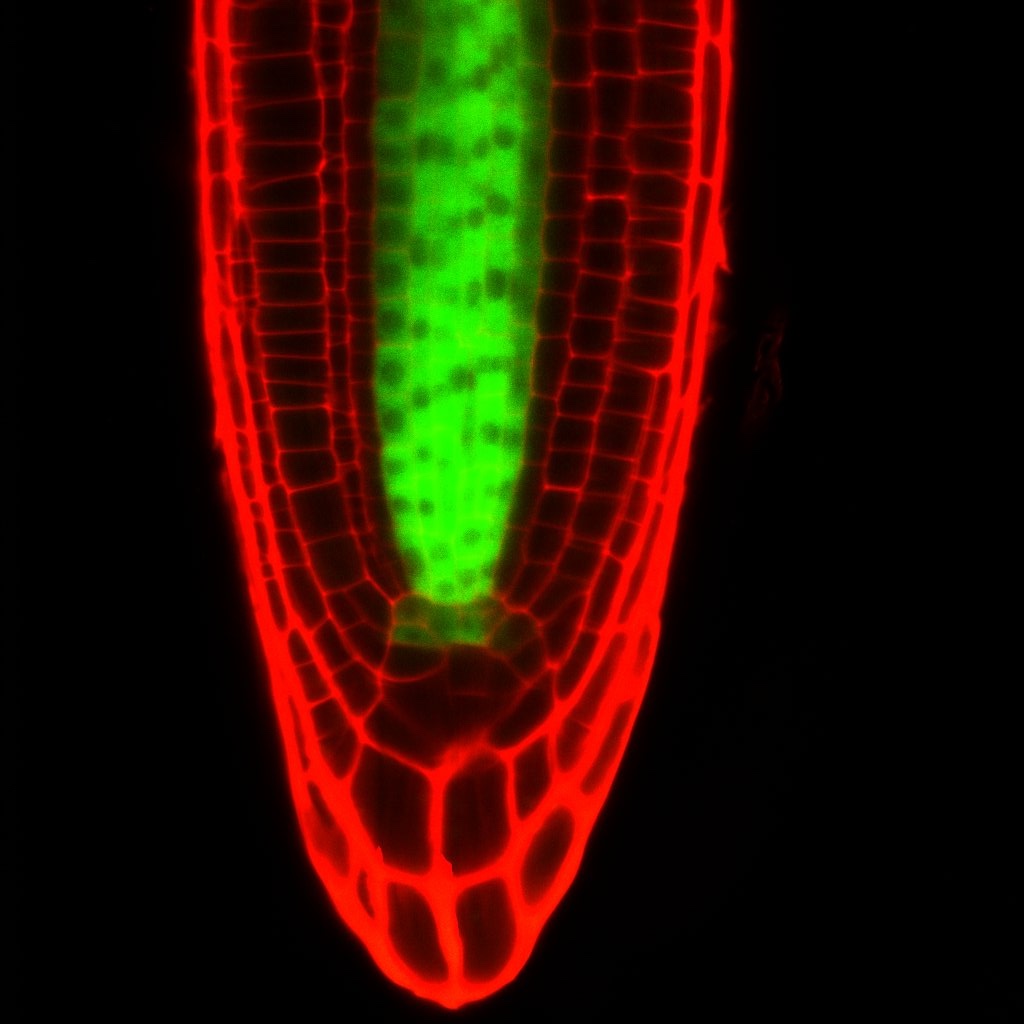

Supplement: Supplementary file 9 — Source Data Fig. 6 [file 44318_2024_71_MOESM9_ESM.zip › Fig 6/A/Day series of A10GFP/3 DAG.jpg]

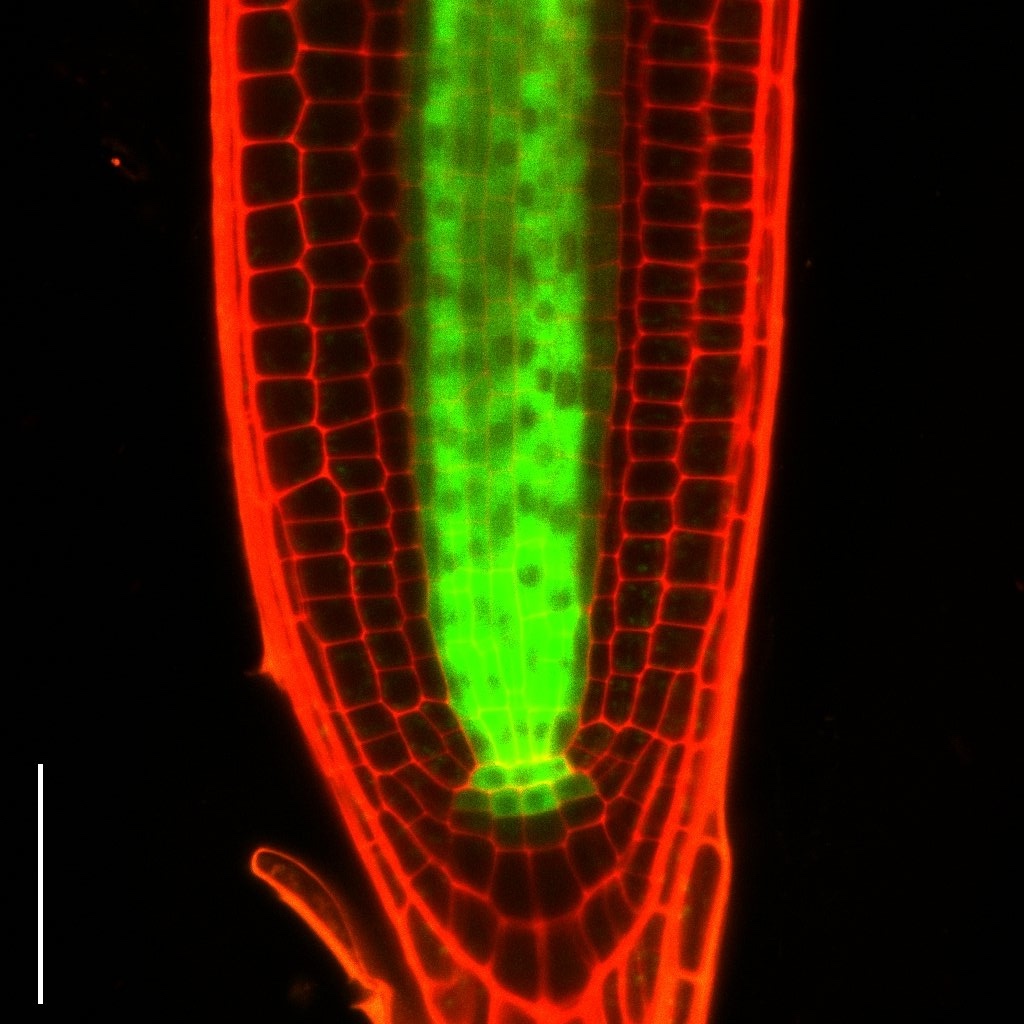

Supplement: Supplementary file 9 — Source Data Fig. 6 [file 44318_2024_71_MOESM9_ESM.zip › Fig 6/A/Day series of A10GFP/4 DAG.jpg]

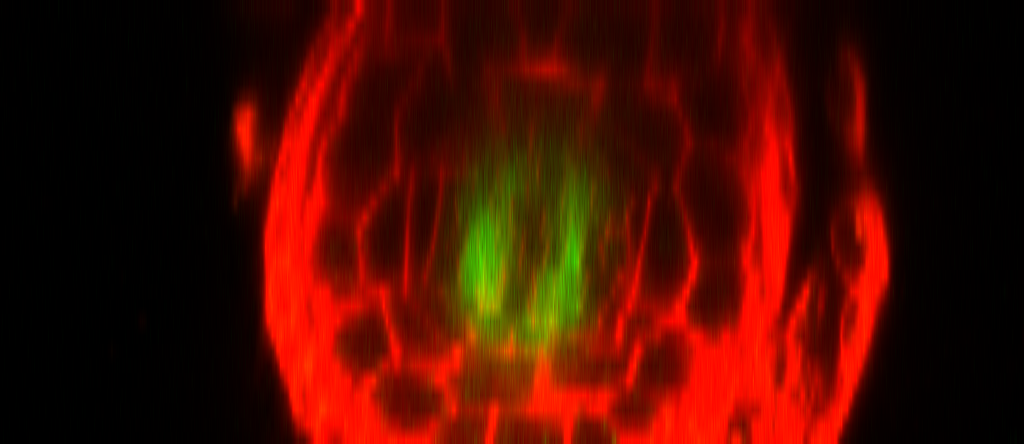

Supplement: Supplementary file 9 — Source Data Fig. 6 [file 44318_2024_71_MOESM9_ESM.zip › Fig 6/A/Day series of A10GFP/5 DAG-CS.tif]

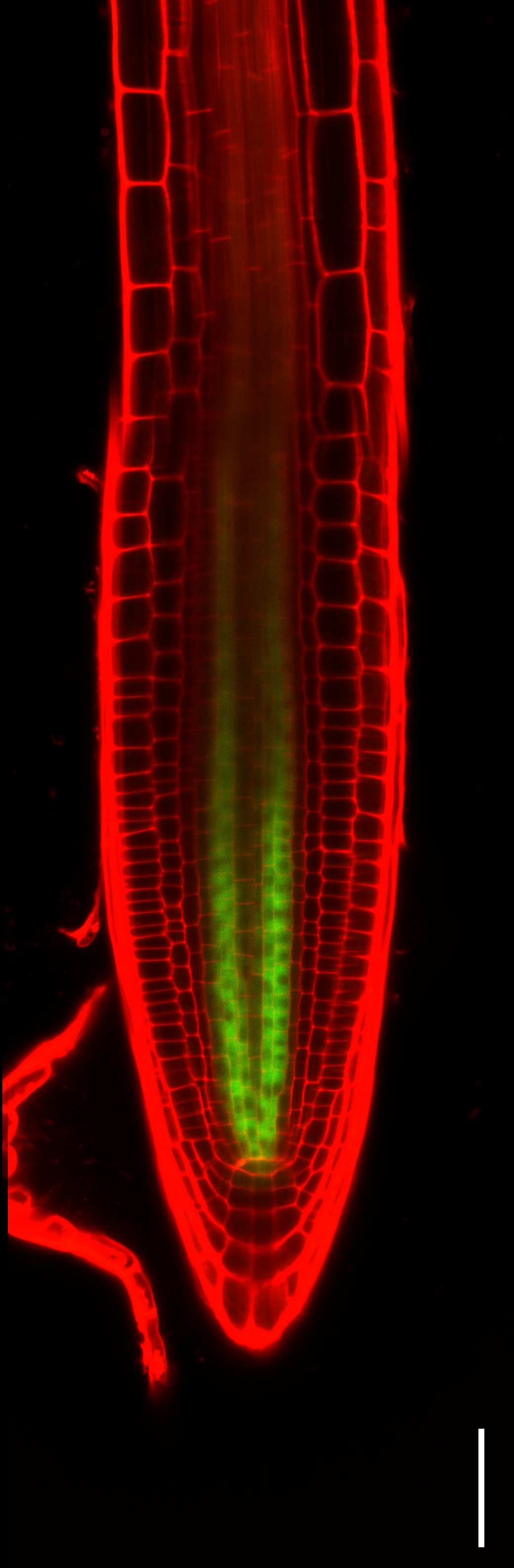

Supplement: Supplementary file 9 — Source Data Fig. 6 [file 44318_2024_71_MOESM9_ESM.zip › Fig 6/A/Day series of A10GFP/5 DAG.jpg]

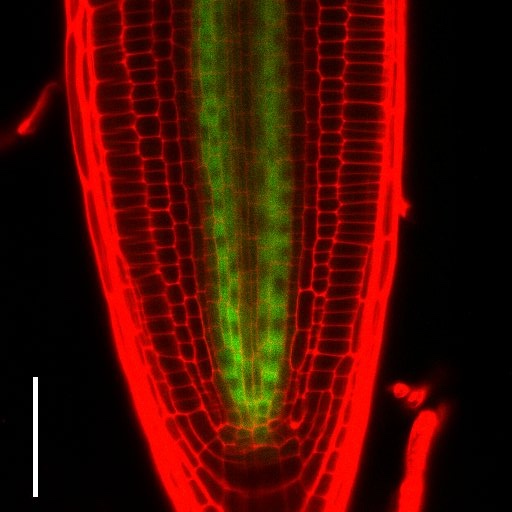

Supplement: Supplementary file 9 — Source Data Fig. 6 [file 44318_2024_71_MOESM9_ESM.zip › Fig 6/A/Day series of A10GFP/6 DAG.jpg]

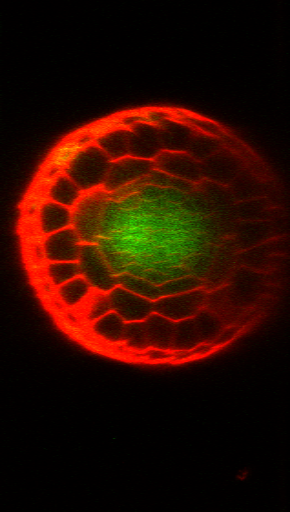

Supplement: Supplementary file 9 — Source Data Fig. 6 [file 44318_2024_71_MOESM9_ESM.zip › Fig 6/A/Day series of AGO10NTF/6 DAG-CS.tif]

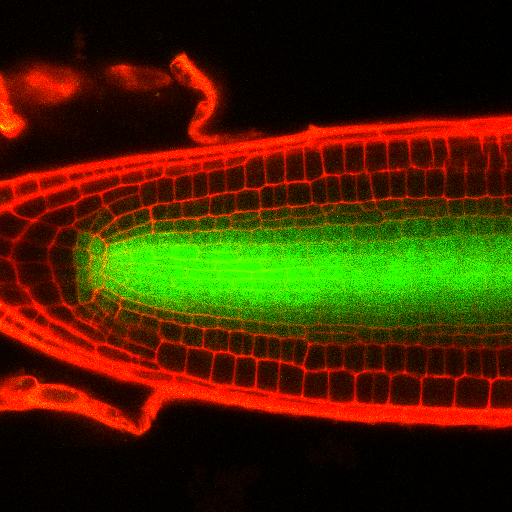

Supplement: Supplementary file 9 — Source Data Fig. 6 [file 44318_2024_71_MOESM9_ESM.zip › Fig 6/A/Day series of AGO10NTF/3 DAG.tif]

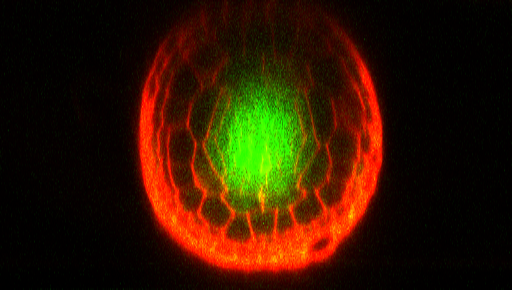

Supplement: Supplementary file 9 — Source Data Fig. 6 [file 44318_2024_71_MOESM9_ESM.zip › Fig 6/A/Day series of AGO10NTF/3 DAG-CS.tif]

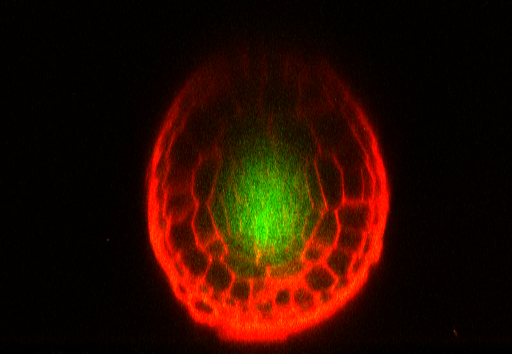

Supplement: Supplementary file 9 — Source Data Fig. 6 [file 44318_2024_71_MOESM9_ESM.zip › Fig 6/A/Day series of AGO10NTF/4 DAG-CS.tif]

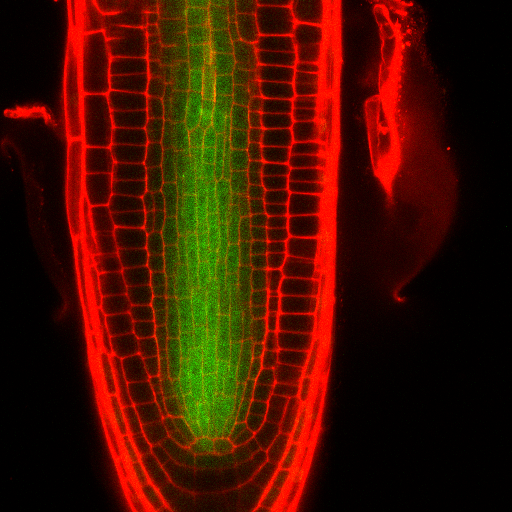

Supplement: Supplementary file 9 — Source Data Fig. 6 [file 44318_2024_71_MOESM9_ESM.zip › Fig 6/A/Day series of AGO10NTF/5 DAG.tif]

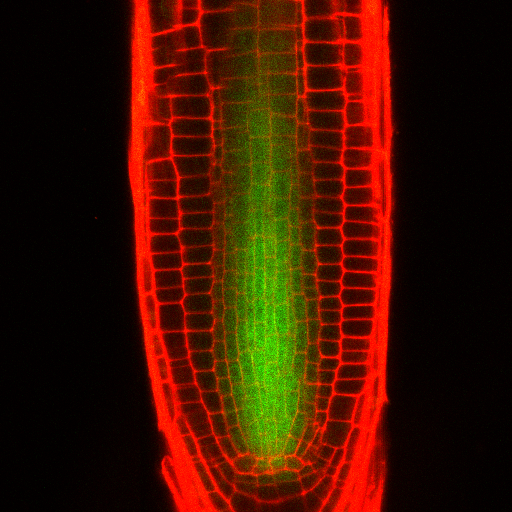

Supplement: Supplementary file 9 — Source Data Fig. 6 [file 44318_2024_71_MOESM9_ESM.zip › Fig 6/A/Day series of AGO10NTF/4 DAG.tif]

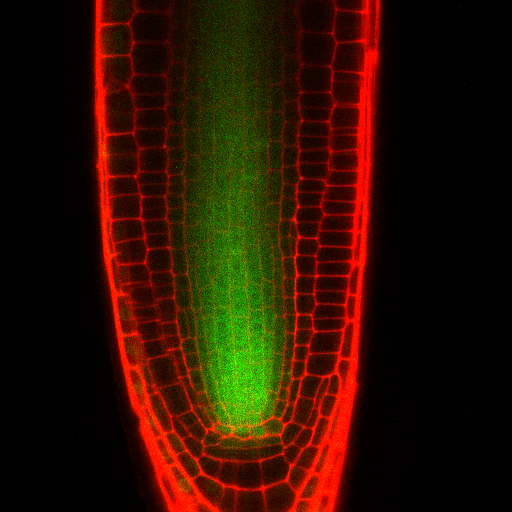

Supplement: Supplementary file 9 — Source Data Fig. 6 [file 44318_2024_71_MOESM9_ESM.zip › Fig 6/A/Day series of AGO10NTF/6 DAG.tif]

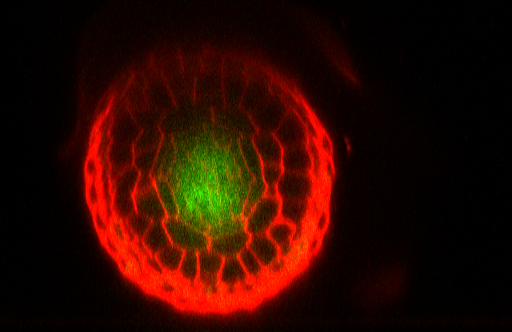

Supplement: Supplementary file 9 — Source Data Fig. 6 [file 44318_2024_71_MOESM9_ESM.zip › Fig 6/A/Day series of AGO10NTF/5 DAG-CS.tif]

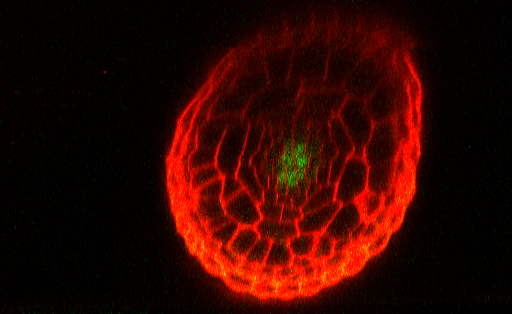

Supplement: Supplementary file 9 — Source Data Fig. 6 [file 44318_2024_71_MOESM9_ESM.zip › Fig 6/C/PHB PHB GFP/DAG3-CS.tif]

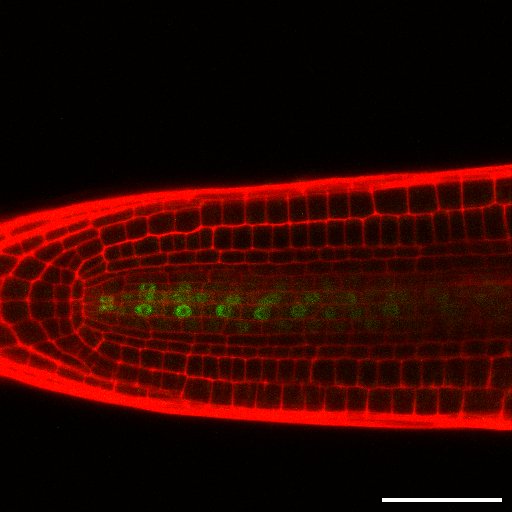

Supplement: Supplementary file 9 — Source Data Fig. 6 [file 44318_2024_71_MOESM9_ESM.zip › Fig 6/C/PHB PHB GFP/DAG3.jpg]

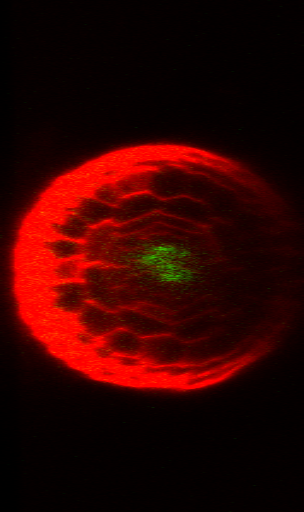

Supplement: Supplementary file 9 — Source Data Fig. 6 [file 44318_2024_71_MOESM9_ESM.zip › Fig 6/C/PHB PHB GFP/DAG4-CS.tif]

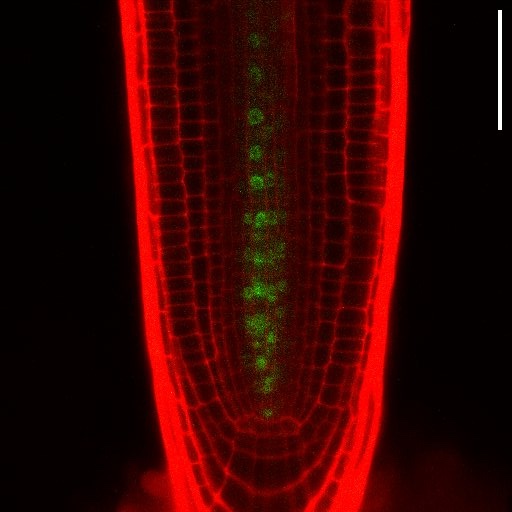

Supplement: Supplementary file 9 — Source Data Fig. 6 [file 44318_2024_71_MOESM9_ESM.zip › Fig 6/C/PHB PHB GFP/DAG4.jpg]

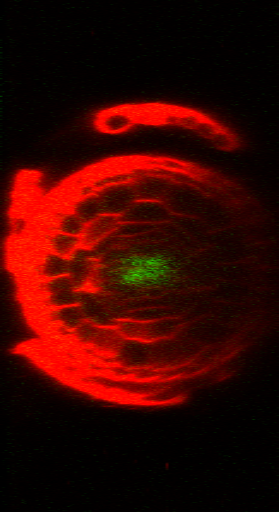

Supplement: Supplementary file 9 — Source Data Fig. 6 [file 44318_2024_71_MOESM9_ESM.zip › Fig 6/C/PHB PHB GFP/DAG5-CS.tif]

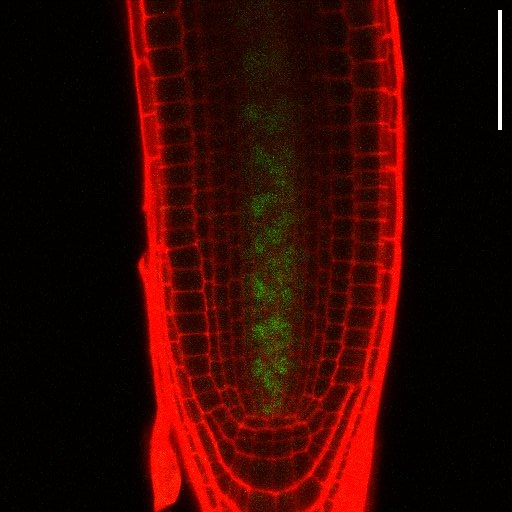

Supplement: Supplementary file 9 — Source Data Fig. 6 [file 44318_2024_71_MOESM9_ESM.zip › Fig 6/C/PHB PHB GFP/DAG5.jpg]

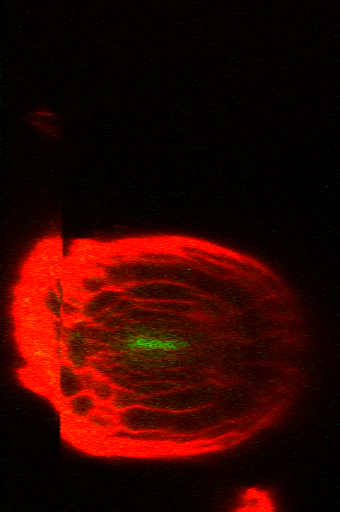

Supplement: Supplementary file 9 — Source Data Fig. 6 [file 44318_2024_71_MOESM9_ESM.zip › Fig 6/C/PHB PHB GFP/DAG6-CS.tif]

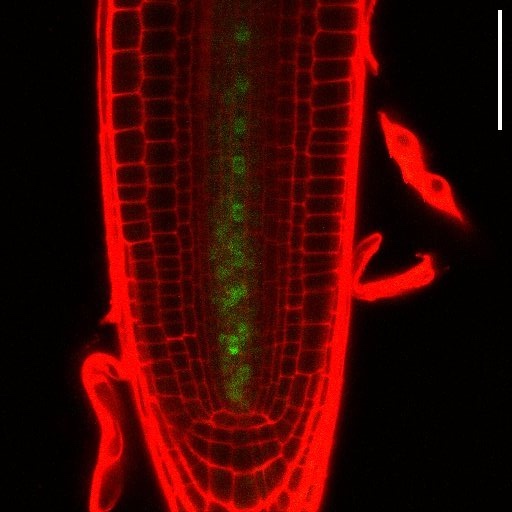

Supplement: Supplementary file 9 — Source Data Fig. 6 [file 44318_2024_71_MOESM9_ESM.zip › Fig 6/C/PHB PHB GFP/DAG6.jpg]

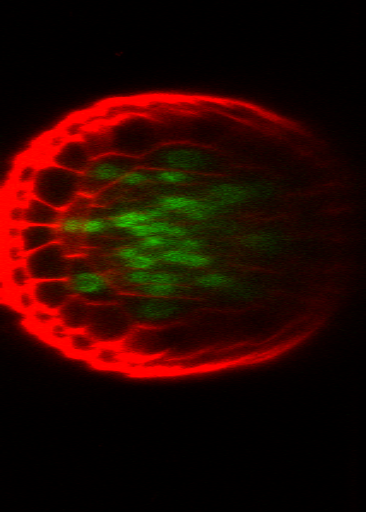

Supplement: Supplementary file 9 — Source Data Fig. 6 [file 44318_2024_71_MOESM9_ESM.zip › Fig 6/C/PHB H2B GFP/DAG3-CS.tif]

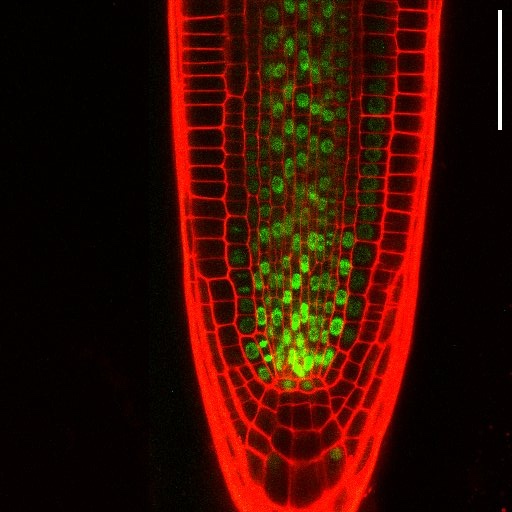

Supplement: Supplementary file 9 — Source Data Fig. 6 [file 44318_2024_71_MOESM9_ESM.zip › Fig 6/C/PHB H2B GFP/DAG3.jpg]

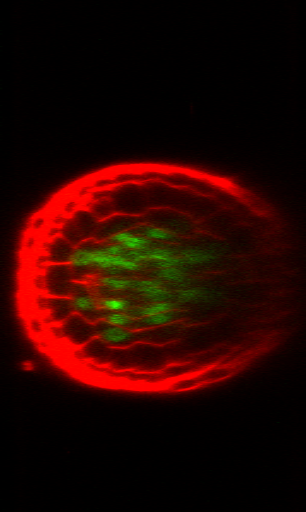

Supplement: Supplementary file 9 — Source Data Fig. 6 [file 44318_2024_71_MOESM9_ESM.zip › Fig 6/C/PHB H2B GFP/DAG4-CS.tif]

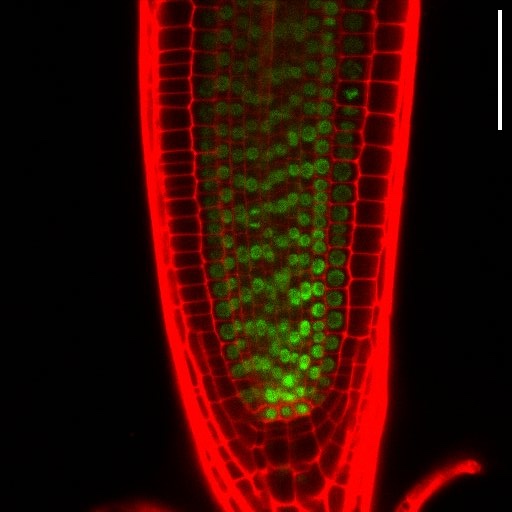

Supplement: Supplementary file 9 — Source Data Fig. 6 [file 44318_2024_71_MOESM9_ESM.zip › Fig 6/C/PHB H2B GFP/DAG4.jpg]

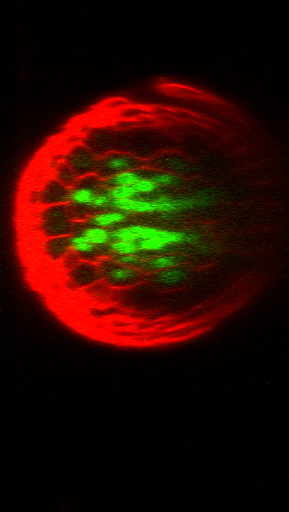

Supplement: Supplementary file 9 — Source Data Fig. 6 [file 44318_2024_71_MOESM9_ESM.zip › Fig 6/C/PHB H2B GFP/DAG5-CS.tif]

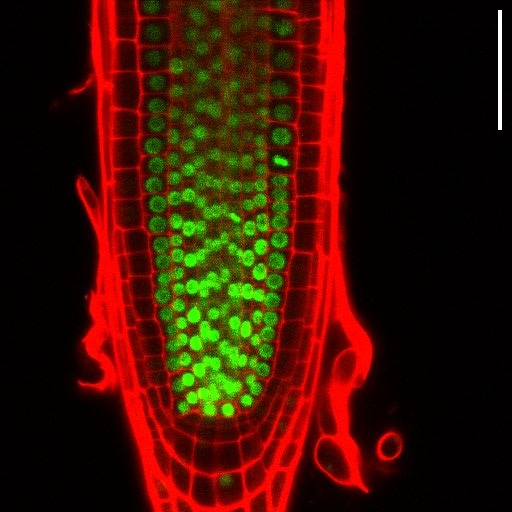

Supplement: Supplementary file 9 — Source Data Fig. 6 [file 44318_2024_71_MOESM9_ESM.zip › Fig 6/C/PHB H2B GFP/DAG5.jpg]

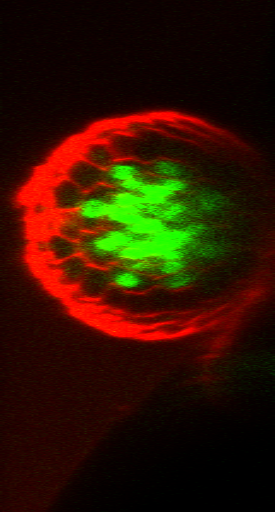

Supplement: Supplementary file 9 — Source Data Fig. 6 [file 44318_2024_71_MOESM9_ESM.zip › Fig 6/C/PHB H2B GFP/DAG6-CS.tif]

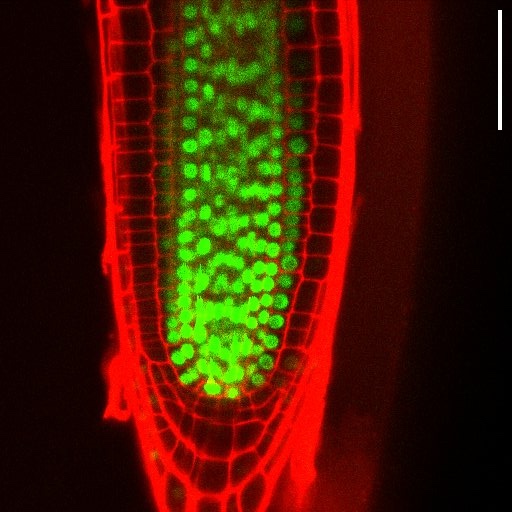

Supplement: Supplementary file 9 — Source Data Fig. 6 [file 44318_2024_71_MOESM9_ESM.zip › Fig 6/C/PHB H2B GFP/DAG6.jpg]

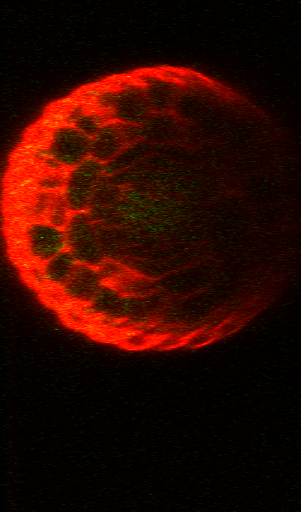

Supplement: Supplementary file 9 — Source Data Fig. 6 [file 44318_2024_71_MOESM9_ESM.zip › Fig 6/C/PHB PHB GFP in ago10-1/DAG3-CS.tif]

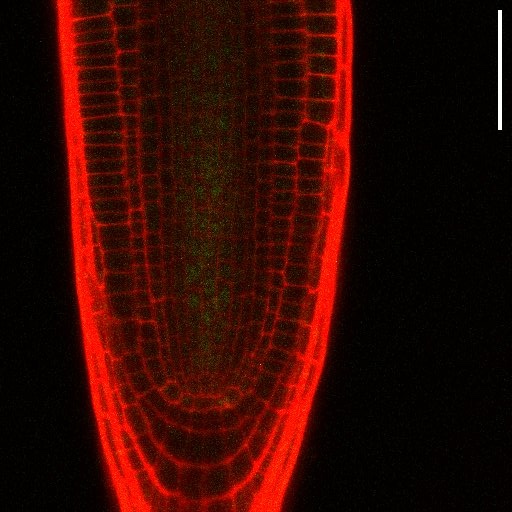

Supplement: Supplementary file 9 — Source Data Fig. 6 [file 44318_2024_71_MOESM9_ESM.zip › Fig 6/C/PHB PHB GFP in ago10-1/DAG3.jpg]

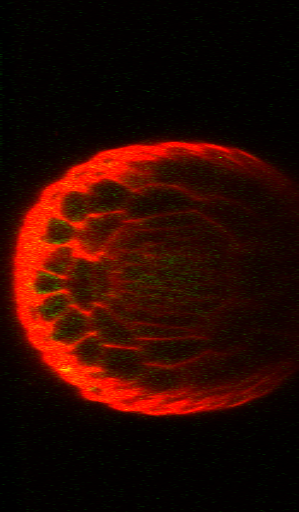

Supplement: Supplementary file 9 — Source Data Fig. 6 [file 44318_2024_71_MOESM9_ESM.zip › Fig 6/C/PHB PHB GFP in ago10-1/DAG4-CS.tif]

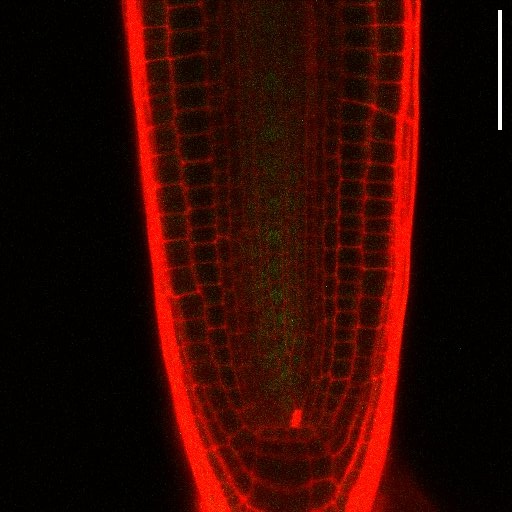

Supplement: Supplementary file 9 — Source Data Fig. 6 [file 44318_2024_71_MOESM9_ESM.zip › Fig 6/C/PHB PHB GFP in ago10-1/DAG4.jpg]

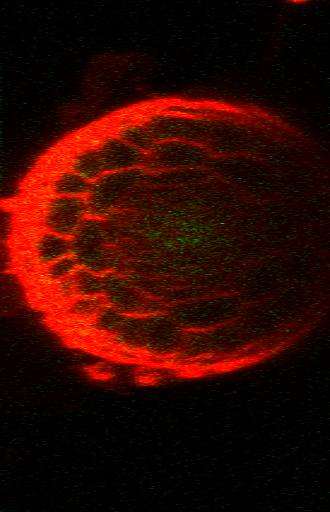

Supplement: Supplementary file 9 — Source Data Fig. 6 [file 44318_2024_71_MOESM9_ESM.zip › Fig 6/C/PHB PHB GFP in ago10-1/DAG5-CS.tif]

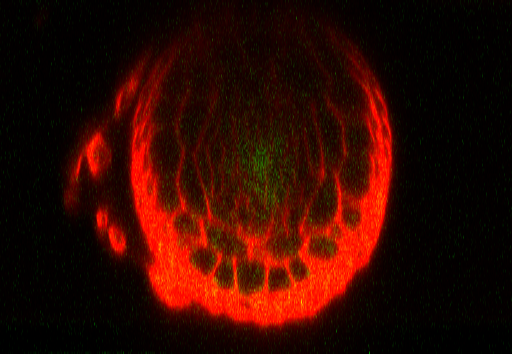

Supplement: Supplementary file 9 — Source Data Fig. 6 [file 44318_2024_71_MOESM9_ESM.zip › Fig 6/C/PHB PHB GFP in ago10-1/DAG6-CS.tif]

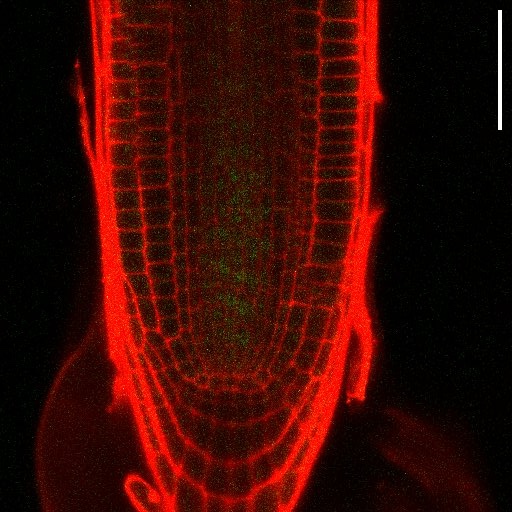

Supplement: Supplementary file 9 — Source Data Fig. 6 [file 44318_2024_71_MOESM9_ESM.zip › Fig 6/C/PHB PHB GFP in ago10-1/DAG5.jpg]

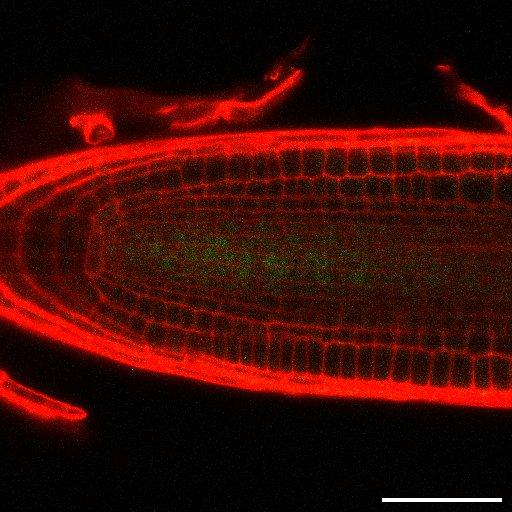

Supplement: Supplementary file 9 — Source Data Fig. 6 [file 44318_2024_71_MOESM9_ESM.zip › Fig 6/C/PHB PHB GFP in ago10-1/DAG6.jpg]
