# Supplementary material for: An AGO10:miR165/6 module regulates meristem activity and xylem development in the Arabidopsis root
Source: EMBO J. 2024 Apr 2;43(9):8. doi: 10.1038/s44318-024-00071-y (PMC11066010; doi:10.1038/s44318-024-00071-y)
Supplement: Supplementary file 12 — Expanded View Figures [file 44318_2024_71_MOESM12_ESM.pdf]

Expanded View Figures

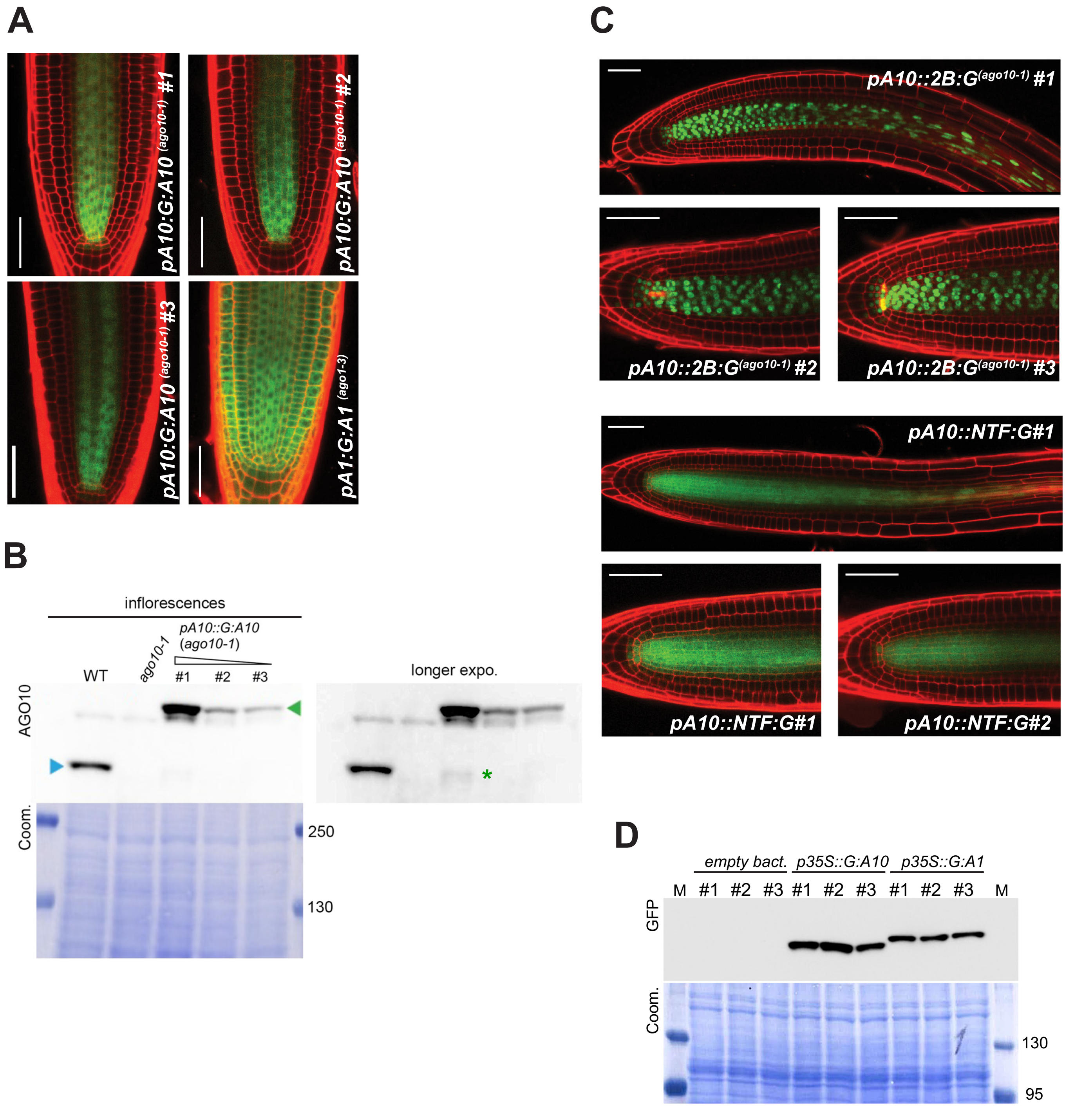

Figure EV1. AGO10 expression and transgenic lines' characterisation.

(**A**) QC-proximal views of the GFP signals yielded in root tips by the indicated reporters in the indicated genetic backgrounds. (**B**) Western analyses of GFP:AGO10 (green arrow) and endoAGO10 (blue arrow) accumulation in inflorescences where the anti-AGO10 antibody yields almost no non-specific or background signals. The longer exposure on the right-hand side allows detection of a GFP::AGO10-derived degradation fragment in overexpressor line #1, indicated with a green asterisk. Coom: Coomassie blue staining provides a total protein loading control. M: protein ladder with molecular weight in kDa. (**C**) GFP signals yielded in root tips by the indicated reporters in the indicated genetic backgrounds. All scale bars: 50 μm. cell walls were stained with PI yielding a red signal. (**D**) Transient expression of *p35S::G:A10* or *p35S::G:A1* in *N.benthamiana* leaves. Samples were collected 2 days post-infiltration and the extracted proteins subjected to western analysis using an anti-GFP antibody. Both proteins accumulate similarly. Coom: Coomassie blue staining provides a total protein loading control. M: protein ladder with molecular weight in kDa. Data information: (**A**, **C**) Roots were inspected at 6 DAG. Source data are available online for this figure.

 © The Author(s)

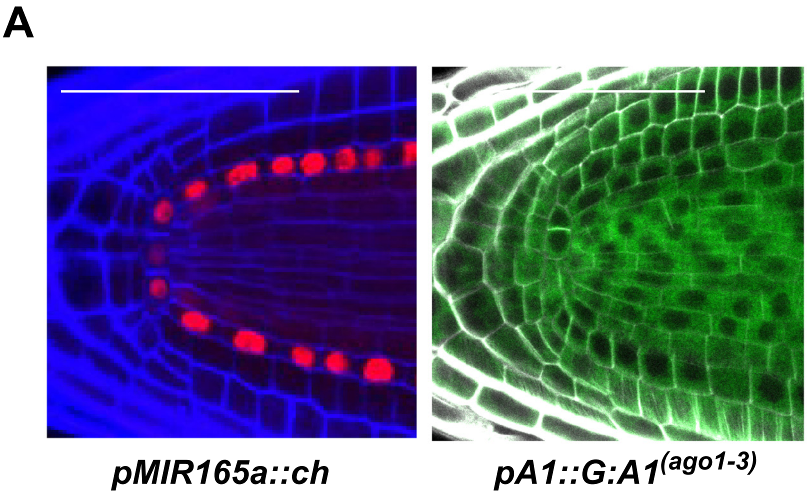

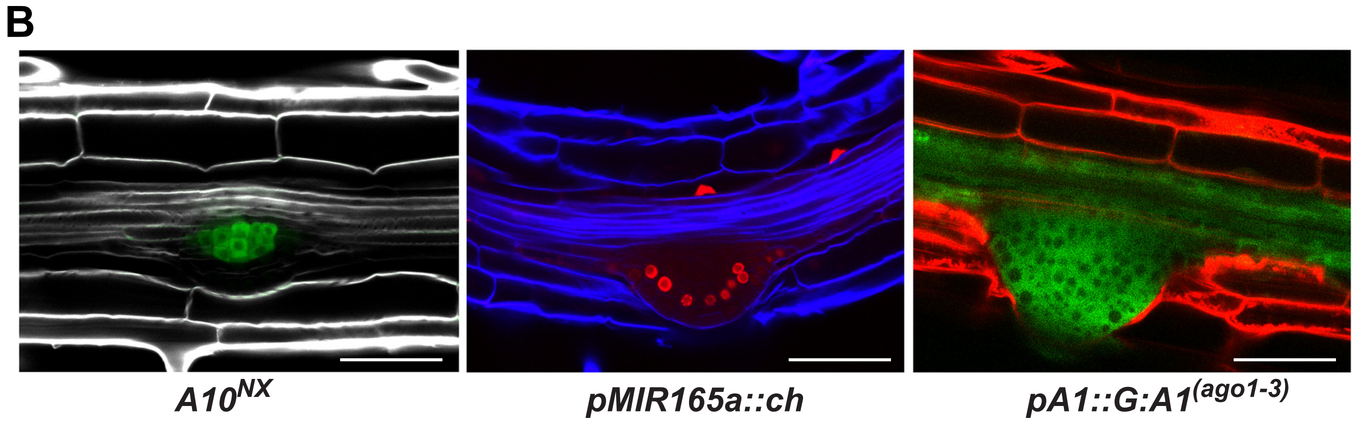

Figure EV2. ***MIR165a*** **transcription and GFP-AGO1 accumulation in the root tip.**

(**A**) QC-proximal views of the mCherry and GFP signals yielded in 6 DAG root tips of, respectively, the *pMIR165a::ch* transcriptional- and *pA1:G::A1*$^{(ago1\text{-}3)}$ translational-reporter lines. (**B**) GFP or mCherry signals yielded by the indicated reporters in lateral root initiation sites in the indicated backgrounds. Cell walls were stained by calcofluor white (in white) or PI (in red). Scale bars in (**A**, **B**): 50 µm. Source data are available online for this figure.

© The Author(s)

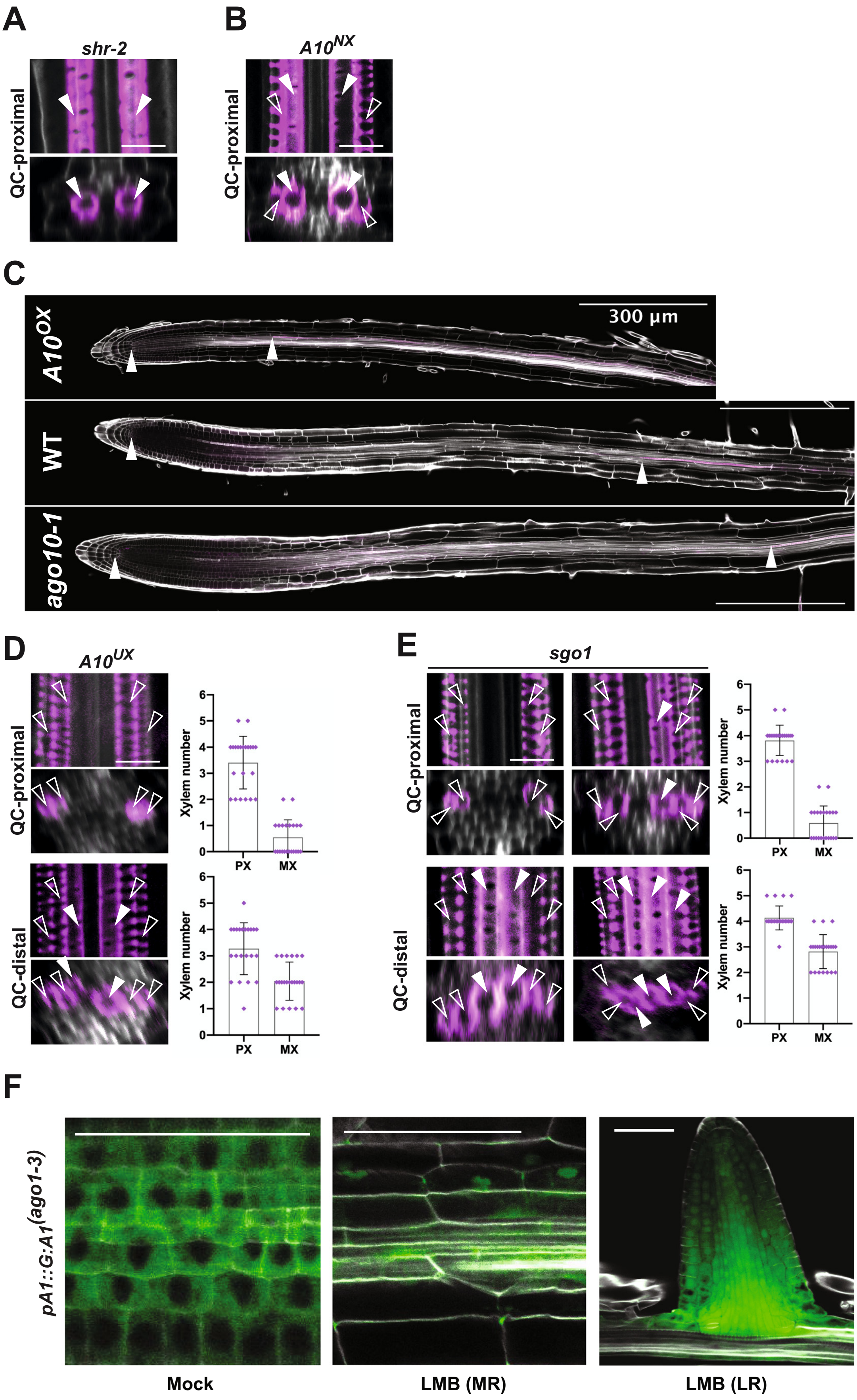
A
shr-2
QC-proximal
B
A10NX
QC-proximal
C
300 µm
A10OX
WT
ago10-1
D
A10UX
QC-proximal
QC-distal
Xylem number
PX
MX
E
sgo1
QC-proximal
QC-distal
Xylem number
PX
MX
F
pA1::G:A1(ago1-3)
Mock
LMB (MR)
LMB (LR)

 © The Author(s)

Figure EV3. Basic Fuchsin staining and meristem length in the indicated genotypes as well as LMB treatments.

(**A**, **B**) Basic Fuchsin staining of the differentiated xylem in the QC-proximal regions of the indicated genotypes. Empty arrows: PX; filled arrows: MX. Scale bars: 10 μm. (**C**) Basic Fuchsin staining to reveal the distance between the QC (left arrows) and the initiation of mature xylem cells (having gained their secondary cell wall; right arrows) in the indicated genotypes. Scale bars: 300 μm. (**D**, **E**) Basic Fuchsin staining of xylems in both QC-proximal (upper panels) and QC-distal (lower panels) root regions. The graphs on the right-hand side depict the numbers of PX and MX files detected in $n = 22$ plants at the indicated positions. Empty arrow heads: PX; filled arrow heads: MX. Scale bars: 10 μm. See Dataset EV3 for the MX-*vs*-PX scores of individual plants used to produce these graphs. Error bars: standard deviations. (**F**) Mock or vacuum-infiltrated LMB treatments within the stele of *pA1::G:A1*$^{(ago1\text{-}3)}$ main roots (MR) or lateral roots (LR). Note GFP:AGO1 relocalization from the cytosol to the nucleus upon LMB treatment. Source data are available online for this figure.

© The Author(s)

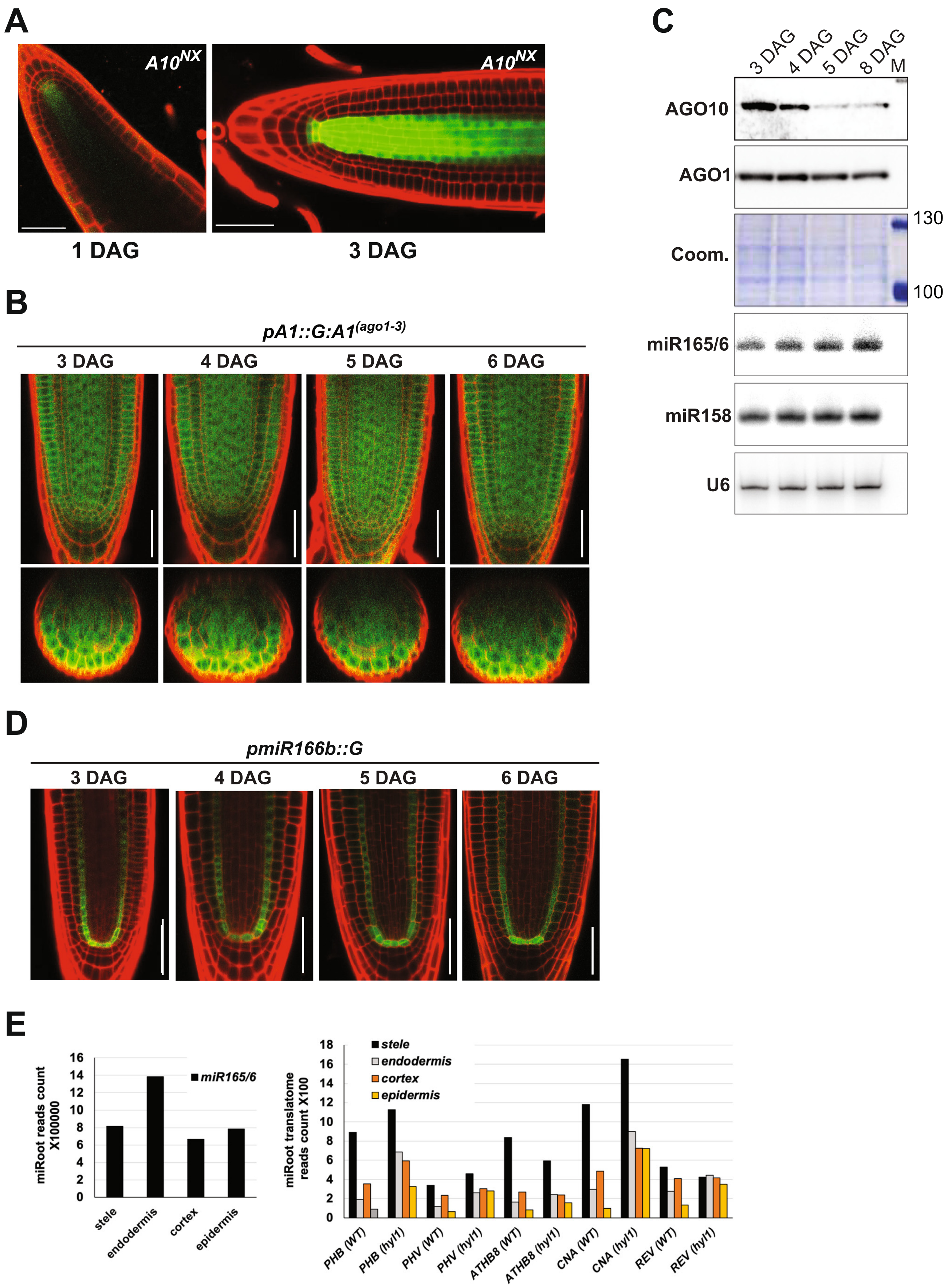
A
A10NX
A10NX
1 DAG
3 DAG
B
pA1::G:A1(ago1-3)
3 DAG
4 DAG
5 DAG
6 DAG
C
3 DAG
4 DAG
5 DAG
8 DAG
M
AGO10
AGO1
Coom.
130
100
miR165/6
miR158
U6
D
pmiR166b::G
3 DAG
4 DAG
5 DAG
6 DAG
E
miRoot reads count X100000
miR165/6
stele
endodermis
cortex
epidermis
miRoot translatome reads count X100
stele
endodermis
cortex
epidermis
PHB (WT)
PHB (hyl1)
PHV (WT)
PHV (hyl1)
ATHB8 (WT)
ATHB8 (hyl1)
CNA (WT)
CNA (hyl1)
REV (WT)
REV (hyl1)

© The Author(s)

Figure EV4. Time-course analyses between 3 and 6 days post-germination.

(**A**) Compared signal intensity yielded by the *AGO10*^{NX} reporter at 1-*vs*-3-DAG. (**B**) GFP signal yielded by the *pA1::G:A1*$^{(ago1\text{-}3)}$ reporter. (**C**) Time-course western analysis of endo-AGO1 *vs* endo-AGO10 levels (upper panels) and -northern analysis of miR165/166 vs miR158 levels (lower panels) in whole root tips of WT plants. Coom: Coomassie blue staining provides a total protein loading control. The indicated miRNAs were detected by northern blot analysis via hybridization of radiolabeled sequence-complementary oligonucleotides. Hybridization to the U6 snRNA provides a total small RNA loading control. (**D**) GFP signal yielded by the *pMIR166b::G* transcriptional reporter in the 3-to-6 DAG germination time-course (**E**) miRoot layer-specific reads count for miR165/166 loading into AGO1 (left) and layer-specific translatome analysis of all root-expressed members of the *HD-ZIP III* family in the WT- or miRNA-deficient *hyl1* background (middle). Data information: (**A**) experiment conducted in the main root tip under identical laser settings. (**B**, **D**) seedlings were grown under conditions identical to those used in the 3-to-6 DAG germination time-course of Fig. 6A,C. The same laser settings were applied in acquiring images. Scale bars in (**A**, **B**, **D**): 50 μm. Source data are available online for this figure.

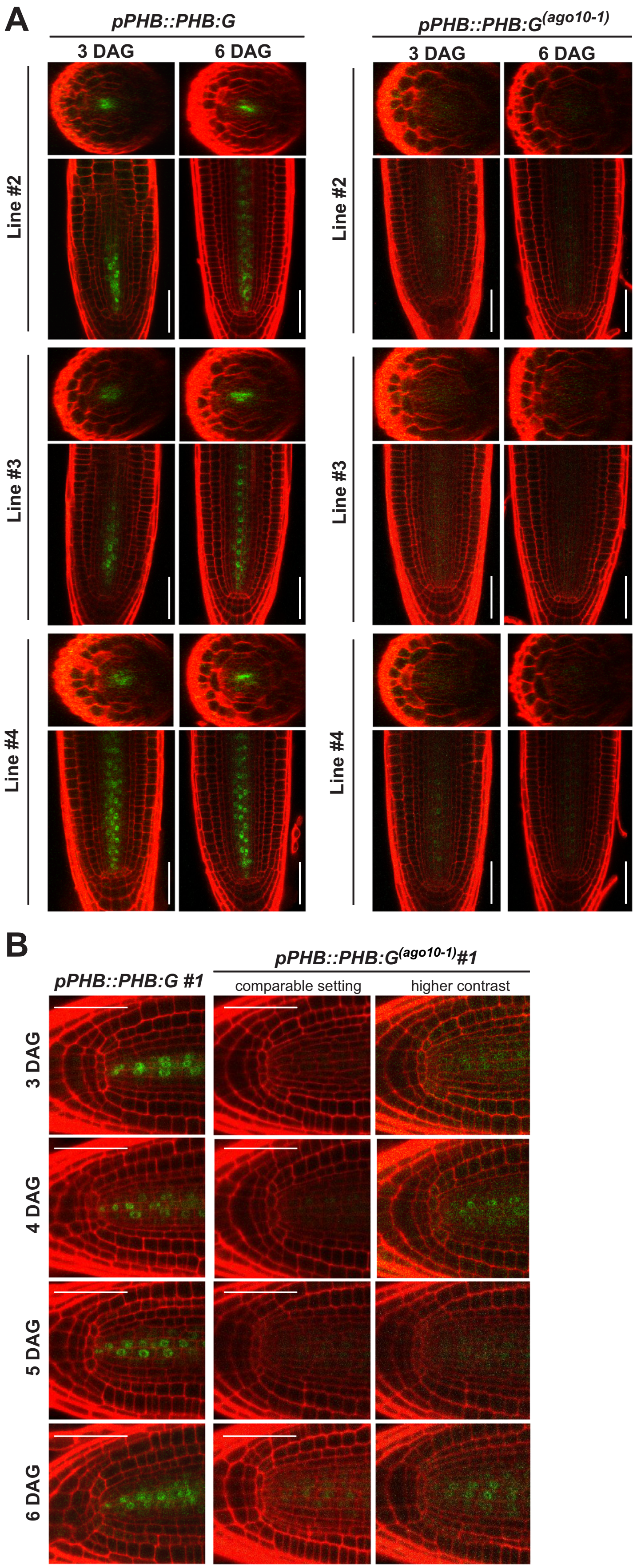
A
pPHB::PHB:G
3 DAG
6 DAG
pPHB::PHB:G(ago10-1)
3 DAG
6 DAG
Line #2
Line #3
Line #4
Line #2
Line #3
Line #4
B
pPHB::PHB:G #1
pPHB::PHB:G(ago10-1)#1
comparable setting
higher contrast
3 DAG
4 DAG
5 DAG
6 DAG

 © The Author(s)

Figure EV5. Characterisation of independent lines of pPHB::PHB:G in the WT or *ago10-1* background.

(**A**) Longitudinal and radial views of roots of additional independent T2 lines of *pPHB::PHB:G* in either the WT (lines #2, 3 and 4) or *ago10-1* (lines #2, 3 and 4) mutant background at 3 or 6 DAG. Confocal settings were identical to those used in Fig. 6. Scale bars: 50 µm. (**B**) QC-proximal views of the root tips of lines *pPHB::PHB:G #1* and *pPHB::PHB:G*$^{(ago10\text{-}1)}$ *#1* at 3, 4, 5 or 6 DAG. The right panels show *pPHB::PHB:G*$^{(ago10\text{-}1)}$ *#1* under normal and enhanced contrast settings to compare to the signal from *pPHB::PHB:G #1*, revealing the wider repartition of PHB in the stele of *ago10-1* background despite substantially reduced levels. Scale bars: 50 µm. Source data are available online for this figure.

© The Author(s)
